# Supplementary material for: Aryl Hydrocarbon Receptor Contributes to the Transcriptional Program of IL-10-Producing Regulatory B Cells
Source: Cell Rep. 2019 Nov 12;29(7):1878–1892.e7. doi: 10.1016/j.celrep.2019.10.018 (PMC6856759; doi:10.1016/j.celrep.2019.10.018)
Supplement: Document S1. Figures S1–S7 and Tables S1 and S2 [file mmc1.pdf]

Cell Reports, Volume *29*

Supplemental Information

Aryl Hydrocarbon Receptor Contributes to the Transcriptional Program of IL-10-Producing Regulatory B Cells

Christopher J.M. Piper, Elizabeth C. Rosser, Kristine Oleinika, Kiran Nistala, Thomas Krausgruber, André F. Rendeiro, Aggelos Banos, Ignat Drozdov, Matteo Villa, Scott Thomson, Georgina Xanthou, Christoph Bock, Brigitta Stockinger, and Claudia Mauri

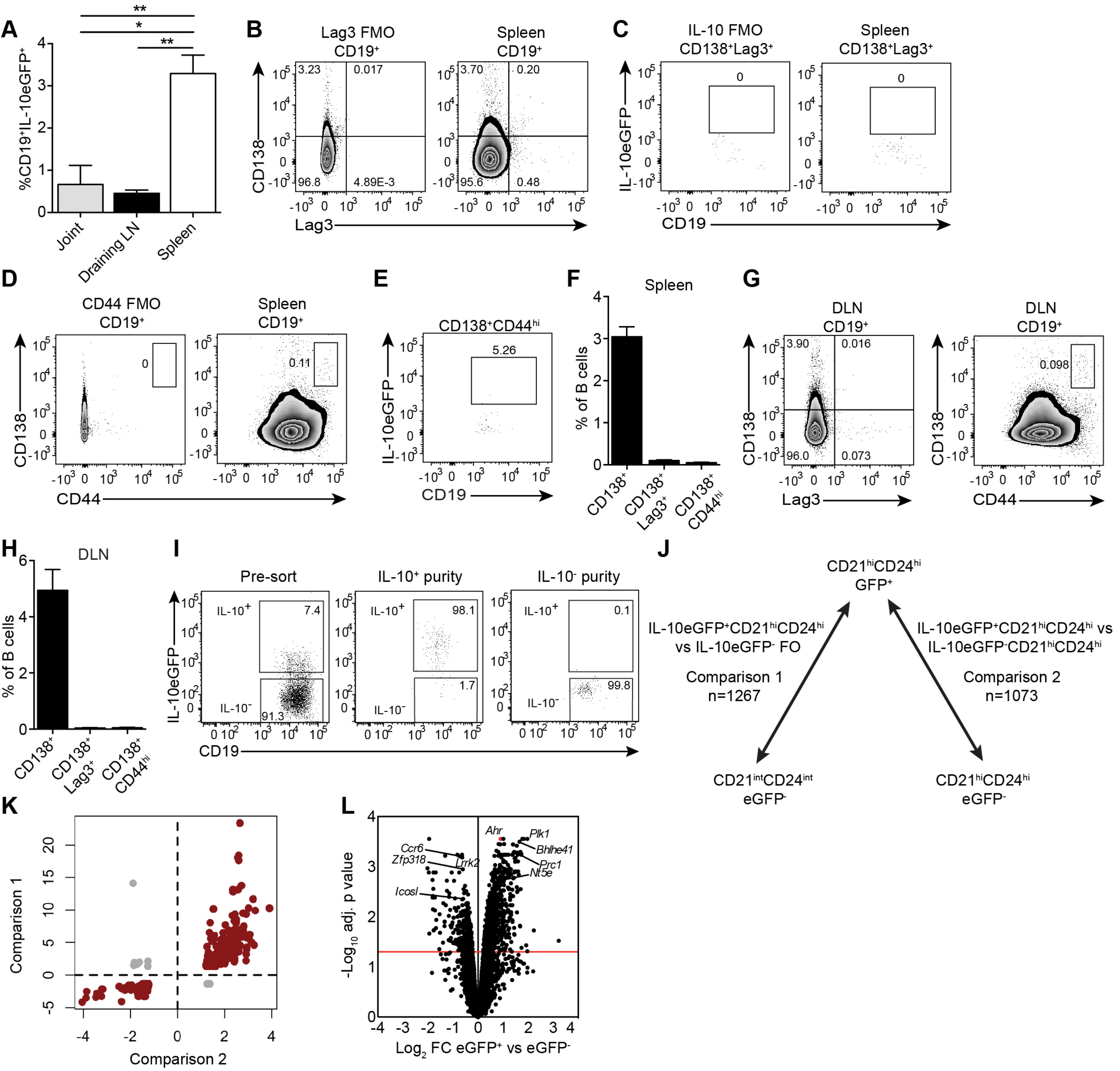

Figure S1. Comparison of gene expression profiles between eGFP^{+} and eGFP^{-} subsets. Related to Figures 1 and 2. Antigen-induced arthritis (AIA) was induced in IL-10eGFP reporter (Vert-X) mice. (**A**) Bar chart showing the frequencies of IL-10^{+}CD19^{+} B cells in the joint, draining LN and spleens of Vert-X mice (n=3). **B-E,** Representative flow cytometry plots showing respectively the frequencies of (**B**) CD138^{+}Lag3^{+} plasmablasts, (**C**) IL-10^{+} CD138^{+}Lag3^{+}, (**D**) CD138^{+}CD44^{hi} plasmablasts and (**E**) IL-10^{+}CD138^{+}CD44^{hi} B plasmablasts in the spleen. (**F**) Bar chart showing the percentages of CD19^{+}CD138^{+}, CD19^{+}CD138^{+}Lag3^{+} and CD19^{+}CD138^{+}CD44^{hi} plasmablasts in the spleens of in Vert-X mice, as shown gated in **B+D** (n=5). (**G**) Representative flow cytometry plots showing respectively the frequencies of (left) CD138^{+}Lag3^{+} and (right) CD138^{+}CD44^{hi} plasmablasts in the DLNs of Vert-X mice. (**H**) Bar chart showing the percentages of CD19^{+}CD138^{+}, CD19^{+}CD138^{+}Lag3^{+} and CD19^{+}CD138^{+}CD44^{hi} plasmablasts in the DLNs of in Vert-X mice, as shown gated in **G** (n=5). (**I**) Representative flow cytometry plots showing purity of CD19^{+}CD21^{hi}CD24^{hi}eGFP^{+} and CD19^{+}CD21^{hi}CD24^{hi}eGFP^{-} B cells. (**J**) Total number of differentially expressed genes between CD19^{+}CD21^{hi}CD24^{hi}GFP^{+} and IL-10eGFP^{-} subsets (>1.5 fold change, adjusted p value <0.05). (**K**) Scatter plot showing fold changes of differentially expressed genes from the two comparisons (n=660). Concordant changes for both comparisons are shown in red, and discordant in grey. (**L**) Volcano plot analysis showing $\log_2$ fold changes (FC) between CD19^{+}CD21^{hi}CD24^{hi}eGFP^{+} B cells versus CD19^{+}CD21^{hi}CD24^{hi}eGFP^{-} B cells, plotted against -$\log_{10}$ adjusted p value. *Ahr* is highlighted in red (adjusted p value of 3.4E-05). All experiments were carried out at day 7 post IA-injection. For figures **A-H,** data representative of 2 independent experiments. Figures **A, F and H,** data are expressed as mean±sem. *p<0.05, **p<0.01, one-way ANOVA.

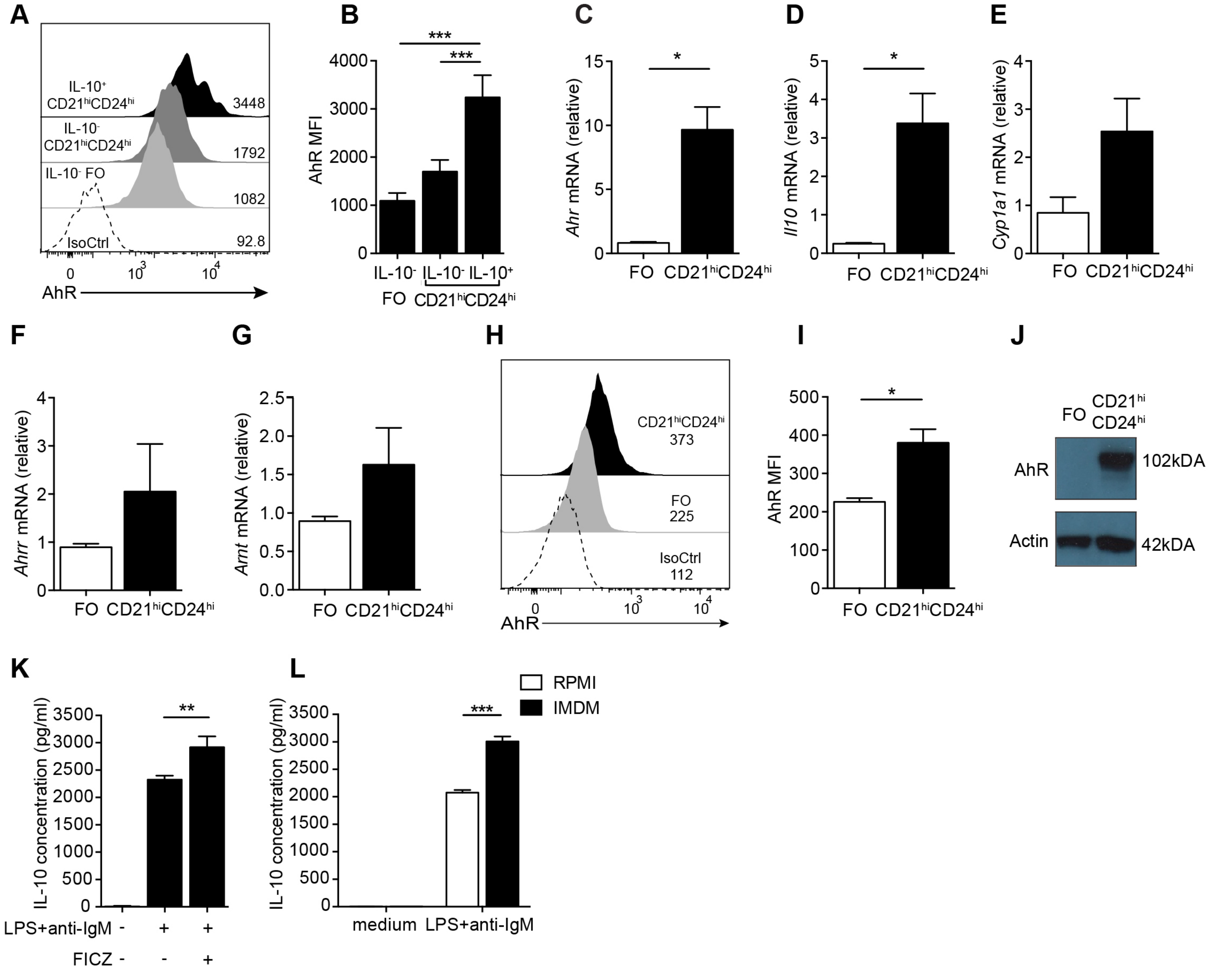

Figure S2. AhR is upregulated in IL-10⁺CD19⁺CD21ʰⁱCD24ʰⁱB cells after stimulation with LPS+anti-IgM. Related to Figure 2 and Figure 3. (**A**) Representative histogram and (**B**) bar chart showing the MFI of AhR expression in IL-10^{+}CD19^{+}CD21^{hi}CD24^{hi}, IL-10^{-}CD19^{+}CD21^{hi}CD24^{hi} and IL-10^{-}FO B cells after 48h stimulation with LPS+anti-IgM (*n*=4). **C-J, Increased levels of *Ahr* and downstream pathway in *ex vivo* CD19^{+}CD21^{hi}CD24^{hi} compared to FO B cells.** CD19^{+}CD21^{hi}CD24^{hi} and FO B cells were isolated from WT mice and the mRNA levels of (**C**) *Ahr*, (**D**) *Il10*, (**E**) *Cyp1a1*, (**F**) *Ahrr* and (**G**) *Arnt* were analysed *ex-vivo* (*n*=3). (**H**) Representative histogram and (**I**) bar chart showing the median fluorescent intensity (MFI) of AhR expression in CD19^{+}CD21^{hi}CD24^{hi} and FO B cells *ex vivo* (*n*=4). (**J**) Western blot showing the expression of AhR in CD19^{+}CD21^{hi}CD24^{hi} and FO B cells isolated from arthritic WT mice. β-actin was used as a loading control. The numbers indicate the size of the protein bands in kDA. **K-L, AhR agonists increase IL-10 concentration in LPS+anti-IgM stimulated CD19^{+}CD21^{hi}CD24^{hi}B cells.** (**K**) CD19^{+}CD21^{hi}CD24^{hi}B cells were cultured in RPMI media for 48h with LPS+anti-IgM±FICZ and IL-10 was measured in the supernantant (*n*=4). (**L**) CD19^{+}CD21^{hi}CD24^{hi}B cells were cultured in LPS+anti-IgM for 48h in RPMI or IMDM media and IL-10 was measured in the supernatants (*n*=5). For qPCR, gene expression was calculated normalizing to β-Actin. All experiments were carried out at day 7 post IA-injection. Data representative of at least 2 independent experiments with biological replicates. Figures **B-G, I and K-L** data are expressed as mean±sem. *$p<0.05$, **$p<0.01$, ***$p<0.001$, one and two-way ANOVA and unpaired t test.

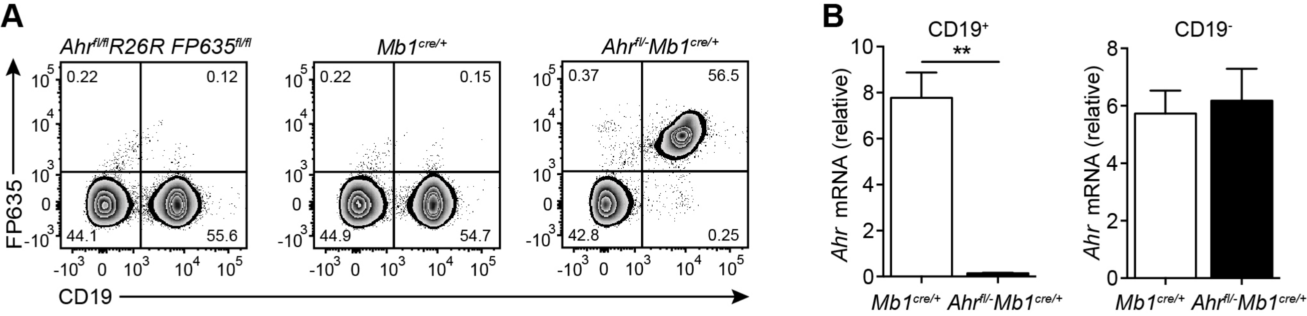

Figure S3. Validation of B cell AhR deficient (*Ahr*$^{fl/-}$*Mb1*$^{cre/+}$) mice. Related to Figures 4-6. (**A**) *Ahr*$^{fl/-}$*Mb1*$^{cre/+}$ mice lack *Ahr* in Mb1-expressing cells and report Cre activity via FP635 expression. Representative flow cytometry plots of FP635 expression in the parental *Ahr*$^{fl/fl}$*R26R FP635*$^{fl/fl}$ strain, *Mb1*$^{cre/+}$ control mice and *Ahr*$^{fl/-}$*Mb1*$^{cre/+}$ mice. (**B**) Splenocytes from *Ahr*$^{fl/-}$*Mb1*$^{cre/+}$ mice and *Mb1*$^{cre/+}$ controls were sorted into CD19^{+}B220^{+} and CD19^{-}B220^{-} fractions and the levels of *Ahr* mRNA were analysed *ex-vivo* (*n*=3). For qPCR, gene expression was calculated normalizing to β-Actin. All experiments were carried out at day 7 post IA-injection. Data representative of at least 2 independent experiments with biological replicates. Figure **B,** data are expressed as mean±sem. $^{**}p<0.01$, unpaired t test.

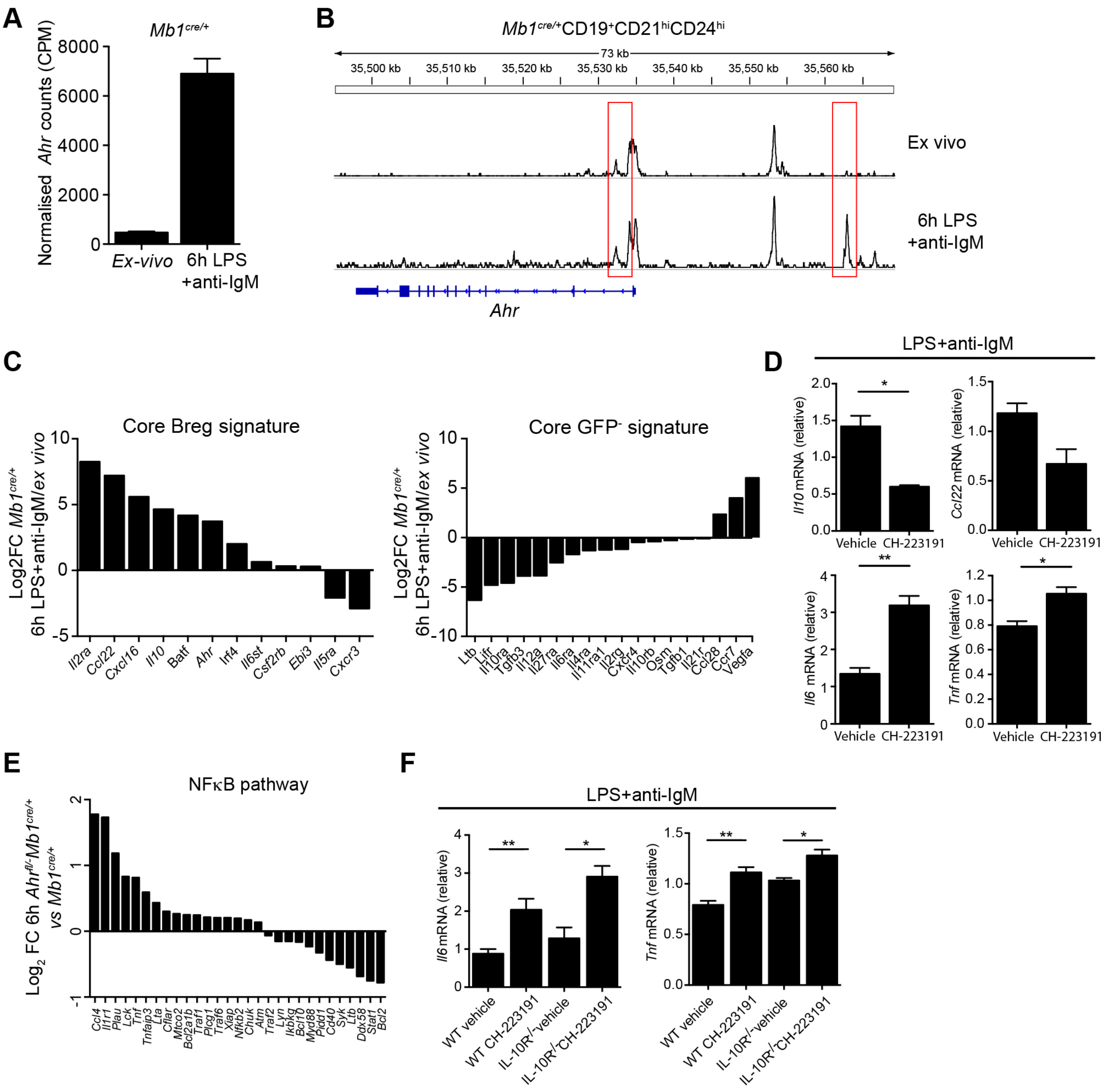

Figure S4. AhR contributes to the chromatin and transcriptional landscape of CD19^{+}CD21^{hi}CD24^{hi}B cells after Breg priming conditions. Related to Figure 4. (**A**) Normalised counts (CPM) of *Ahr* expression in *Mb1*$^{cre/+}$ mice *ex vivo* and after activation for 6h with LPS+anti-IgM. (**B**) Representative track of the *Ahr* locus before and after stimulation with LPS+anti-IgM in *Mb1*$^{cre/+}$ CD19^{+}CD21^{hi}CD24^{hi} B cells. Red box indicates one significantly differentially accessable region. (**C**) Log$_2$ FC for core GFP^{+} and GFP^{-} gene signatures (identified from Figure 1G) comparing 6h LPS+anti-IgM vs *ex vivo* *Mb1*$^{cre/+}$ CD19^{+}CD21^{hi}CD24^{hi}B cells. (**D**) CD19^{+}CD21^{hi}CD24^{hi}B cells were isolated from WT mice and stimulated for 24h with LPS+anti-IgM in the presence of the AhR antagonist (CH-223191) or a vehicle control and *Il10, Ccl22, Il6* and *Tnf* mRNA levels were analyzed (n=5). (**E**) Log$_2$ FC for NF-κB pathway genes (taken from KEGG) comparing 6h LPS+anti-IgM stimulated CD19^{+}CD21^{hi}CD24^{hi}B cells from *Mb1*$^{cre/+}$ and *Ahr*$^{fl/-}$*Mb1*$^{cre/+}$ mice. (**F**) WT or IL-10R$^{-/-}$ CD19^{+}CD21^{hi}CD24^{hi}B cells were cultured with LPS+anti-IgM±CH-223191 and *Il6* and *Tnf* mRNA levels were analyzed (n=5). For qPCR, gene expression was calculated normalizing to β-Actin. All experiments were carried out at day 7 post IA-injection. For RNA-seq data, n=3 per condition and genotype. For ATAC-seq data, n=3 for *Mb1*$^{cre/+}$ mice and n=2 for *Ahr*$^{fl/-}$*Mb1*$^{cre/+}$ mice. Figures **A, D and F,** data expressed as mean±sem. *p<0.05, **p<0.01, Mann-whitney test and two-way ANOVA. Figures **D** and **F,** representative of two independent experiments with biological replicates.

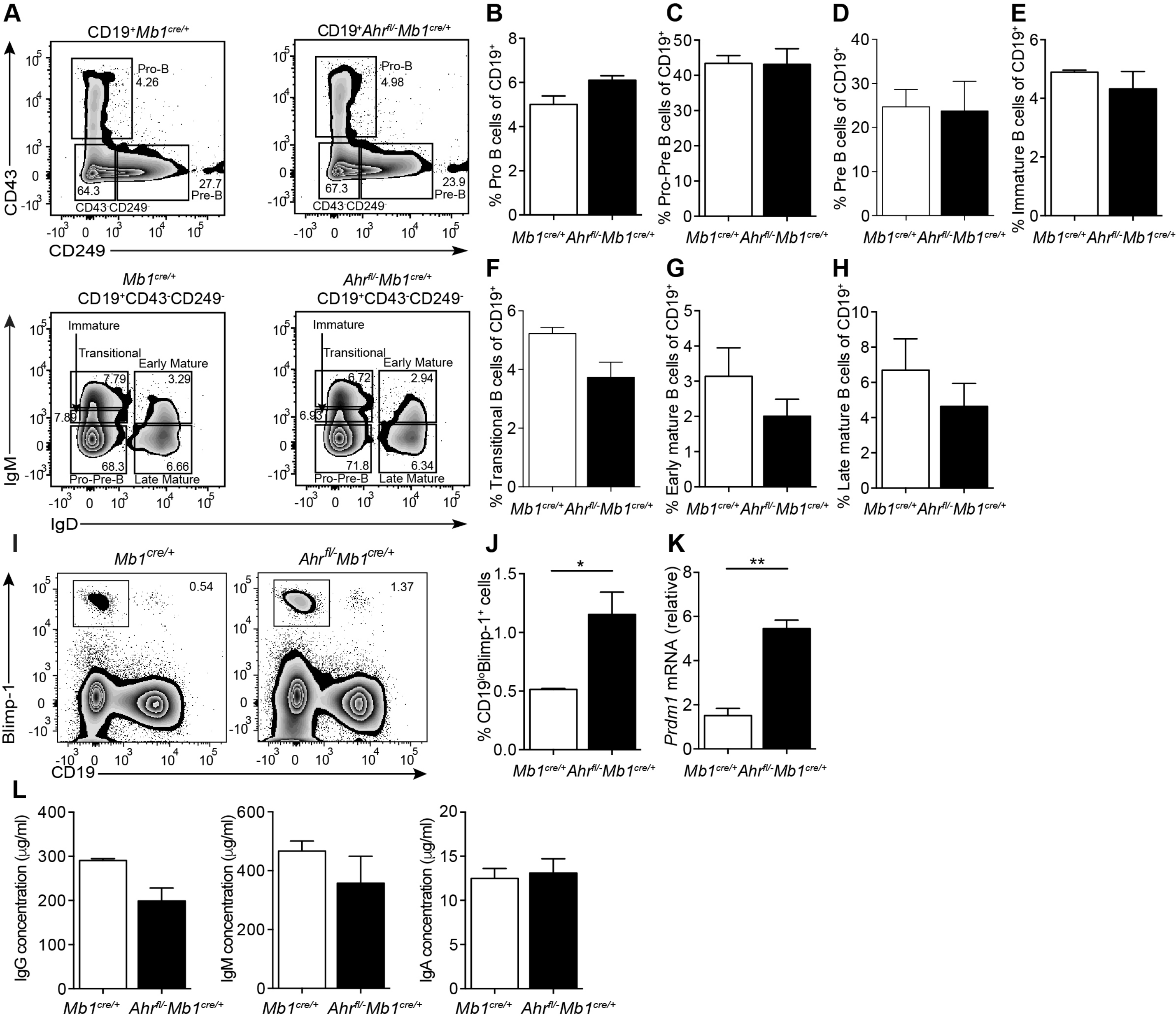

Figure S5. A-H, AhR plays a redundant role in early B cell development in the bone marrow. Related to Figure 6. (**A**) Representative flow cytometry plots showing $Mb1^{cre/+}$ and $Ahr^{fl/-}Mb1^{cre/+}$ B cell subsets in the bone marrow. **B-H,** Bar charts showing the frequencies of (**B**) pro, (**C**) pro-pre, (**D**) pre, (**E**) immature, (**F**) transitional (**G**) early mature and (**H**) late mature B cells, as a percentage of total $CD19^+$ B cells in the bone marrow for $Mb1^{cre/+}$ and $Ahr^{fl/-}Mb1^{cre/+}$ mice (*n*=3 per genotype). **I-K, AhR represses plasma cell differentiation.** (**I**) Representative flow cytometry plots and (**J**) bar chart showing the percentage of splenic $Blimp\text{-}1^+$ B cells from $Mb1^{cre/+}$ and $Ahr^{fl/-}Mb1^{cre/+}$ mice (*n*=3). (**K**) Total splenic B cells were isolated from $Mb1^{cre/+}$ and $Ahr^{fl/-}Mb1^{cre/+}$ mice and *Prdm1* mRNA levels were analysed *ex-vivo* (*n*=3). (**L**) Serum concentrations of total IgG, IgM and IgA from $Mb1^{cre/+}$ and $Ahr^{fl/-}Mb1^{cre/+}$ mice were measured by ELISA. For qPCR, gene expression was calculated normalizing to β-Actin. All experiments were carried out at day 7 post IA-injection. Data representative of at least 2 independent experiments with biological replicates. Figures **B-H, and J-L** data are expressed as mean±sem. *$p<0.05$, **$p<0.01$, unpaired t test.

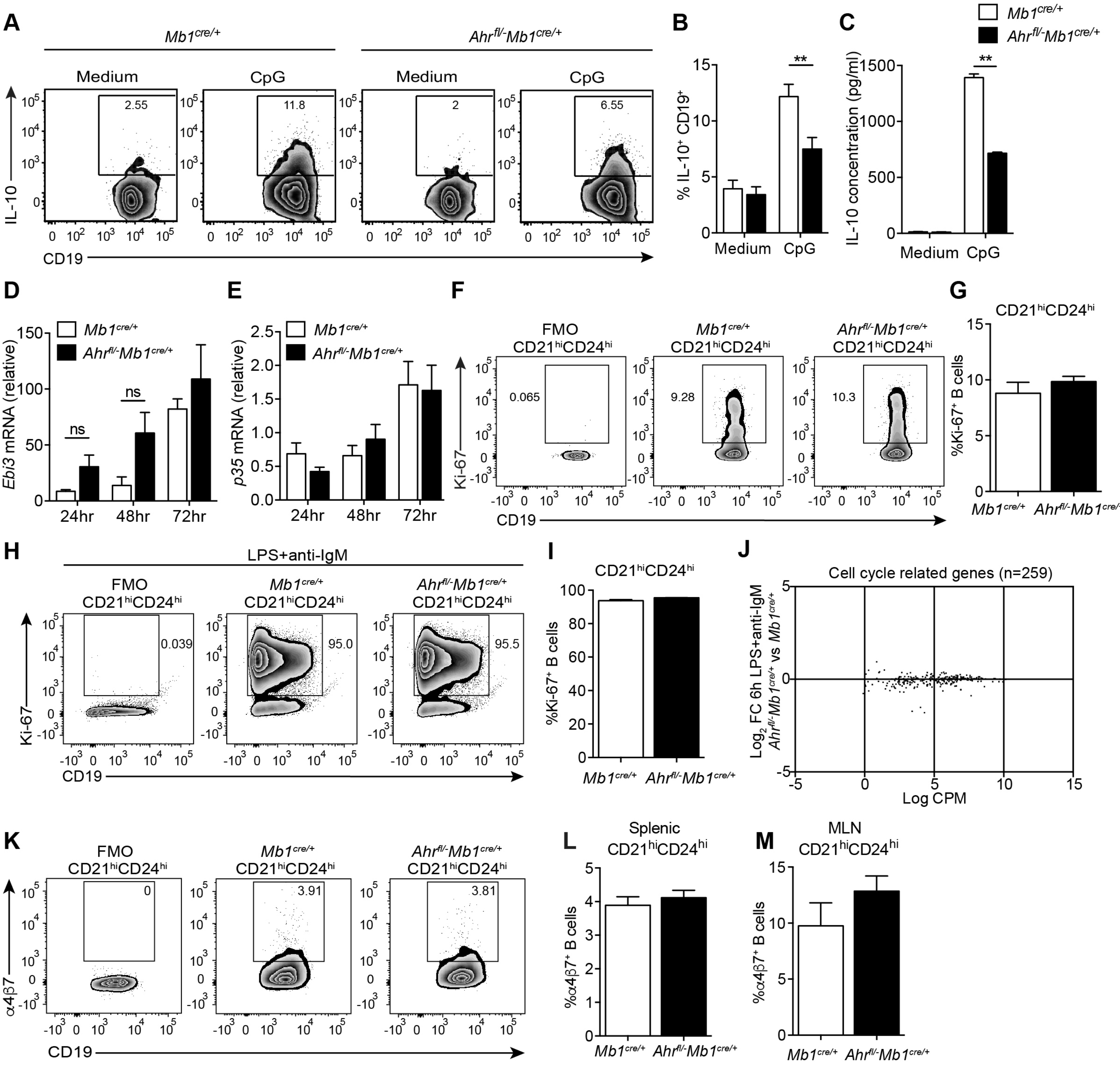

Figure S6. A-C, AhR is required for IL-10 production by Breg *in vitro*. Related to Figure 6. Representative flow cytometry plots (**A**) and bar chart (**B**) showing the percentage of IL-10-expressing $CD19^{+}$B cells from *$Mb1^{cre/+}$* and *$Ahr^{fl/-}Mb1^{cre/+}$* mice, after 48h stimulation with CpGb (*n*=3). (**C**) IL-10 production, as measured by ELISA (*n*=3). **D-E, AhR does not control IL-35 production by B cells.** Splenic B cells were isolated from *$Mb1^{cre/+}$* and *$Ahr^{fl/-}Mb1^{cre/+}$* mice and stimulated with LPS for the indicated times and (**D**) *Ebi3* and (**E**) *p35* mRNA levels were analysed (*n*=3). **F-J, AhR does not affect the proliferation of $CD19^{+}CD21^{hi}CD24^{hi}$B cells in arthritic mice.** (**F**) Representative flow cytometry plots and (**G**) bar graphs summarising Ki-67 expression in $CD19^{+}CD21^{hi}CD24^{hi}$B cells from *$Mb1^{cre/+}$* and *$Ahr^{fl/-}Mb1^{cre/+}$* mice *ex vivo* after day 7 AIA and (**H-I**) after 48h stimulation with LPS+anti-IgM (*n*=3). (**J**) Volcano plot (RNA-seq analysis) showing $\log_2$ fold changes (FC) between 6h LPS+anti-IgM stimulated $CD19^{+}CD21^{hi}CD24^{hi}$B cells from *$Ahr^{fl/-}Mb1^{cre/+}$* versus *$Mb1^{cre/+}$* mice, plotted against average log counts per million (CPM; across all samples) for cell cyle related genes (n=259). **K-M, α4β7 is not differentially expressed between *$Mb1^{cre/+}$* and *$Ahr^{fl/-}Mb1^{cre/+}$* $CD19^{+}CD21^{hi}CD24^{hi}$B cells.** (**K**) Representative flow cytometry plots of splenic α4β7 expression in $CD19^{+}CD21^{hi}CD24^{hi}$B cells from *$Mb1^{cre/+}$* and *$Ahr^{fl/-}Mb1^{cre/+}$* mice. **L-M,** Bar charts showing the frequencies of α4β7-expressing $CD19^{+}CD21^{hi}CD24^{hi}$B cells in the (**L**) spleen and (**M**) MLNs of *$Mb1^{cre/+}$* and *$Ahr^{fl/-}Mb1^{cre/+}$* mice (*n*=6). All experiments were carried out at day 7 post IA-injection. For qPCR gene expression was calculated normalising to β-Actin. All data representative of at least 2 independent experiments, with biological replicates. Figures **B-E, G, I and L-M,** data expressed as mean±sem. **$p<0.01$, two-way ANOVA.

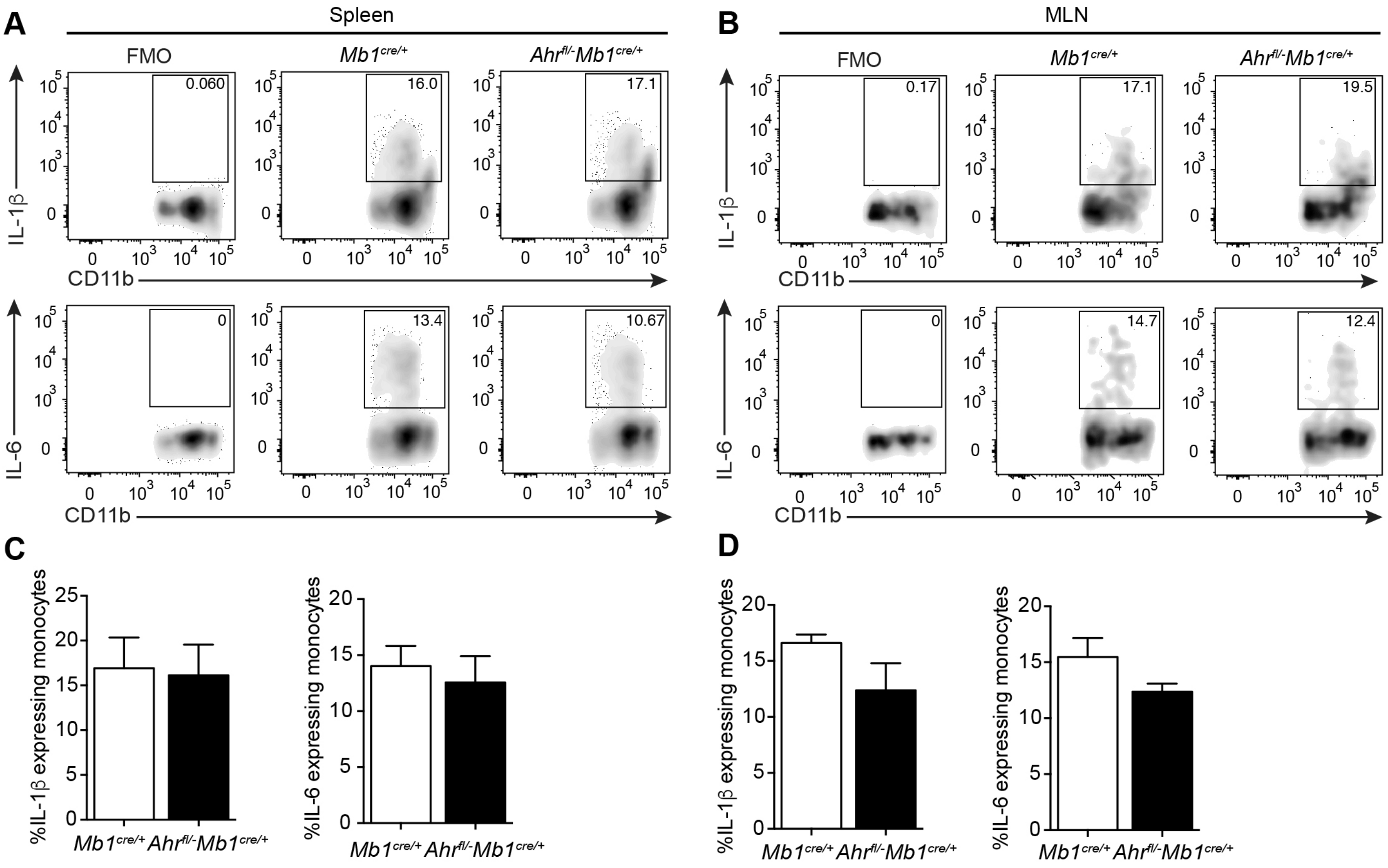

Figure S7 (Related to Figure 6): A-D, No difference in monocyte IL-1β and IL-6 expression is observed between *Mb1*$^{cre/+}$ and *Ahr*$^{fl/-}$*Mb1*$^{cre/+}$ mice. Total splenocytes or MLN cells were cultured for 6h with LPS. **A-D,** Representative flow cytometry plots and bar charts showing respectively the percentage of (**A,C**) splenic and (**B,D**) MLN IL-1β and IL-6-expressing monocytes (*n*=5). All data representative of at least 2 independent experiments with biological replicates. Figures **C-D,** data expressed as mean±sem. $^{**}p<0.01$

Symbol	Name	Function	FC (CD21^hiCD24^hi pos vs CD21^hiCD24^hi neg)	adj.P.Val (CD21^hiCD24^hi pos vs CD21^hiCD24^hi neg)	FC (CD21^hiCD24^hi pos vs FO)	adj.P.Val (CD21^hiCD24^hi pos vs FO)
Ahr	Aryl-hydrocarbon receptor	DNA binding	1.869114565	5.05135E-05	5.183739908	1.73543E-08
E2f8	E2F transcription factor 8	Core promoter binding	3.524122031	8.24144E-05	9.937468163	1.16556E-07
Bhlhe41	Basic helix-loop-helix family, member e41	RNA polymerase II core promoter proximal region sequence-specific DNA binding	3.151182551	5.20865E-05	5.070827978	2.34121E-07
Pim1	Proviral integration site 1	Nucleotide binding	1.43710652	0.000803313	1.536952061	5.28079E-05
Tacc3	Transforming, acidic coiled-coil containing protein 3	Protein binding	1.754219566	0.000237504	2.612848846	1.13543E-06
E2f7	E2F transcription factor 7	Core promoter binding	1.569164933	0.000491558	2.089170639	3.35506E-06
Dnmt1	DNA methyltransferase (cytosine-5) 1	DNA binding	1.525927763	0.001016079	1.542642041	0.000192334
Zbtb32	Zinc finger and BTB domain containing 32	Nucleic acid binding	1.564102074	0.00060818	1.599906949	8.54549E-05
Zfpm1	Zinc finger protein, multitype 1	RNA polymerase II core promoter binding transcription factor activity	1.635156815	0.000956596	2.073406035	1.36055E-05
Pmf1	Polyamine-modulated factor 1	Transcription coactivator activity	1.613117282	0.000743998	2.03500123	9.61821E-06
C1qbp	C1q binding protein	Complement component C1q binding	1.49365815	0.002166338	1.796509212	4.16981E-05
Foxm1	Forkhead box M1	DNA binding	1.457277204	0.00190059	2.20317842	3.01442E-06

Cenpf	Centromere protein F	Protein C-terminus binding	1.922637351	0.008852351	3.81234672	2.613E-05
Pdlim1	PDZ and LIM domain 1 (elfin)	Transcription coactivator activity	1.538439454	0.001738439	1.372627344	0.00277832
Setd8	SET domain containing (lysine methyltransferase) 8	P53 binding	1.481768428	0.005872492	1.58624982	0.000660724
E2f1	E2F transcription factor 1	Core promoter binding	1.27720107	0.005234129	1.799717751	4.40902E-06
Hes6	Hairy and enhancer of split 6	DNA binding	1.26830407	0.004558499	1.367381679	0.000227666
Smarca4	SWI/SNF related, matrix associated, actin dependent regulator of chromatin	Nucleotide binding	1.335143927	0.011950492	1.398775214	0.00168523
Dip2c	DIP2 disco-interacting protein 2 homolog C (Drosophila)	Unknown	1.258003811	0.02122796	1.734098658	2.86597E-05
Skil	SKI-like	Chromatin binding	-1.201268078	0.022180352	-1.54986752	3.18895E-05
Hhex	Hematopoietically expressed homeobox	DNA binding	-1.235422164	0.026407274	-1.511651527	0.000170022
Rbpms	RNA binding protein gene with multiple splicing	Nucleotide binding	-1.326003662	0.040745097	-1.992794467	7.70317E-05
Hist1h4k	Histone Cluster 1 H4 Family Member K	Unknown	1.274	0.019929	1.5213	0.00027

Table S1 (Related to Figure 2): List of 23 candidate genes differentially expressed between $CD21^{hi}CD24^{hi}IL\text{-}10eGFP^{+}$ and GFP^{-} populations. Abbreviations: FC – fold change, FO – Follicular.

Resource	Source	Identifier
**qPCR primers**		
Actb	ThermoFisher Scientific; This paper	N/A
Fwd 5'-AGATGACCCAGATCATGTTTGAG		
Rev 5'-AGGTCCAGACGCAGGATG		
Ahr	ThermoFisher Scientific; This paper	N/A
Fwd 5'-AGGATCGGGGTACCAGTTCA-3'		
Rev 5'-CTCCAGCGACTGTGTTTTGC-3'		
Ahrr	Qiagen	Cat#QT00161693
N/A		
Arnt	Qiagen	Cat#QT00151718
N/A		
Ccl22	ThermoFisher Scientific; Hao *et al.*, 2016	N/A
Fwd 5'-CAGGCAGGTCTGGGTGAA-3'		
Rev 5'-TAAAGGTGGCGTCGTTGG-3'		
Cyp1a1	Qiagen	Cat#QT00105756
N/A		
Ebi3	ThermoFisher Scientific; Shen et al., 2014	N/A
CGGTGCCCTACATGCTAAAT		
GCGGAGTCGGTACTTGAGAG		
Il2	ThermoFisher Scientific; Martins., 2008	N/A
5′-AGCAGCTGTTGATGGACCTA-3′		
5′-CGCAGAGGTCCAAGTTCAT-3′		
Il5ra	ThermoFisher Scientific; This paper	N/A
Fwd 5'-GGTCCCGGTATGCAGTTCTA-3'		
Rev 5'-AGCCGAATGCTGGAAAAGTG-3'		
Il6	ThermoFisher Scientific; This paper	N/A
Fwd 5'-GCCTTCTTGGGACTGATGCT-3'		
Rev 5'-TGCCATTGCACAACTCTTTTC-3'		
Il10	ThermoFisher Scientific; Yanaba et al., 2009	N/A
Fwd 5'-GGTTGCCAAGCCTTATCGGA-3'		
Rev 5'-ACCTGCTCCACTGCCTTGCT-3'		
p35	ThermoFisher Scientific; Shen et al., 2014	N/A
Fwd 5'-CATCGATGAGCTGATGCAGT-3'		
Rev 5'-CAGATAGCCCATCACCCTGT-3'		
Tnf	ThermoFisher Scientific; Denaes et al., 2016	N/A
Fwd 5'-AATGGCCTCCCTCTCATCAGTT-3'		
Rev 5'-CCACTTGGTGGTTTGCTACGA-3'		
**ChIP qPCR primers**		

***Il10* -3.5kb**<br>Fwd 5'-AGGGCTTGATAACGTGTGAGT-3'<br>Rev 5'-TGAACTTCACACCCAGCTTGAG-3'	ThermoFisher Scientific; This paper	N/A
***Il10* -2kb**<br>Fwd 5'-TAAGAGGTGCTGCTTCTCCTG-3'<br>Rev 5'-TGGCACTGGACAGTTCTATGA-3'	ThermoFisher Scientific; This paper	N/A
***Il10* -0.5kb**<br>Fwd 5'-AGGGAGGAGGAGCCTGAATAA-3'<br>Rev 5'-CCTGTTCTTGGTCCCCCTTTT-3'	ThermoFisher Scientific; This paper	N/A
***Il10* +2kb**<br>Fwd 5'-GCCACATGCATCCAGAGACAC-3'<br>Rev 5'-GTGCCTCAAAGTCACTCCCAC-3'	ThermoFisher Scientific; This paper	N/A
***Cyp1a1* -3.6kb**<br>Fwd 5'-GCTCTTTCTCTGCCAGGTTG-3'<br>Rev 5'-GGCTAAGGGTCACAATGGAA-3'	ThermoFisher Scientific; This paper	N/A
***Cyp1a1* promoter**<br>Fwd 5'-AAGCATCACCCTTTGTAGCC-3'<br>Rev 5'-CAGGCAACACAGAGAAGTCG-3'	ThermoFisher Scientific; This paper	N/A
***Gapdh* promoter**<br>Fwd 5'-GCGCGAAAGTAAAGAAAGAAGCCC-3'<br>Rev 5'-AGCGGCCCGGAGTCTTAAGTATTAG-3'	ThermoFisher Scientific; This paper	N/A

Table S2. qPCR and ChIP qPCR primers used in this study. Related to the Key Resources Table in STAR methods. N/A – not applicable.
