# Supplementary material for: Aryl Hydrocarbon Receptor Contributes to the Transcriptional Program of IL-10-Producing Regulatory B Cells
Source: Cell Rep. 2019 Nov 12;29(7):1878–1892.e7. doi: 10.1016/j.celrep.2019.10.018 (PMC6856759; doi:10.1016/j.celrep.2019.10.018)
Supplement: Document S2. Article plus Supplemental Information [file mmc2.pdf]

Cell Reports

Article

Aryl Hydrocarbon Receptor Contributes to the Transcriptional Program of IL-10-Producing Regulatory B Cells

Graphical Abstract

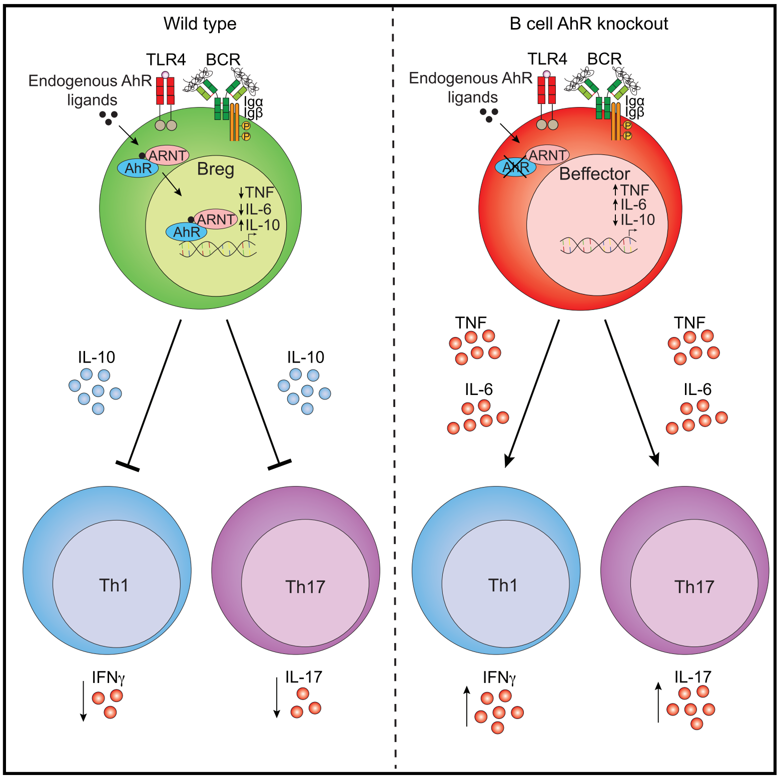

Highlights

- IL-10^{+} Bregs are identified by high expression of AhR
- B cell AhR deficiency leads to exacerbated arthritis and impaired Breg function
- AhR directly binds to and regulates the expression of IL-10 in Bregs
- AhR maintains Breg phenotype by suppressing pro-inflammatory gene expression

Authors

Christopher J.M. Piper, Elizabeth C. Rosser, Kristine Oleinika, ..., Christoph Bock, Brigitta Stockinger, Claudia Mauri

Correspondence

c.mauri@ucl.ac.uk

In Brief

The transcriptional control of interleukin-10 (IL-10) in regulatory B cells (Bregs) remains undefined. Piper et al. identify the aryl hydrocarbon receptor (AhR) as an important transcription factor involved in Breg differentiation and show a direct role of AhR in the regulation of IL-10 transcription.

Piper et al., 2019, Cell Reports *29*, 1878–1892
November 12, 2019 © 2019 The Author(s).
https://doi.org/10.1016/j.celrep.2019.10.018

CellPress

OPEN ACCESS
CellPress

Cell Reports
Article

Aryl Hydrocarbon Receptor Contributes to the Transcriptional Program of IL-10-Producing Regulatory B Cells

Christopher J.M. Piper,[1] Elizabeth C. Rosser,[1,2,3] Kristine Oleinika,[1] Kiran Nistala,[1] Thomas Krausgruber,[4] André F. Rendeiro,[4] Aggelos Banos,[5] Ignat Drozdov,[6] Matteo Villa,[7] Scott Thomson,[1] Georgina Xanthou,[8] Christoph Bock,[4,9,10] Brigitta Stockinger,[7] and Claudia Mauri[1,11,*]
[1]Centre for Rheumatology, Division of Medicine, University College London, London, UK
[2]University College London Great Ormond Street Institute of Child Health, 30 Guilford Street, London, WC1N 1EH, UK
[3]Versus Arthritis Centre for Adolescent Rheumatology at University College London, University College London Hospitals and Great Ormond Street Hospital, London, UK
[4]CeMM Research Center for Molecular Medicine of the Austrian Academy of Sciences, Vienna, Austria
[5]Laboratory of Inflammation and Autoimmunity, Biomedical Research Foundation of the Academy of Athens (BRFAA), Athens, Greece
[6]Bering Limited, London, TW2 5EA, UK
[7]The Francis Crick Institute, London, NW1 1AT, UK
[8]Cellular Immunology Lab, Biomedical Research Foundation of the Academy of Athens (BRFAA), Athens, Greece
[9]Department of Laboratory Medicine, Medical University of Vienna, Vienna, Austria
[10]Max Planck Institute for Informatics, Saarland Informatics Campus, Saarbrücken, Germany
[11]Lead Contact
*Correspondence: c.mauri@ucl.ac.uk
https://doi.org/10.1016/j.celrep.2019.10.018

SUMMARY

Regulatory B cells (Bregs) play a critical role in the control of autoimmunity and inflammation. IL-10 production is the hallmark for the identification of Bregs. However, the molecular determinants that regulate the transcription of IL-10 and control the Breg developmental program remain unknown. Here, we demonstrate that aryl hydrocarbon receptor (AhR) regulates the differentiation and function of IL-10-producing $CD19^{+}CD21^{hi}CD24^{hi}$Bregs and limits their differentiation into B cells that contribute to inflammation. Chromatin profiling and transcriptome analyses show that loss of AhR in B cells reduces expression of IL-10 by skewing the differentiation of $CD19^{+}CD21^{hi}CD24^{hi}$B cells into a pro-inflammatory program, under Breg-inducing conditions. B cell AhR-deficient mice develop exacerbated arthritis, show significant reductions in IL-10-producing Bregs and regulatory T cells, and show an increase in T helper (Th) 1 and Th17 cells compared with B cell AhR-sufficient mice. Thus, we identify AhR as a relevant contributor to the transcriptional regulation of Breg differentiation.

INTRODUCTION

B cells with immunosuppressive capacity, known as regulatory B cells (Bregs), play an important role in restraining inflammation. In mice, regulatory function has been ascribed to IL-10-producing transitional type 2 marginal zone precursors (T2-MZPs) (Evans et al., 2007), marginal zone (MZ) (Gray et al., 2007) and $CD1d^{hi}CD5^{+}$B cells (Yanaba et al., 2008), plasmablast (Matsumoto et al., 2014), and plasma cell (Lino et al., 2018) populations. Bregs suppress inflammatory cytokine production by T cells and promote the differentiation of $Foxp3^{+}$ regulatory T cells (Tregs), primarily via the secretion of interleukin-10 (IL-10) (Carter et al., 2011; Rosser et al., 2014). Mice lacking IL-10-expressing B cells develop exacerbated autoimmune arthritis and experimental autoimmune encephalitis (EAE) (Carter et al., 2011; Fillatreau et al., 2002), and adoptive transfer of IL-10-deficient B cells to arthritic mice fails to suppress inflammation (Carter et al., 2011).

B cell receptor (BCR) engagement, Toll-like receptor (TLR) agonists lipopolysaccharide (LPS; TLR2 and TLR4) (Lampropoulou et al., 2008; Tian et al., 2001), CpG oligo-deoxynucleotides (TLR9), and inflammatory cytokines such as IL-1β and IL-6 or IFN-α are all potent inducers of B cell-derived IL-10, suggesting an important role for Bregs in the restoration of tolerance after infection or inflammation (Menon et al., 2016; Rosser et al., 2014). Cell-derived signals arising from B and T lymphocyte cross-talk, including T cell-derived CD40L and IL-21, further support the expansion of Bregs (Mauri et al., 2000; Yoshizaki et al., 2012). Bregs, in turn, suppress inflammatory cytokine production by T cells and promote the differentiation of $Foxp3^{+}$ regulatory T cells (Treg) (Carter et al., 2011; Oleinika et al., 2018; Rosser et al., 2014).

Unlike in murine T cells, in which it is well established that IL-10 expression is controlled by several transcription factors, including c-Maf and the aryl hydrocarbon receptor (AhR) (Apetoh et al., 2010), there is limited knowledge of the transcriptional control of IL-10 production by Bregs. To date, studies of the molecular control of B cell IL-10 production have been limited to the examination of NFAT downstream of STIM1/STIM2 calcium sensors (Matsumoto et al., 2011) and Myd88 (Liu et al., 2019) in mice and ERK (Li et al., 2012) and STAT3 (Blair et al., 2010) in humans.

 © 2019 The Author(s).
This is an open access article under the CC BY license (http://creativecommons.org/licenses/by/4.0/).

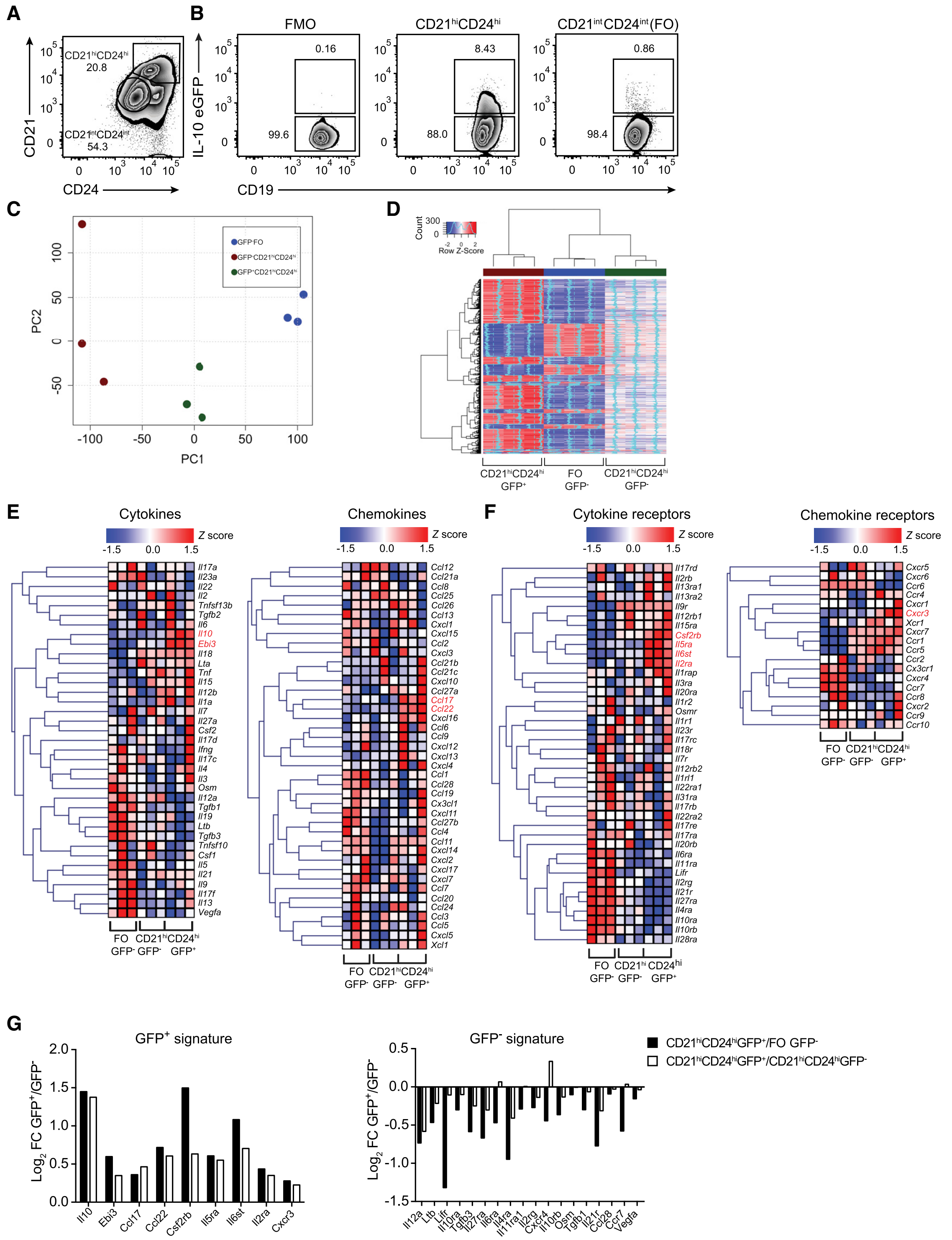

Figure 1. Bregs Express a Highly Specific Cytokine and Chemokine Transcriptional Profile

Antigen-induced arthritis (AIA) was induced in IL-10eGFP reporter (Vert-X) mice.

(A) Representative flow cytometry plots showing the frequency of $CD19^{+}CD21^{hi}CD24^{hi}$ and $CD19^{+}CD21^{int}CD24^{int}$(FO)B cells.

(B) Representative flow cytometry plots showing the frequency of IL-10eGFP^{+} respectively in $CD19^{+}CD21^{hi}CD24^{hi}$ and FO B cell subsets.

(C) Principal-component analysis of transcripts in $CD19^{+}CD21^{hi}CD24^{hi}$eGFP^{+}, $CD19^{+}CD21^{hi}CD24^{hi}$eGFP^{-}, and FO B cell subsets (n = 3).

(D) Heatmap showing the expression of genes by $CD19^{+}CD21^{hi}CD24^{hi}GFP^{+}$, $CD19^{+}CD21^{hi}CD24^{hi}GFP^{-}$, and FO B cells. Blue dashed line represents SD of 0.

(E) Heatmaps showing the expression of cytokine (left) and chemokine (right) genes in the respective subsets.

(legend continued on next page)

More recently, Blimp1 and IRF4 (Matsumoto et al., 2014) have been linked to IL-10^{+} plasmablasts, but at least for Blimp1 and IRF4, neither transcription factor appeared to directly control the production of IL-10 by splenic B cells (Matsumoto et al., 2014).

To explore the molecular mechanisms regulating the differentiation of B cells into IL-10-producing Bregs, we used an IL-10-eGFP reporter mouse (Madan et al., 2009). We compared the gene expression profiles of IL-10eGFP^{+}CD19^{+}CD21^{hi}CD24^{hi} Bregs, IL-10eGFP^{-}CD19^{+}CD21^{hi}CD24^{hi}B cells, and IL-10eGFP^{-}CD19^{+}CD21^{int}CD24^{int} follicular (FO) B cells isolated from arthritic mice (FO B cells do not produce IL-10 and do not suppress arthritis after adoptive transfer; Evans et al., 2007). We chose to study IL-10^{+}CD19^{+}CD21^{hi}CD24^{hi}Bregs because this population contains both T2-MZP and MZ B cells, which together have been shown to contain the vast majority of splenic IL-10-producing Bregs (Evans et al., 2007; Gray et al., 2007).

IL-10eGFP^{+}CD19^{+}CD21^{hi}CD24^{hi}Bregs have a unique transcriptome, characterized by a highly restricted cytokine/chemokine profile that distinguishes them from the IL-10eGFP^{-}B cell subsets. AhR was among the most significant differentially expressed transcription factors in IL-10eGFP^{+}CD19^{+}CD21-^{hi}CD24^{hi}Bregs, compared with both IL-10eGFP^{-}CD19^{+}CD21-^{hi}CD24^{hi}B cells and IL-10eGFP^{-}FO B cells. We show that AhR binds upstream to the transcription start site (TSS) of the *Il10* locus in IL-10eGFP^{+}B cells but not in IL-10eGFP^{-}B cells. LPS and anti-IgM, stimuli previously shown and confirmed here to induce the expression of AhR (Vaidyanathan et al., 2017; Villa et al., 2017), promote the differentiation of CD19^{+}CD21^{hi}CD24^{hi}B cells (a population poised to become Bregs; Evans et al., 2007; Matsumoto et al., 2011) into IL-10^{+}CD19^{+}CD21^{hi}CD24^{hi}Bregs. Taking advantage of high-throughput sequencing, we demonstrated that activation of AhR under this Breg-polarizing condition results in the suppression of several pro-inflammatory cytokine and chemokine genes and in a highly restricted phenotype in CD19^{+}CD21^{hi}CD24^{hi}B cells congruous with an immunosuppressive population.

In vivo, B cell-specific deletion of AhR caused exacerbated arthritis, reduced IL-10 production by CD19^{+}CD21^{hi}CD24^{hi} Bregs, and reduced the frequency of Foxp3^{+}Tregs and expansion of Th1 and Th17 cells. These results support a role of AhR in the differentiation of IL-10-producing Bregs and in the control of their immunosuppressive phenotype.

RESULTS

IL-10^{+} Bregs Present a Restricted Cytokine and Chemokine Gene Expression Profile

To identify candidate genes that regulate the transcription of IL-10 in Bregs, arthritis was induced in IL-10eGFP reporter mice (Vert-x) (Madan et al., 2009). We sorted splenic IL-10eGFP^{+}CD19^{+}CD21^{hi}CD24^{hi}Bregs, IL-10eGFP^{-}CD19^{+}CD21^{hi} CD24^{hi}B cells, and IL-10eGFP^{-}FO B cells (the two GFP^{-} populations are hereafter referred to as IL-10eGFP^{-}B cell subsets) and profiled these cells using gene expression microarray (Figures 1A and 1B). Of note, very few IL-10-producing Bregs were present in the joint or draining lymph nodes (DLNs) of arthritic mice (Figure S1A). This sorting strategy was chosen to capture the majority of described Breg subsets, including IL-10^{+}T2-MZP, IL-10^{+}MZ, and IL-10^{+}CD1d^{hi}CD5^{+}, which have been shown to exert suppressive capacity via IL-10 in this model of arthritis and in other models of autoimmunity (Brummel and Lenert, 2005; Evans et al., 2007; Tian et al., 2001; Yanaba et al., 2009). Virtually no IL-10-expressing plasma cells or plasmablasts (LAG-3^{+} plasma cells [Lino et al., 2018] and CD138^{+}CD44^{+} plasmablasts [Matsumoto et al., 2014]) were detected in the spleen or DLNs following the induction of arthritis (Figures S1B–S1H). The purity of sorted IL-10eGFP^{+} and IL-10eGFP^{-} subsets isolated for the microarray was more than 98% (Figure S1I). Principal-component analysis (PCA) revealed three distinct groups along the first dimension, with the IL-10eGFP^{+}CD19^{+}CD21^{hi}CD24^{hi}Breg population clustered separately away from both IL-10eGFP^{-}B cell subsets (Figure 1C). Analysis of gene expression revealed 1,073 differentially expressed genes (DEGs) between IL-10eGFP^{+}CD19^{+}CD21^{hi} CD24^{hi}Bregs and IL-10eGFP^{-}CD19^{+}CD21^{hi}CD24^{hi}B cells and 1,267 genes that were differentially expressed between IL-10eGFP^{+}CD19^{+}CD21^{hi}CD24^{hi}Bregs and IL-10eGFP^{-}FO B cells (fold change > 1.5 and adjusted p value < 0.05) (Figure S1J; Figure 1D).

In the context of arthritis, splenic Bregs have been shown to produce mainly IL-10 (Rosser and Mauri, 2015). Of the cytokine genes upregulated in IL-10eGFP^{+}CD19^{+}CD21^{hi}CD24^{hi}Bregs, only *Il10* and *Ebi3* reached an adjusted p value of < 0.05, compared with both IL-10eGFP^{-} B cell subsets. However, because *Il12a* was not found to be upregulated in IL-10eGFP^{+}CD19^{+}CD21^{hi}CD24^{hi}Bregs, we excluded a role of IL-35 in these cells. Although a trend in the increase of transcripts for pro-inflammatory genes such as *Il1a*, *Il12b*, *Il15*, and *Il18* was noted, the expression of these genes was not significantly different from the IL-10eGFP^{-}B cell subsets (Figure 1E). IL-10eGFP^{-}FO B cells, which unlike CD19^{+}CD21^{hi}CD24^{hi}B cells do not suppress inflammation on adoptive transfer (Evans et al., 2007), express a different transcriptional profile, characterized by higher expression of cytokines and cytokine receptors known to mediate inflammatory responses, compared with IL-10eGFP^{+}CD19^{+}CD21^{hi}CD24^{hi}Bregs (Figures 1E and 1F). The transcripts that were significantly differentially expressed in IL-10eGFP^{+}CD19^{+}CD21^{hi}CD24^{hi}Bregs versus IL-10eGFP^{-}B cell subsets are summarized in (Figure 1G). In keeping with the anti-inflammatory role of Bregs, we found that the Th2-attracting

(F) Heatmaps of cytokine receptor (left) and chemokine receptor (right) expression profiles in the respective subsets (n = 3).
(G) Log_2 fold changes (FCs) of all significant genes identified in (E) and (F) for the GFP^{+} (left graph) and GFP^{-} (right graph) signatures. Log_2 FCs are highlighted for GFP^{+} versus both GFP^{-} populations.
All experiments were performed at day 7 post-IA injection. For (D)–(F), heatmaps show *Z* scores based on normalized GeneChip robust multiarray averaging (GC-RMA) values. Listed genes highlighted in red are upregulated in the CD19^{+}CD21^{hi}CD24^{hi}eGFP^{+} population compared with CD19^{+}CD21^{hi}CD24^{hi}eGFP^{-} (adjusted p value < 0.05). In (A) and (B), data are representative of at least five independent experiments.
See also Figure S1.

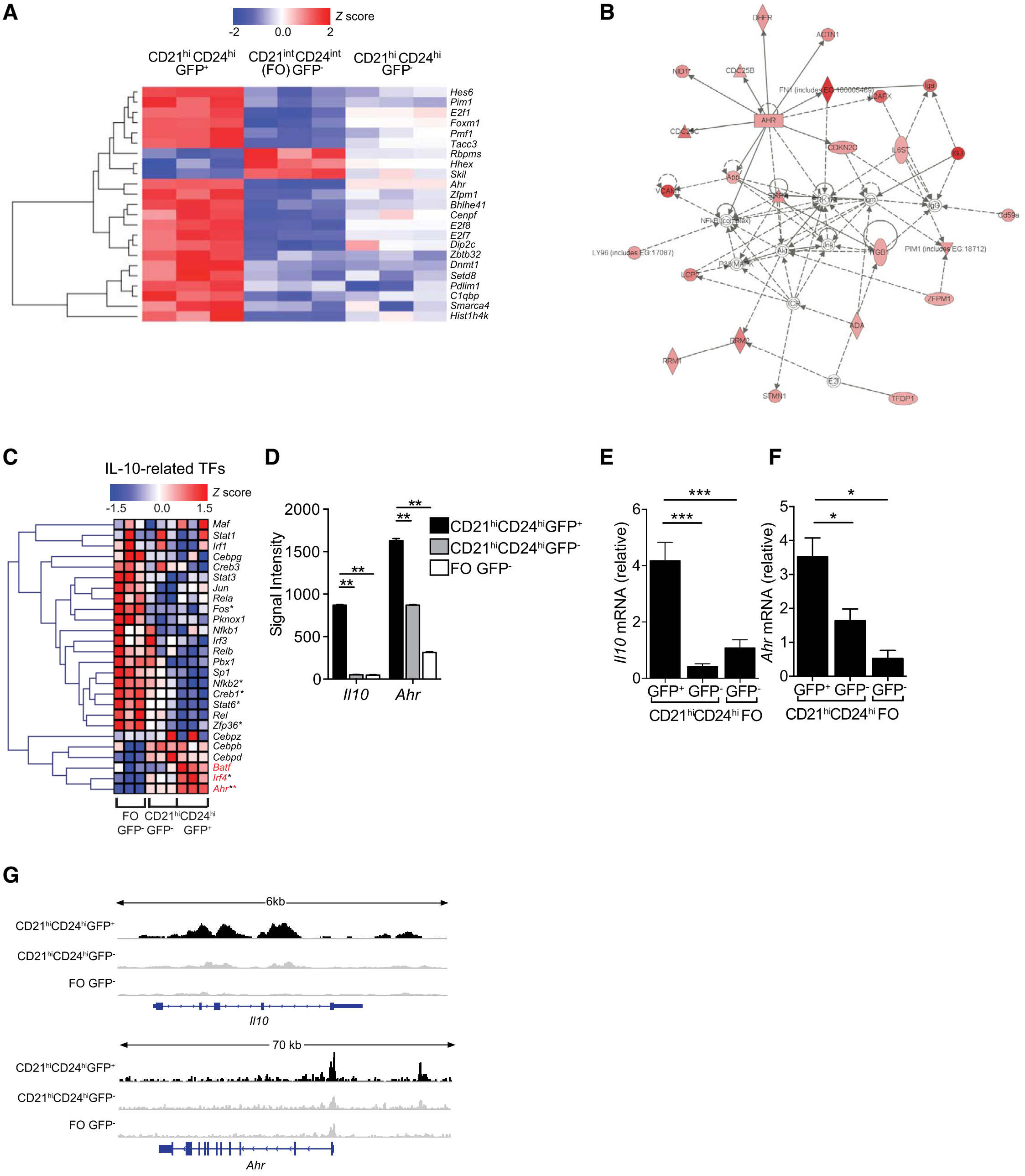

Figure 2. Identification of AhR as a Key IL-10 Associated Transcription Factor in Bregs

Antigen-induced arthritis (AIA) was induced in IL-10eGFP reporter mice.

(A) Heatmap showing *Z* scores of significant DEG (n = 23, adjusted p value < 0.05) based on normalized GC-RMA values, filtered on transcription factor activity Gene Ontology terms in sorted $CD19^{+}CD21^{hi}CD24^{hi}eGFP^{+}$, $CD19^{+}CD21^{hi}CD24^{hi}eGFP^{-}$, and FO B cells.

(B) Ingenuity pathway network analysis identifies a cluster of genes with AhR as the central hub. The lines between genes represent known interactions (solid lines represent direct interactions, dashed lines represent indirect interactions).

(C) Heatmap of *Z* scores of transcription factors regulating IL-10, expressed by $CD19^{+}CD21^{hi}CD24^{hi}eGFP^{+}$, $CD19^{+}CD21^{hi}CD24^{hi}eGFP^{-}$, and FO B cells. Genes highlighted in red are upregulated in the $CD19^{+}CD21^{hi}CD24^{hi}GFP^{+}$ population compared with $eGFP^{-}$ populations. Black asterisks, adjusted p value < 0.05 for the comparison of $CD19^{+}CD21^{hi}CD24^{hi}eGFP^{+}$ versus FO $eGFP^{-}$. Red asterisks, adjusted p value < 0.05 for the comparison of $CD19^{+}CD21^{hi}CD24^{hi}eGFP^{+}$ versus $CD19^{+}CD21^{hi}CD24^{hi}eGFP^{-}$.

(D) Microarray signal intensities of *Il10* and *Ahr* (n = 3).

(E and F) Validation of (E) *Il10* and (F) *Ahr* mRNA expression in the indicated B cell subsets by qPCR (n = 3).

(legend continued on next page)

OPEN
ACCESS
CellPress

chemokines *Ccl17* and *Ccl22* (Imai et al., 1999; Nakayama et al., 2004), and the chemokine receptor *Cxcr3*, important for the trafficking of lymphocytes to the synovium in arthritis (Mohan and Issekutz, 2007), were upregulated in IL-10eGFP$^+$CD19$^+$CD21^{hi}CD24^{hi}Bregs compared with IL-10eGFP$^-$B cell subsets (Figures 1E–1G). These results showed that in the context of arthritis, splenic Bregs displayed a distinct anti-inflammatory transcriptional profile compared with IL-10eGFP$^-$B cell subsets.

AhR Is Highly Expressed in IL-10-Producing Bregs

To screen for molecules involved in IL-10 transcription and Breg function, genes concordant for comparison 1 (between IL-10eGFP$^+$CD19$^+$CD21^{hi}CD24^{hi}Bregs and IL-10eGFP$^-$FO B cells) and comparison 2 (between IL-10eGFP$^+$CD19$^+$CD21^{hi}CD24^{hi}Bregs and IL-10eGFP$^-$CD19$^+$CD21^{hi}CD24^{hi}B cells) (Figure S1K) were filtered on the basis of the transcription factor Gene Ontology term, resulting in 23 candidates (Figure 2A). Pathway analyses showed that AhR represented a central network hub (Figure 2B) and was the most significantly enriched candidate in IL-10eGFP$^+$CD19$^+$CD21^{hi}CD24^{hi}Bregs (adjusted p value < 3.34×10^{-5}; Table S1; Figure S1L). Analysis of transcription factors previously shown to be associated with IL-10 transcriptional regulation in other lymphocyte subsets, including Tregs (Iyer and Cheng, 2012), confirmed that AhR was the most significantly upregulated IL-10-related transcription factors in IL-10eGFP$^+$CD19$^+$CD21^{hi}CD24^{hi}Bregs, in comparison with IL-10eGFP$^-$B cell subsets (Figure 2C). Microarray signal intensities for *Il10* and *Ahr* were higher in IL-10eGFP$^+$CD19$^+$CD21^{hi}CD24^{hi}Bregs compared with IL-10eGFP$^-$B cell subsets (Figure 2D). qPCR analysis confirmed that *Il10* and *Ahr* mRNA expression was higher in IL-10eGFP$^+$CD19$^+$CD21^{hi}CD24^{hi}Bregs than in both IL-10eGFP$^-$B cell subsets (Figures 2E and 2F). Corroborating the results in Figures 2E and 2F, assay for transposase-accessible chromatin using sequencing (ATAC-seq) showed increased accessibility in both the *Il10* and *Ahr* loci in IL-10eGFP$^+$CD19$^+$CD21^{hi}CD24^{hi}Bregs, in comparison with both IL-10eGFP$^-$B cell subsets (Figure 2G).

AhR Upregulation Promotes the Generation of IL-10$^+$CD19$^+$CD21^{hi}CD24^{hi}Bregs

We and others have recently shown that BCR signals combined with TLR stimulation induce a substantial upregulation of AhR in B cells (Vaidyanathan et al., 2017; Villa et al., 2017). Because BCR signals together with TLR engagement are known to be pivotal in the generation of Bregs (Rosser and Mauri, 2015), next we investigated whether the differentiation of Bregs "marked" by the induction of IL-10 expression by these stimuli was AhR dependent. For this purpose, CD19$^+$CD21^{hi}CD24^{hi}B cells were sorted from the spleens of arthritic mice and stimulated with LPS ± anti-IgM. An 11-fold increase in the frequency of IL-10$^+$CD19$^+$CD21^{hi}CD24^{hi}Bregs and a 200-fold increase in the production of IL-10 by CD19$^+$CD21^{hi}CD24^{hi}Bregs were observed upon LPS+anti-IgM stimulation compared with unstimulated cells (Figures 3A–3C). Increased levels of AhR expression in IL-10$^+$CD19$^+$CD21^{hi}CD24^{hi} Bregs compared with IL-10$^-$FO and IL-10$^-$CD19$^+$CD21^{hi}CD24^{hi}B cells were confirmed using flow cytometry (Figures S2A and S2B). We established the kinetics of AhR and AhR pathway-associated gene expression in relation to *Il10* transcription, after activation with Breg-polarizing stimuli. We observed a peak in the expression of *Ahr* and *Cyp1a1* (the gene encoding the AhR-dependent cytochrome P4501A1) at 6 h post-stimulation with LPS+anti-IgM, followed by an upregulation of AhR repressor (*Ahrr*) and *Il10* at 24 h. No significant changes in the expression of the AhR binding partner AhR nuclear translocator (*Arnt*) were observed (Figures 3D–3H). Of note, *ex vivo* CD19$^+$CD21^{hi}CD24^{hi}B cells display higher expression of *Ahr* (confirmed at the protein level using flow cytometry and western blotting), *Il10, Cyp1a1*, *Ahrr*, and *Arnt* compared with FO B cells (Figures S2C–S2J). Together these data show that IL-10$^+$CD19$^+$CD21^{hi}CD24^{hi}Bregs express the highest levels of AhR compared with the IL-10$^-$CD19$^+$CD21^{hi}CD24^{hi} and IL-10$^-$FO B cells and that in CD19$^+$CD21^{hi}CD24^{hi}B cells, AhR upregulation precedes the production of IL-10.

To understand the role of AhR in the regulation and function of IL-10 expression in CD19$^+$CD21^{hi}CD24^{hi}Bregs, we isolated CD19$^+$CD21^{hi}CD24^{hi}B cells and FO B cells from *Ahr*$^{+/-}$ and *Ahr*$^{-/-}$ mice and stimulated them *in vitro* with LPS+anti-IgM. Lack of AhR significantly reduced the ability of CD19$^+$CD21^{hi}CD24^{hi}B cells to differentiate into IL-10-producing CD19$^+$CD21^{hi}CD24^{hi}Bregs compared with AhR competent CD19$^+$CD21^{hi}CD24^{hi}B cells (Figure 3I). In addition, *Ahr*$^{-/-}$ CD19$^+$CD21^{hi}CD24^{hi}B cells presented a reduced capacity to inhibit IFN-γ production by CD4$^+$T cells *in vitro* compared with *Ahr*$^{+/-}$ CD19$^+$CD21^{hi}CD24^{hi}B cells (Figures 3J and 3K). FO B cells failed to produce IL-10 and to suppress IFN-γ by CD4$^+$ T cells, irrespective of AhR expression (Figures 3I–3K).

To assess if activation of AhR directly with endogenous ligands promotes the differentiation of CD19$^+$CD21^{hi}CD24^{hi}B cells into IL-10$^+$CD19$^+$CD21^{hi}CD24^{hi}Bregs, we stimulated sorted CD19$^+$CD21^{hi}CD24^{hi}B cells or FO B cells with the AhR ligand 6-formylindolo(3,2-b) carbazole (FICZ). AhR activation significantly upregulated the expression of *Cyp1a1* and *Il10* in the CD19$^+$CD21^{hi}CD24^{hi}B cell subset but not in FO B cells compared with the vehicle control (Figure 3L). Secretion of IL-10 was further enhanced by the addition of FICZ to LPS+anti-IgM-stimulated CD19$^+$CD21^{hi}CD24^{hi}B cells, compared with LPS+anti-IgM alone (Figure S2K). An increase in Breg IL-10 expression was observed when CD19$^+$CD21^{hi}CD24^{hi}B cells were cultured in Iscove's modified Dulbecco's medium (IMDM; enriched in aromatic amino acids that give rise to AhR ligands; Veldhoen et al., 2009) (Heath-Pagliuso et al., 1998) compared with RPMI media (Figure S2L).

We next investigated whether AhR regulates IL-10 expression in IL-10eGFP$^+$CD19$^+$CD21^{hi}CD24^{hi}Bregs by directly binding the

(G) Representative ATAC-seq tracks for the *Il10* and *Ahr* loci in CD19$^+$CD21^{hi}CD24^{hi}eGFP$^+$, CD19$^+$CD21^{hi}CD24^{hi}eGFP$^-$, and FO B cells (n = 3). Track heights between samples are normalized through group autoscaling. For qPCR, gene expression was calculated normalizing to β-actin.
All experiments were performed at day 7 post-IA injection. In (D)–(F), data are expressed as mean ± SEM. In (E) and (F), data are representative of three independent experiments with biological replicates. *p < 0.05, **p < 0.01, and ***p < 0.001, one- and two-way ANOVA.
See also Figures S1 and S2 and Table S1.

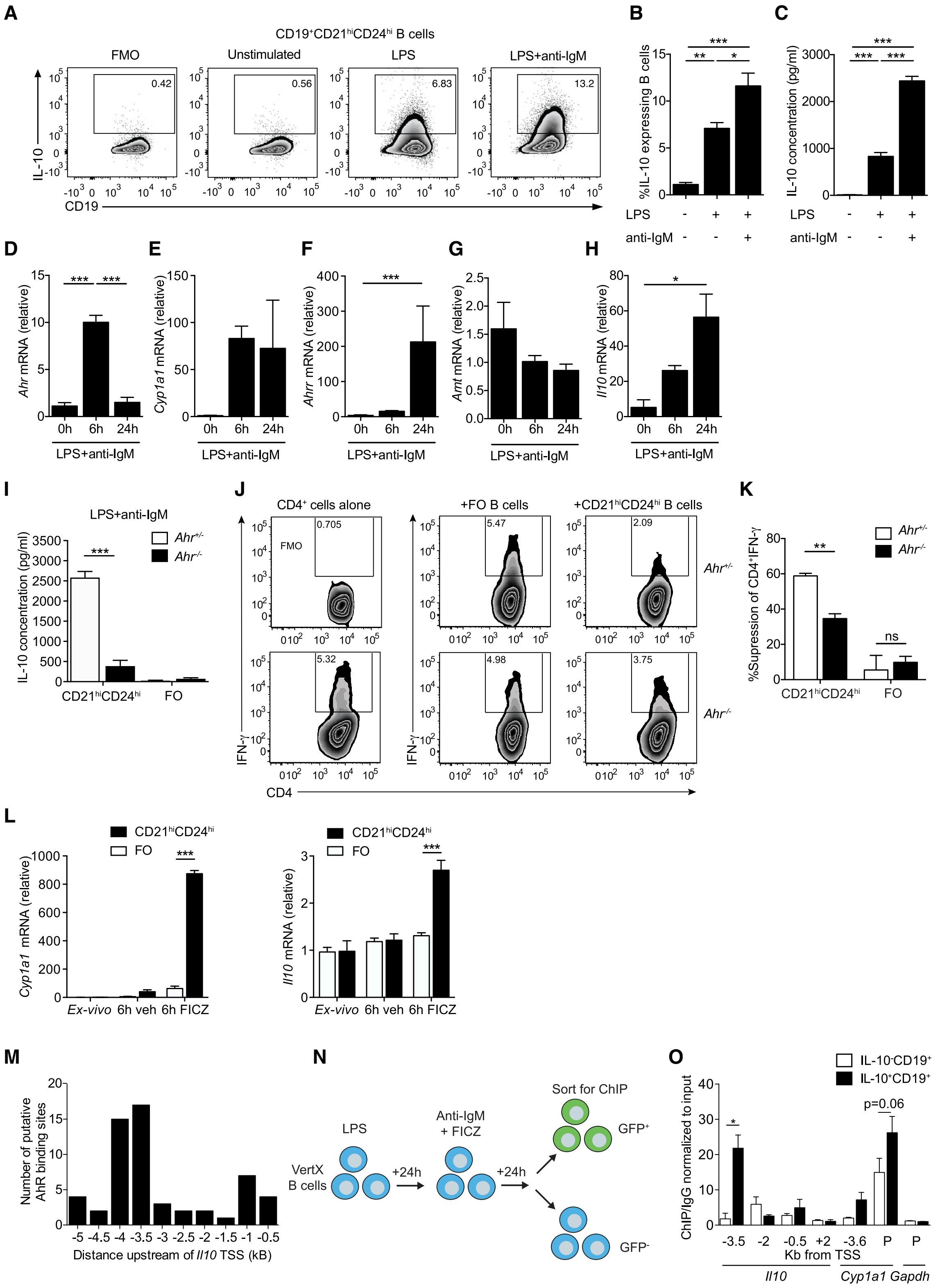

(legend on next page)

Il10 locus. To address this, we took advantage of the JASPAR tool (Khan et al., 2018) and identified putative AhR binding sites in 500 bp regions up to −5 kb upstream and +5 kb downstream of the *Il10* TSS and designed primer probes to span these regions (Figure 3M). We sorted IL-10eGFP$^+$ and IL-10eGFP$^-$B cells, after stimulation with LPS+anti-IgM+FICZ (the combination of stimuli was used to maximize AhR activation and translocation to the nucleus) and performed chromatin immunoprecipitation (ChIP) qPCR on AhR-bound DNA (Figure 3N). Significantly enriched AhR binding was observed at −3.5 kb upstream of the *Il10* TSS in the IL-10eGFP$^+$ population. Minimal binding of AhR was observed in other regions of the *Il10* locus. As a positive control, we confirmed that under these experimental conditions, there was enriched binding of AhR to the promoter of *Cyp1a1* but no binding to *Gapdh,* an AhR-independent housekeeping gene (Figure 3O). These results show that AhR controls IL-10 transcription and the function of Bregs.

AhR Controls the Breg Transcriptional Program by Suppressing Pro-inflammatory Gene Expression

To examine the role of AhR in controlling the differentiation of CD19$^+$CD21^{hi}CD24^{hi}B cells into Bregs, and to ascertain the relative contribution of AhR in establishing the restricted Breg phenotype identified in the microarray (Figures 1E–1G) in addition to IL-10 production, we took advantage of mice with a B cell-specific deficiency of AhR (*Ahr*$^{fl/-}$*Mb1*$^{cre/+}$) (Villa et al., 2017)) (Figures S3A and S3B). The use of these mice avoids any cell extrinsic effects of AhR that could indirectly influence Breg differentiation (Stockinger et al., 2014). CD19$^+$CD21^{hi}CD24^{hi}B cells were sorted from immunized *Mb1*$^{cre/+}$ and *Ahr*$^{fl/-}$*Mb1*$^{cre/+}$ mice, and the transcriptional profile of sorted CD19$^+$CD21^{hi}CD24^{hi}B cells was compared before and after stimulation under Breg-polarizing conditions (LPS+anti-IgM). Both the normalized counts for *Ahr* and the accessibility of the *Ahr* locus measured by ATAC-seq increased in LPS+anti-IgM-stimulated *Mb1*$^{cre/+}$ CD19$^+$CD21^{hi}CD24^{hi}B cells compared with *ex vivo Mb1*$^{cre/+}$ CD19$^+$CD21^{hi}CD24^{hi}B cells (Figures S4A and S4B). Signaling pathway impact analysis (SPIA) of DEGs revealed overrepresented pathways relating to cytokine-cytokine receptor interactions and chemokine signaling in stimulated *Mb1*$^{cre/+}$ CD19$^+$CD21^{hi}CD24^{hi}B cells versus *ex vivo Mb1*$^{cre/+}$ CD19$^+$CD21^{hi}CD24^{hi}B cells (Figure 4A). Analysis of the genes differentially expressed within this pathway confirmed that under Breg-polarizing conditions, several genes identified in the IL-10eGFP$^+$ signature (as shown in Figure 1G), including *Il10*, *Ccl22*, and *Il2ra*, were upregulated, while those associated with the IL-10eGFP$^-$ signature, including *Il12a*, *Il10ra*, and *Ltb*, were downregulated under Breg-polarizing conditions (Figure 4B; Figure S4C).

A signature of genes related to cytokine-cytokine receptor interaction was upregulated in *Ahr*$^{fl/-}$*Mb1*$^{cre/+}$ CD19$^+$CD21^{hi}CD24^{hi} B cells under LPS+anti-IgM stimulation compared with *Mb1*$^{cre/+}$ (Figure 4C). Forty-four of 102 genes in this pathway were significantly differentially expressed between *Ahr*$^{fl/-}$*Mb1*$^{cre/+}$ and *Mb1*$^{cre/+}$ CD19$^+$CD21^{hi}CD24^{hi}B cells (Figures 4C and 4D). Of the genes that were differentially expressed under Breg-polarizing conditions (Figure 4B), pro-inflammatory cytokines including *Il6*, *Tnf*, *Il2*, and chemokines such as *Ccl3*, *Ccl5*, and *Cxcl16*, known to recruit lymphocytes to the inflamed synovia in models of arthritis (Kasama et al., 1995; Ruth et al., 2006; Szekanecz et al., 2000; Thornton et al., 1999), were upregulated in the absence of AhR (Figure 4D). The absence of AhR expression led to downregulation of the *Il5ra* gene, which had previously been associated with Breg function (Klinker et al., 2013), *Ccl22*, and *Il18*, which we have identified as Breg-associated genes in the microarray. Testing key arthritogenic pro-inflammatory transcripts (Feige et al., 2000; Ohshima et al., 1998; Thornton et al., 2000) using qPCR confirmed that *Il2*, *Il6*, and *Tnf* were increased in *Ahr*$^{fl/-}$*Mb1*$^{cre/+}$ CD19$^+$CD21^{hi}CD24^{hi}B cells, and *Ccl22* and *Il5ra* were decreased compared with *Mb1*$^{cre/+}$ CD19$^+$CD21^{hi}CD24^{hi}B cells. The increase in pro-inflammatory

Figure 3. The Effect of AhR in the Differentiation of CD19$^+$CD21^{hi}CD24^{hi} into Bregs

Antigen-induced arthritis (AIA) was induced in IL-10eGFP reporter or C57BL/6 mice.

(A and B) Representative flow cytometry plots (A) and bar chart (B) showing the percentage of IL-10 expression in CD19$^+$CD21^{hi}CD24^{hi}B cells (n = 5). In these experiments, CD19$^+$CD21^{hi}CD24^{hi}B cells were stimulated for 24 h with LPS, followed by an additional 24 h with anti-IgM.

(C) IL-10 production, as measured by ELISA (n = 7).

(D–H) CD19$^+$CD21^{hi}CD24^{hi}B cells were isolated from WT C57BL/6 mice and stimulated for 6 or 24 h with LPS+anti-IgM. The mRNA levels of (D) *Ahr*, (E) *Cyp1a1*, (F) *Ahrr*, (G) *Arnt*, and (H) *Il10* were analyzed *ex vivo* and after stimulation (n = 3).

(I) IL-10 production measured by ELISA in LPS+anti-IgM-stimulated CD19$^+$CD21^{hi}CD24^{hi}B cells and FO B cells (n = 4 per group).

(J) CpGb-stimulated CD19$^+$CD21^{hi}CD24^{hi} and FO B cells from *Ahr*$^{+/-}$ and *Ahr*$^{-/-}$ mice co-cultured for 72 h with anti-CD3 stimulated autologous CD4$^+$T cells from *Ahr*$^{+/-}$ mice. Representative flow cytometry plots showing the frequency of IFN-γ^+CD4$^+$ T cell.

(K) Bar chart showing percentage suppression of IFN-γ^+CD4$^+$ T cells by splenic CD19$^+$CD21^{hi}CD24^{hi}B cells or FO B cells following stimulation with anti-CD3 (n = 3).

(L) CD19$^+$CD21^{hi}CD24^{hi} and FO B cells were stimulated for 6 h with either vehicle alone (DMSO) or the AhR agonist FICZ, and the expression of *Cyp1a1* and *Il10* was measured using qRT-PCR (n = 3).

(M) Jaspar binding motif analysis of putative AhR binding sites (XRE sites) in 500 bp regions of DNA, upstream of the *Il10* TSS.

(N) Schematic representing the experimental design for the ChIP-qPCR assay. Briefly, we isolated splenic B cells from IL-10eGFP reporter mice and stimulated them for 24 h with LPS, followed by 24 h with anti-IgM+FICZ.

(O) ChIP analysis of AhR binding to the *Il10* locus was performed in IL-10eGFP$^+$CD19$^+$ and IL-10eGFP$^-$CD19$^+$ B cells. Bar chart showing the relative enrichment of AhR binding to regions upstream/downstream or in the promoters (labeled as P) of *Il10*, *Cyp1a1*, and *Gapdh* (n = 3). For qPCR, gene expression was calculated normalizing to β-actin.

All experiments were performed at day 7-post-IA injection. Data representative of at least three independent experiments with biological replicates. In (B)–(I), (K), (L), and (O), data are expressed as mean ± SEM. *p < 0.05, **p < 0.01, and ***p < 0.001, one- and two-way ANOVA.

See also Figure S2.

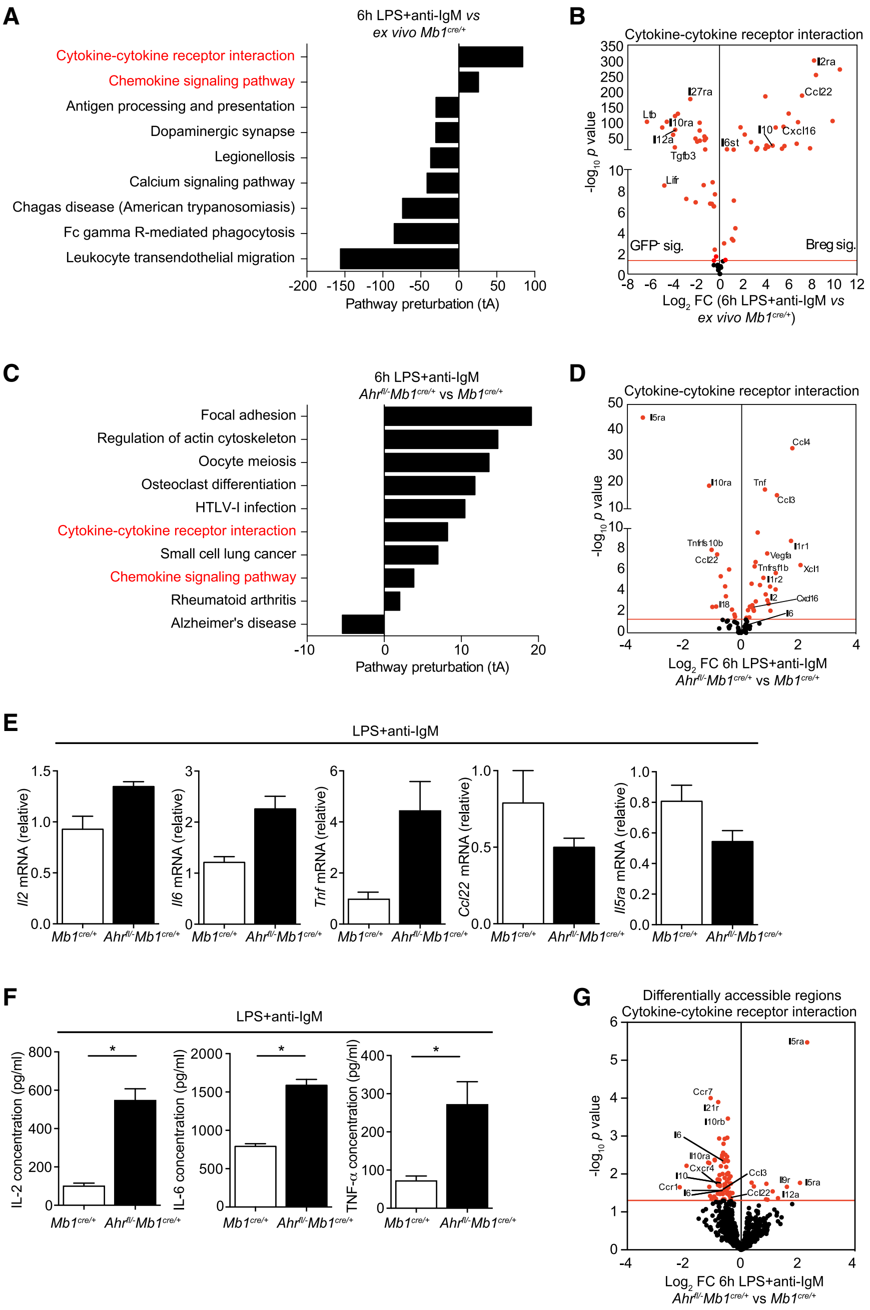

Figure 4. AhR Suppresses Pro-inflammatory Gene Expression during the Differentiation of Bregs

AIA was induced in *Mb1*$^{cre/+}$ and *Ahr*$^{fl/-}$*Mb1*$^{cre/+}$ mice.

(A) Signaling pathway impact analysis (SPIA) showing the top significant ($p < 0.05$) over-represented and under-represented pathways in LPS+anti-IgM-stimulated compared with *ex vivo* CD19^{+}CD21^{hi}CD24^{hi}B cells from *Mb1*$^{cre/+}$ mice. The total perturbation accumulation of these pathways (tA) score is represented on the x axis.

(B) Volcano plot of RNA-seq analysis showing $\log_2$ fold changes (FCs) between LPS+anti-IgM-stimulated compared with *ex vivo* CD19^{+}CD21^{hi}CD24^{hi}B cells from *Mb1*$^{cre/+}$ mice, plotted against $-\log_{10}$ p value for the cytokine-cytokine receptor interaction pathway. Red dots represent significant DEG, with the red line denoting a cut-off p value of < 0.05.

(C) Signaling pathway impact analysis (SPIA) showing the top significant ($p < 0.05$) over-represented and under-represented pathways in 6 h LPS+anti-IgM-stimulated CD19^{+}CD21^{hi}CD24^{hi}B cells from *Ahr*$^{fl/-}$*Mb1*$^{cre/+}$ mice compared with *Mb1*$^{cre/+}$ mice.

(D) Volcano plot of RNA-seq analysis showing $\log_2$ fold changes (FCs) between LPS+anti-IgM-stimulated CD19^{+}CD21^{hi}CD24^{hi}B cells from *Ahr*$^{fl/-}$*Mb1*$^{cre/+}$ versus *Mb1*$^{cre/+}$ mice, plotted against $-\log_{10}$ p value for the cytokine-cytokine receptor interaction pathway.

(E) CD19^{+}CD21^{hi}CD24^{hi}B cells were isolated from *Mb1*$^{cre/+}$ mice and *Ahr*$^{fl/-}$*Mb1*$^{cre/+}$ mice, stimulated for 6 h with LPS+anti-IgM, and assessed for mRNA levels of *Il2*, *Il6*, *Tnf*, *Ccl22*, and *Il5ra* (n = 4).

(F) IL-2, IL-6, and TNFα concentrations from 48 h LPS+anti-IgM-stimulated CD19^{+}CD21^{hi}CD24^{hi} B cells from *Mb1*$^{cre/+}$ mice and *Ahr*$^{fl/-}$*Mb1*$^{cre/+}$ mice (n = 4).

(G) Volcano plot of ATAC-seq DARs in genes taken from the cytokine-cytokine receptor interaction pathway, comparing chromatin accessibility at these sites between *Ahr*$^{fl/-}$*Mb1*$^{cre/+}$ and *Mb1*^{cre} CD19^{+}CD21^{hi}CD24^{hi} B cells after 6 h LPS+anti-IgM. For qPCR, gene expression was calculated normalizing to β-actin.

All experiments were performed at day 7 post-IA injection. For RNA-seq data, n = 3 per condition and genotype. For ATAC-seq data, n = 3 for *Mb1*$^{cre/+}$ mice and n = 2 for *Ahr*$^{fl/-}$*Mb1*$^{cre/+}$ mice. For (E) and (F), data are representative of two independent experiments with biological replicates, and data are expressed as mean ± SEM. *$p < 0.05$, Mann-Whitney test.

See also Figure S4.

cytokines in *Ahr*$^{fl/-}$*Mb1*$^{cre/+}$ CD19^{+}CD21^{hi}CD24^{hi}B cells was confirmed using ELISA (Figures 4E and 4F).

Specific interrogation of differentially accessible regions (DARs) in loci encoding genes from the cytokine-cytokine receptor interaction pathway revealed an overall decrease in chromatin accessibility in these genes in *Ahr*$^{fl/-}$*Mb1*$^{cre/+}$ CD19^{+} CD21^{hi}CD24^{hi}B cells. Seventy-eight DARs ($p < 0.05$) were identified among the genes regulated by AhR at the transcriptional level, including *Il2*, *Il6*, *Ccl3*, *Ccl5*, *Il5ra*, *Ccl22*, and *Il10* between LPS+anti-IgM polarized *Ahr*$^{fl/-}$*Mb1*$^{cre/+}$ and *Mb1*$^{cre/+}$ CD19^{+}CD21^{hi}CD24^{hi}B cells (Figure 4G).

To confirm that AhR suppresses pro-inflammatory gene expression during the development of Bregs, we blocked AhR signaling *in vitro* with CH-223191. Concordant with the results identified with RNA sequencing (RNA-seq), blocking AhR signaling resulted in upregulation of *Il6*

and *Tnf* and downregulation of *Il10* and *Ccl22* mRNA (Figure S4D).

AhR can repress gene expression indirectly by controlling the activity of additional molecules involved in gene regulation, including genes related to the NF-κB pathway. Because we have previously shown that the NF-κB pathway is linked to IL-10 production in Bregs (Rosser et al., 2014), next we analyzed the effect that the lack of AhR in B cells had on the NF-κB pathway in LPS+anti-IgM-stimulated cells. A reduction in NF-κB pathway-related genes, including *Cd40* and *Myd88*, required for IL-10 production, was observed in $Ahr^{fl/-}Mb1^{cre/+}$ $CD19^{+}CD21^{hi}CD24^{hi}$B cells. This was mirrored by upregulation of genes relating to inflammation (*Tnf*, *Tnfaip3*, *Ccl4*, and *Il1r1*) in $Ahr^{fl/-}Mb1^{cre/+}$ compared with $Mb1^{cre/+}$ $CD19^{+}CD21^{hi}$ $CD24^{hi}$B cells (Figure S4E).

Our data suggest that AhR contributes the Breg transcriptional program by suppressing pro-inflammatory gene expression. To rule out whether this effect is secondary to the decrease in IL-10, we cultured wild-type (WT) and $Il10r^{-/-}$ $CD19^{+}CD21^{hi}CD24^{hi}$B cells with LPS+anti-IgM in the presence or absence of the selective AhR antagonist CH-223191. The expression of *Il6* and *Tnf* was significantly increased in both WT and $IL\text{-}10R^{-/-}$ $CD19^{+}CD21^{hi}CD24^{hi}$B cells cultured with the AhR antagonist, suggesting a direct effect of AhR in the suppression of pro-inflammatory gene expression (Figure S4F). Collectively, these data show that under Breg-polarizing conditions, AhR acts as a molecular switch that "turns off" a number of pro-inflammatory cytokines and chemokines in $CD19^{+}CD21^{hi}CD24^{hi}$B cells, while promoting the expression of $IL\text{-}10^{+}CD19^{+}CD21^{hi}CD24^{hi}$Breg-associated cytokines and receptors.

B Cell-Specific AhR Deficiency Causes Exacerbated Arthritis and Increased T Cell-Driven Arthritogenic Responses

Having confirmed the contribution of AhR in the programming of the $IL\text{-}10^{+}CD19^{+}CD21^{hi}CD24^{hi}$Breg transcriptional profile, we explored the impact of AhR deficiency specifically in B cells on the immune response associated with arthritis. $Ahr^{fl/-}Mb1^{cre/+}$ mice developed exacerbated arthritis compared with control $Mb1^{cre/+}$ mice (Figure 5A). Histological analysis of joint tissue showed an increase in immune cell infiltration in the synovia and hyper-vascularization in $Ahr^{fl/-}Mb1^{cre/+}$ compared with control $Mb1^{cre/+}$ mice (Figure 5B). The enhanced inflammation was associated with a significant increase in the frequency and number of IFN-γ- and IL-17-expressing $CD4^{+}$ T cells in the spleen and DLN (Figures 5C–5F). $Ahr^{fl/-}Mb1^{cre/+}$ mice had an increased frequency and number of $IL\text{-}17^{+}CD4^{+}$ cells and a reduction in the frequency and total number of $Foxp3^{+}$Tregs in the inguinal DLN compared with the control mice (Figures 5F–5H). Increased levels of IL-17 were observed in the synovium of inflamed joints of $Ahr^{fl/-}Mb1^{cre/+}$ compared with the control group, whereas IFN-γ levels were undetectable (Figure 5I).

Adoptive transfer of $Mb1^{cre/+}$ or $Ahr^{fl/-}Mb1^{cre/+}$ $CD19^{+}$ $CD21^{hi}CD24^{hi}$B cells into syngeneic mice showed that only $CD19^{+}CD21^{hi}CD24^{hi}$B cells from $Mb1^{cre/+}$, but not from $Ahr^{fl/-}Mb1^{cre/+}$ mice, significantly inhibited disease and Th17/Th1 differentiation in the recipient mice (Figures 5J–5L), confirming that $CD19^{+}CD21^{hi}CD24^{hi}$B cells were less effective at suppressing inflammation in the absence of AhR.

$Ahr^{fl/-}Mb1^{cre/+}$ Mice Do Not Have a Defect in B Cell Development but Present with a Reduced Frequency and Number of Bregs

To establish that the increase in disease severity and the impact on the T cell compartment was due to the lack of AhR-expressing Bregs, rather than a consequence of abnormal B cell development, we next compared the frequencies of pro, pro-pre, pre, immature, transitional (T), early, and late mature B cells in the bone marrow or T1 or FO B cells in the spleens of $Ahr^{fl/-}Mb1^{cre/+}$ and $Mb1^{cre/+}$ mice. We observed no differences in these populations, suggesting that the increase in arthritis severity and in pro-inflammatory T cells was indeed due to a reduction of $CD19^{+}CD21^{hi}CD24^{hi}$Bregs rather than a consequence of abnormal B cell development (Figures 6A and 6B; Figures S5A–S5H). In line with previous findings showing that AhR represses differentiation of B cells into plasma cells (Tucker et al., 1986), we found increased frequencies of splenic plasma cells (Figures S5I and S5J) and increased *Prdm1* mRNA expression in $Ahr^{fl/-}Mb1^{cre/+}$ B cells relative to $Mb1^{cre/+}$ B cells (Figure S5K). Despite changes in the frequency of plasma cells in the spleens, there were no differences in the amounts of secreted IgG, IgM, and IgA in the serum of arthritic $Ahr^{fl/-}Mb1^{cre/+}$ versus $Mb1^{cre/+}$ mice (Figure S5L).

Although there were no differences in the number of splenic $CD19^{+}CD21^{hi}CD24^{hi}$B cells between the two groups, $Ahr^{fl/-}Mb1^{cre/+}CD21^{hi}CD24^{hi}$B cells secreted significantly less IL-10 than $Mb1^{cre/+}CD19^{+}CD21^{hi}CD24^{hi}$B cells, following stimulation with LPS+anti-IgM (Figure 6C). IL-10 production by $Ahr^{fl/-}Mb1^{cre/+}$ $CD19^{+}$B cells was significantly reduced in response to TLR9 stimulation (Figures S6A–S6C). Comparable levels of *Ebi3* and *Il12a* were present in B cells from $Ahr^{fl/-}Mb1^{cre/+}$ mice and $Mb1^{cre/+}$ mice (Figures S6D and S6E). These results, together with our previous findings showing a redundant effect of IL-35 in Bregs in this model (Vasconcellos et al., 2011), excluded that Bregs were non-functional because of a lack of IL-35 production in the absence of AhR.

The observed IL-10 reduction was not due to impaired B cell proliferation, as an equivalent expression of Ki-67 in $Mb1^{cre/+}$ and $Ahr^{fl/-}Mb1^{cre/+}$ $CD19^{+}CD21^{hi}CD24^{hi}$B cells was observed both directly *ex vivo* after antigen-induced arthritis (AIA) and after stimulation with LPS+anti-IgM (Figures S6F–S6I). No difference in genes related to the cell cycle was observed, including *Ccno*, previously been shown to be regulated by AhR in splenic B cells (Villa et al., 2017), between $Mb1^{cre/+}$ and $Ahr^{fl/-}Mb1^{cre/+}$ $CD19^{+}CD21^{hi}CD24^{hi}$B cells (Figure S6J), suggesting that there was no impairment in BCR-driven regulation of B cell entry into the cell cycle (Richards et al., 2008).

Mesenteric lymph nodes (MLNs) are important sites for the licensing of Breg development, through the exposure to microbiota-derived pro-inflammatory signals IL-1β and IL-6 (Rosser et al., 2014). Fewer $CD19^{+}CD21^{hi}CD24^{hi}$B cells, and a decreased amount of B cell-derived IL-10 after *in vitro* polarization with LPS+anti-IgM, were observed in the MLN of $Ahr^{fl/-}Mb1^{cre/+}$ mice compared with control $Mb1^{cre/+}$ mice (Figures 6D–6F). Of interest, no difference in gut-homing integrin α4β7

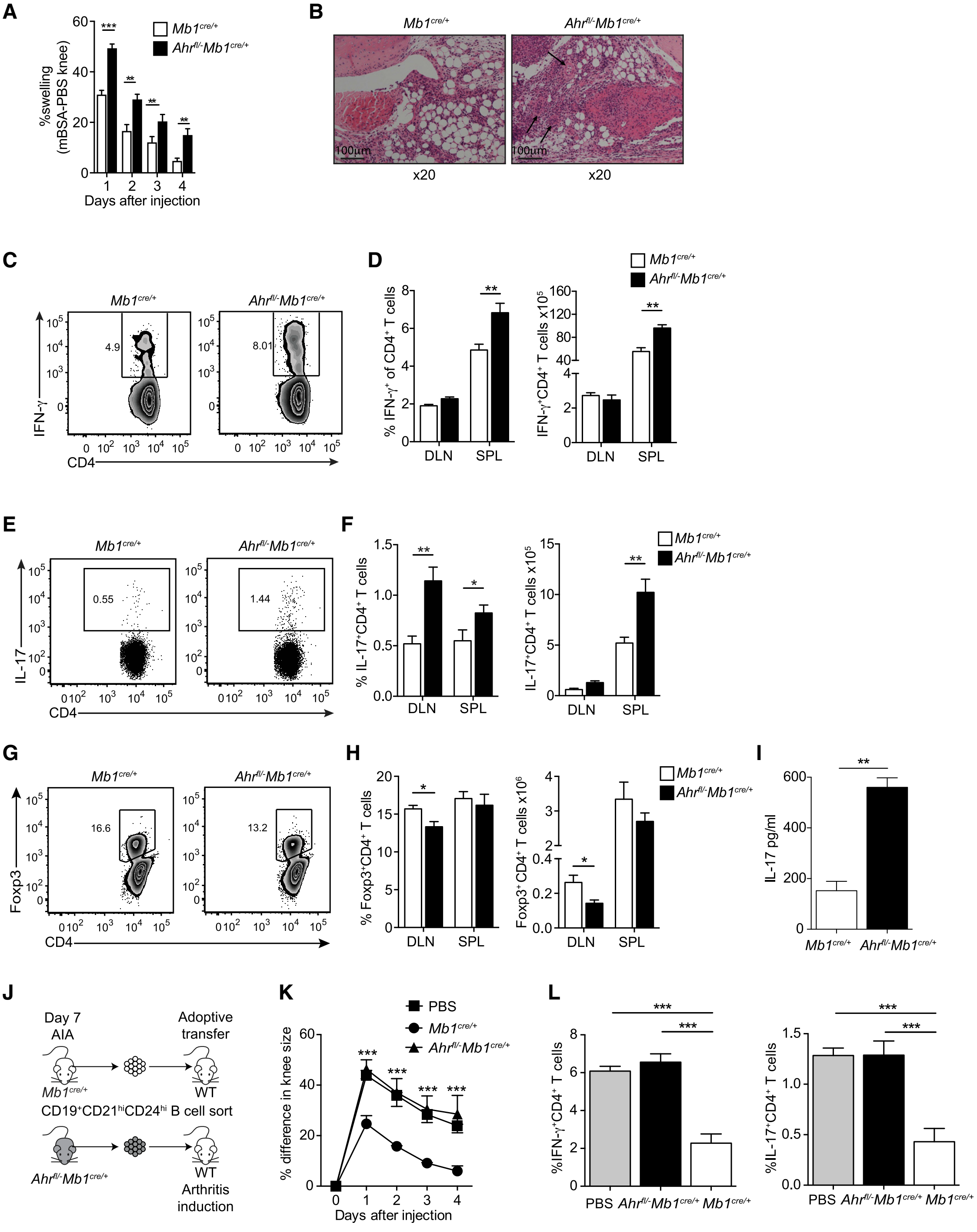

Figure 5. B Cell AhR Deficiency Exacerbates Antigen Induced Arthritis

(A) Mean clinical score of *Mb1*$^{cre/+}$ and *Ahr*$^{fl/-}$*Mb1*$^{cre/+}$ mice following induction of arthritis; y axis shows percentage swelling in antigen-injected knee compared with control knee (n = 12 per group).

(B) Representative H&E staining of arthritic joints from *Mb1*$^{cre/+}$ and *Ahr*$^{fl/-}$*Mb1*$^{cre/+}$ mice (n = 3; original magnification of 20×). Arrows indicate hyper-vascularization. Scale bar, 100 μM.

(C–H) Representative flow cytometry plots and bar charts showing respectively the percentage and number of (C and D) IFN-γ^{+}CD4^{+} cells, (E and F) IL-17^{+}CD4^{+} cells, and (G and H) Foxp3^{+}CD4^{+}T cells in the spleens and DLNs of *Mb1*$^{cre/+}$ and *Ahr*$^{fl/-}$*Mb1*$^{cre/+}$ mice (n = 7).

(I) IL-17 concentration as measured in the synovial fluid of *Mb1*$^{cre/+}$ and *Ahr*$^{fl/-}$*Mb1*$^{cre/+}$ mice (n = 6).

(J) Schematic showing the experimental design of the adoptive transfer system.

(legend continued on next page)

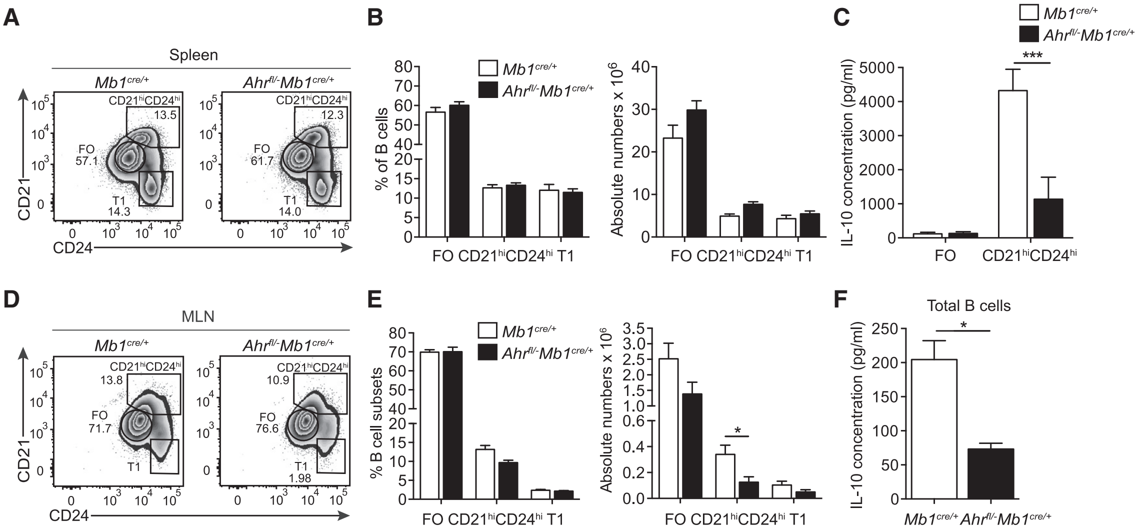

Figure 6. CD19^{+}CD21^{hi}CD24^{hi}B Cells in *Ahr*$^{fl/-}$*Mb1*$^{cre/+}$ Mice Are Less Able to Differentiate into Bregs

AIA was induced in *Mb1*$^{cre/+}$ and *Ahr*$^{fl/-}$*Mb1*$^{cre/+}$ mice.

(A) Representative flow cytometry plots showing percentage and (B) bar charts showing the percentages and absolute numbers of CD19^{+}CD21^{-}CD24^{hi} (T1), CD19^{+}CD21^{hi}CD24^{hi}, and FO B cells in the spleens of *Mb1*$^{cre/+}$ and *Ahr*$^{fl/-}$*Mb1*$^{cre/+}$ mice (n = 7).

(C) CD19^{+}CD21^{hi}CD24^{hi} or FO B cells were sorted from *Mb1*$^{cre/+}$ and *Ahr*$^{fl/-}$*Mb1*$^{cre/+}$ mice and stimulated with LPS+anti-IgM for 48 h. IL-10 production, as measured by ELISA (n = 4 per group).

(D and E) Representative flow cytometry plots (D) showing percentage and (E) bar charts showing percentages and absolute numbers of CD19^{+}CD21^{-}CD24^{hi}, CD19^{+}CD21^{hi}CD24^{hi} and FO B cells in the MLNs of *Mb1*$^{cre/+}$ and *Ahr*$^{fl/-}$*Mb1*$^{cre/+}$ mice (n = 7).

(F) CD19^{+}B cells were sorted from *Mb1*$^{cre/+}$ and *Ahr*$^{fl/-}$*Mb1*$^{cre/+}$ mice and stimulated with LPS+anti-IgM for 48 h. IL-10 production, as measured by ELISA (n = 3).

All experiments were performed at day 7 post-IA injection. Data representative of at least two independent experiments with biological replicates. In (B), (C), (E), and (F), data are expressed as mean ± SEM. *p < 0.05, **p < 0.01, and ***p < 0.01, two-way ANOVA and Mann-Whitney test.

See also Figures S5–S7.

expression was observed in splenic or MLN-derived CD19^{+}CD21^{hi}CD24^{hi}B cells between *Ahr*$^{fl/-}$*Mb1*$^{cre/+}$ and *Mb1*$^{cre/+}$ mice, suggesting that the reduction of Bregs observed in the MLN of *Ahr*$^{fl/-}$*Mb1*$^{cre/+}$ mice is $\alpha4\beta7$ independent (Figures S6K–S6M). We can exclude that the decreased frequency of Bregs was the consequence of a reduction in monocyte-derived IL-1β and IL-6 produced in the spleens or in the MLNs, as equivalent amounts of these cytokines were produced by these cells in both *Ahr*$^{fl/-}$*Mb1*$^{cre/+}$ and *Mb1*$^{cre/+}$ mice (Figures S7A–S7D).

Thus, our results collectively show that mice lacking AhR expression in B cells phenocopy the results we have previously observed in mice with IL-10$^{-/-}$B cells (Carter et al., 2011) and influence IL-10^{+}CD19^{+}CD21^{hi}CD24^{hi}Breg differentiation in a cell-intrinsic manner.

DISCUSSION

Bregs are generated in the periphery in response to a variety of "homeostatic" inflammatory stimuli including activation through TLRs and by pro-inflammatory cytokines (Rosser and Mauri, 2015). The transcriptional program that governs the differentiation and function of IL-10^{+}CD19^{+}CD21^{hi}CD24^{hi}Bregs remains virtually unknown. Here, we show that AhR contributes to the differentiation of CD19^{+}CD21^{hi}CD24^{hi}B cells into functionally suppressive IL-10^{+}CD19^{+}CD21^{hi}CD24^{hi}Bregs by regulating their IL-10 production and by repressing the transcription of pro-inflammatory mediators. The importance of IL-10 in mediating the suppressive effect of Bregs is well established, and its role is corroborated by *in vivo* results showing that mice lacking IL-10-producing B cells develop exacerbated autoimmunity (Carter et al., 2011). Similarly, AhR deficiency restricted to B cells impairs IL-10^{+}CD19^{+}CD21^{hi}CD24^{hi}Breg differentiation and function, resulting in an increase of IFNγ and IL-17-expressing CD4^{+} T cells, a decrease in Tregs, and the development of an exacerbated arthritis.

AhR plays a pleiotropic role in the regulation of several immune responses (Stockinger et al., 2014), most notably in the differentiation of CD4^{+} T cells, in which AhR influences both the differentiation and activation of Th17 cells, which are known to play a major role in the pathogenesis of several autoimmune diseases (Stockinger and Omenetti, 2017) and the differentiation of CD4^{+} T cells into Tr1 cells (Apetoh et al., 2010). Immune

(K) Mean clinical score of C57BL/6 mice following adoptive transfer of CD19^{+}CD21^{hi}CD24^{hi}B cells purified from *Mb1*$^{cre/+}$ and *Ahr*$^{fl/-}$*Mb1*$^{cre/+}$ mice, administered on the day of disease onset. Control (no transfer) group received PBS (n = 5).

(L) Bar charts showing respectively the percentage of IFN-γ^{+}CD4^{+} cells and IL-17^{+}CD4^{+} cells in the spleens of WT recipient mice, following an adoptive transfer of *Mb1*$^{cre/+}$ and *Ahr*$^{fl/-}$*Mb1*$^{cre/+}$ CD19^{+}CD21^{hi}CD24^{hi}B cells or a PBS control (n = 5).

All experiments were performed at day 7 post-IA injection. Data are representative of at least three independent experiments with biological replicates. In (A), (D), (F), (H), (I), (K), and (L), data are expressed as mean ± SEM. *p < 0.05 and **p < 0.01, one- and two-way ANOVA and Mann-Whitney test.

suppression was one of the earliest known observations of AhR function. 2,3,7,8-Tetrachlorodibenzo-ρ-dioxin (TCDD), an environmental contaminant and a potent AhR agonist, was found to suppress delayed hypersensitivity (DTH) responses to tuberculin (Vos et al., 1973). More recently, it has become apparent that AhR has a conserved role in the regulation of IL-10 across the innate and adaptive immune system, controlling IL-10 production in natural killer cells (Wagage et al., 2014), peritoneal (Kimura et al., 2009) and bone marrow-derived macrophages (Shinde et al., 2018), and Tr1 cells, in which AhR binding to the *Il10* promoter region has been described (Apetoh et al., 2010). We demonstrate that in IL-10$^+$B cells, AhR binds upstream of the *Il10* TSS to a different genomic region than in Tr1 cells, suggesting that there are cell context- and cell signal-specific epigenetic differences in the regulation of *Il10* (Saraiva et al., 2005).

An interesting finding in our study was the discovery that AhR partly controls the differentiation of CD19$^+$CD21^{hi}CD24^{hi}B cells into a polarized IL-10$^+$CD19$^+$CD21^{hi}CD24^{hi}Breg population that produces only IL-10 by contributing to IL-10 induction and by suppressing the transcription of several pro-inflammatory cytokines, such as *Il2*, *Il6*, and *Tnf*. AhR has been previously shown to inhibit pro-inflammatory IL-17 and IFN-γ cytokine production in T cells and to induce Tr1 cell differentiation in the gut (Ye et al., 2017). In addition, in the absence of AhR in macrophages, mice are more susceptible to LPS-induced endotoxic shock and present with an increase in pro-inflammatory IL-6 expression (Kimura et al., 2009). AhR deletion in microglial cells led to the upregulation of *Ccl2*, *Il1b*, *Nos2*, and *Vegfb* gene expression, factors known to be involved in inflammation and neurodegeneration (Rothhammer et al., 2018). Our data reveal that AhR preserves the immunosuppressive function of splenic IL-10$^+$CD19$^+$CD21^{hi}CD24^{hi}Bregs by silencing a pro-inflammatory transcriptional program. Whether AhR complexes bind to XRE on the loci of pro-inflammatory cytokines and directly inhibit their expression in B cells, or if AhR coordinates the suppression of pro-inflammatory immune responses through interaction with other transcription factors in B cells, warrants further study.

Our adoptive transfer results suggest that the predominant effect of AhR loss in B cells on AIA is through the loss of IL-10. We have previously shown that adoptive transfer of IL-10$^{-/-}$ B cells is unable to suppress arthritis (Carter et al., 2011; Evans et al., 2007). Here we show that WT mice do not get worse disease than the PBS control-injected mice upon adoptive transfer of AhR$^-$CD19$^+$CD21^{hi}CD24^{hi} B cells. Equally, AhR$^-$CD19$^+$CD21^{hi}CD24^{hi} B cells do not suppress disease onset in the recipient mice, unlike the transfer of control AhR$^+$CD19$^+$CD21^{hi}CD24^{hi} B cells. These data therefore suggest that the deleterious effects observed in AIA is the consequence of the reduced amount of IL-10. However, we cannot rule out the possibility that *in vivo*, AhR-deficient B cells contribute to overall inflammation through the upregulation of pro-inflammatory cytokines and chemokines or indirectly through the recruitment of other cell types.

We have recently reported that AhR deletion, rather than impairing the capacity of B cells to proliferate, instead compromised their ability to commence the cell cycle (Villa et al., 2017). Indeed, there was reduction in *Ccno* mRNA expression in splenic B cells isolated from naive *Ahr*$^{fl/-}$*Mb1*$^{cre/+}$ compared with *Mb1*$^{cre/+}$ mice. Here we show that this defect is overcome during an arthritogenic response, as no change in Ki-67 expression or in genes regulating the cell cycle, including *Ccno*, were observed between *Mb1*$^{cre/+}$ and *Ahr*$^{fl/-}$*Mb1*$^{cre/+}$ CD19$^+$CD21^{hi}CD24^{hi}B cells taken directly from arthritic mice. Furthermore, no difference was observed in Ki-67 expression between *Mb1*$^{cre/+}$ and *Ahr*$^{fl/-}$*Mb1*$^{cre/+}$ CD19$^+$CD21^{hi}CD24^{hi}B cells after restimulation with LPS+anti-IgM. Although, in the latter, we used purified CD19$^+$CD21^{hi}CD24^{hi} B cells instead of total splenic B cells and used different stimuli to activate this population, which together might account for the differences observed in this study. Therefore, these data confirmed that the phenotype observed in the absence of AhR expression in B cells is not due to impaired B cell proliferation but instead is due to the reduced ability of B cells to differentiate into Bregs and reduction in the production of IL-10.

Our results show that although the loss of B cell AhR expression reduces IL-10$^+$CD19$^+$CD21^{hi}CD24^{hi}Breg frequency and leads to an expansion of plasma cells, it does not affect the frequencies or absolute numbers of B cell populations up to a mature naive B cell stage. Taken together with previous findings showing that AhR is expressed by B cells from the immature B cell stage in the bone marrow (Villa et al., 2017; Yamaguchi et al., 1997a, 1997b), we suggest that B cell AhR expression is important primarily for the control of IL-10$^+$CD19$^+$CD21^{hi}CD24^{hi} Breg immune-regulatory transcriptional programming and restricting plasma cell development but is dispensable for homeostatic B cell development. We report that *Ahr*$^{fl/-}$*Mb1*$^{cre/+}$ mice have increased frequencies of splenic plasma cells compared with control *Mb1*$^{cre/+}$ mice, and these results are in line with those showing that both prototypic AhR agonists (polycyclic aromatic and planar halogenated hydrocarbons) affect terminal differentiation of B cells and humoral immune responses by inhibiting plasma cell differentiation and reducing the production of IgM (Schneider et al., 2009; Tucker et al., 1986; Vaidyanathan et al., 2017). We find increased levels of *Prdm1* (Blimp-1) mRNA expression in B cells lacking AhR, consistent with previous findings showing that the suppression of terminal differentiation is mediated through AhR increases Bach2 expression (De Abrew et al., 2011); Bach2 in turn represses the expression of Blimp-1, a key transcription factor that controls B cell differentiation into immunoglobulin-producing plasma cells (Turner et al., 1994). We have extended the significance of these results to an inflammatory model and shown that early B cell development and maturation of B cells is not affected by B cell AhR expression but that AhR is required for the differentiation of IL-10$^+$CD19$^+$CD21^{hi}CD24^{hi}Bregs. It is tantalizing to propose that the increase in plasma cells observed in mice lacking AhR$^+$IL-10$^+$CD19$^+$CD21^{hi}CD24^{hi}Bregs is due to the impaired function of Bregs. We have previously shown that at least in humans, IL-10 produced by Bregs directly inhibits T helper cell differentiation, which prevents plasma cell differentiation (Menon et al., 2016).

Here we have shown that AhR, in response to inflammatory signals, plays an important role in the homeostatic maintenance of Breg function by acting as a molecular brake, preventing the differentiation of Bregs into effector B cells producing pro-inflammatory mediators. In addition to identifying that AhR regulates IL-10 expression in Bregs, our data

highlight an additional mechanism by which AhR restrains inflammatory responses. These results add to a growing body of evidence supporting AhR as a key modulator of immune tolerance and therefore a potential therapeutic target in autoimmunity.

STAR★METHODS

Detailed methods are provided in the online version of this paper and include the following:

- KEY RESOURCES TABLE
- LEAD CONTACT AND MATERIALS AVAILABILITY
- EXPERIMENTAL MODEL AND SUBJECT DETAILS
  - Induction of antigen-induced arthritis (AIA)
- METHOD DETAILS
  - Cell isolation and culture
  - ELISA's on cell culture supernatants and sera
  - Flow cytometry and cell sorting
  - In-vitro suppression assay
  - Adoptive transfer experiments
  - Histology
  - Microarray analysis and RNA extraction
  - Chromatin Immunoprecipitation
  - Western blot
  - RNA-seq
  - Bioinformatic analysis of RNA-seq data
  - ATAC-seq
  - Bioinformatic analysis of ATAC-seq data
  - qPCR
- QUANTIFICATION AND STATISTICAL ANALYSIS
- DATA AND CODE AVAILABILITY

SUPPLEMENTAL INFORMATION

Supplemental Information can be found online at https://doi.org/10.1016/j.celrep.2019.10.018.

ACKNOWLEDGMENTS

We would like to thank Dr. Chris Karp (Bill and Melinda Gates Foundation) for providing the Vert-X mice; Professor Michael Reth (Max Planck Institute) for *Mb1*^{cre} mice; Professor Werner Muller (University of Manchester) for IL-10R$^{-/-}$ mice; the staff of the University College London Biological Services Unit for all the help with animal husbandry; Jamie Evans for cell sorting; Drs. Diego Catalán and Amanda Duhlin for critically reviewing the manuscript; Drs. Tina Baker and Leo Swadling for help with analysis of the RNA-seq dataset; and Professor Idit Shachar, Lihi Radomir, and the Crown Institute for Genomics in the Nancy and Stephen Grand Israel National Center for Personalized Medicine (G-INCPM) for the library preparation and sequencing of the RNA-seq samples. This work is funded by a Versus Arthritis program grant (21140) to C.M. and the European Community's Seventh Framework Programme (FP7-2007-2013) under grant agreement HEALTH-F2-2013-602114 (Athero-B-Cell) to C.M. and I.D. C.J.M.P is funded by a Rosetrees Trust PhD studentship (M536). E.C.R. is funded by a Versus Arthritis UK Foundation fellowship (21141). K.N. and part of this work are funded by a Wellcome Trust Intermediate Clinical Fellowship (097259/Z/11/Z). B.S. is supported by the Francis Crick Institute, which receives its core funding from Cancer Research UK (FC001159), The UK Medical Research Council (FC001159), and the Wellcome Trust (FC001159).

AUTHOR CONTRIBUTIONS

C.J.M.P. designed and performed experiments, analyzed data, and wrote the manuscript. E.C.R. performed experiments, analyzed data, and critically reviewed the manuscript. K.O. analyzed data and wrote the manuscript. T.K., A.F.R., and C.B. performed the bioinformatics analysis of the ATAC-seq data and critically reviewed the manuscript. A.B. and G.X. performed the ChIP and critically reviewed the manuscript. I.D. performed the bioinformatics analysis of the expression microarray and RNA-seq datasets. K.N. and S.T. performed some of the experiments and analyzed data. B.S. and M.V. generated the *Ahr*$^{fl/-}$*Mb1*$^{cre/+}$ deficient mice, helped design the experiments, and critically reviewed the manuscript. C.M. designed the study, analyzed data, and wrote the manuscript.

DECLARATION OF INTERESTS

The authors declare no competing interests.

Received: January 15, 2019
Revised: August 23, 2019
Accepted: October 3, 2019
Published: November 12, 2019

REFERENCES

Anders, S., Pyl, P.T., and Huber, W. (2015). HTSeq—a Python framework to work with high-throughput sequencing data. Bioinformatics *31*, 166–169.

Apetoh, L., Quintana, F.J., Pot, C., Joller, N., Xiao, S., Kumar, D., Burns, E.J., Sherr, D.H., Weiner, H.L., and Kuchroo, V.K. (2010). The aryl hydrocarbon receptor interacts with c-Maf to promote the differentiation of type 1 regulatory T cells induced by IL-27. Nat. Immunol. *11*, 854–861.

Blair, P.A., Noreña, L.Y., Flores-Borja, F., Rawlings, D.J., Isenberg, D.A., Ehrenstein, M.R., and Mauri, C. (2010). CD19(+)CD24(hi)CD38(hi) B cells exhibit regulatory capacity in healthy individuals but are functionally impaired in systemic Lupus Erythematosus patients. Immunity *32*, 129–140.

Bray, N.L., Pimentel, H., Melsted, P., and Pachter, L. (2016). Near-optimal probabilistic RNA-seq quantification. Nat. Biotechnol. *34*, 525–527.

Brummel, R., and Lenert, P. (2005). Activation of marginal zone B cells from lupus mice with type A(D) CpG-oligodeoxynucleotides. J. Immunol. *174*, 2429–2434.

Carter, N.A., Vasconcellos, R., Rosser, E.C., Tulone, C., Muñoz-Suano, A., Kamanaka, M., Ehrenstein, M.R., Flavell, R.A., and Mauri, C. (2011). Mice lacking endogenous IL-10-producing regulatory B cells develop exacerbated disease and present with an increased frequency of Th1/Th17 but a decrease in regulatory T cells. J. Immunol. *186*, 5569–5579.

De Abrew, K.N., Phadnis, A.S., Crawford, R.B., Kaminski, N.E., and Thomas, R.S. (2011). Regulation of Bach2 by the aryl hydrocarbon receptor as a mechanism for suppression of B-cell differentiation by 2,3,7,8-tetrachlorodibenzo-p-dioxin. Toxicol. Appl. Pharmacol. *252*, 150–158.

Denaës, T., Lodder, J., Chobert, M.N., Ruiz, I., Pawlotsky, J.M., Lotersztajn, S., and Teixeira-Clerc, F. (2016). The cannabinoid receptor 2 protects against alcoholic liver disease via a macrophage autophagy-dependent pathway. Sci. Rep. *6*, 28806.

Dobin, A., Davis, C.A., Schlesinger, F., Drenkow, J., Zaleski, C., Jha, S., Batut, P., Chaisson, M., and Gingeras, T.R. (2013). STAR: ultrafast universal RNA-seq aligner. Bioinformatics *29*, 15–21.

Evans, J.G., Chavez-Rueda, K.A., Eddaoudi, A., Meyer-Bahlburg, A., Rawlings, D.J., Ehrenstein, M.R., and Mauri, C. (2007). Novel suppressive function of transitional 2 B cells in experimental arthritis. J. Immunol. *178*, 7868–7878.

Feige, U., Hu, Y.L., Gasser, J., Campagnuolo, G., Munyakazi, L., and Bolon, B. (2000). Anti-interleukin-1 and anti-tumor necrosis factor-alpha synergistically inhibit adjuvant arthritis in Lewis rats. Cell. Mol. Life Sci. *57*, 1457–1470.

Fillatreau, S., Sweenie, C.H., McGeachy, M.J., Gray, D., and Anderton, S.M. (2002). B cells regulate autoimmunity by provision of IL-10. Nat. Immunol. *3*, 944–950.

Gray, M., Miles, K., Salter, D., Gray, D., and Savill, J. (2007). Apoptotic cells protect mice from autoimmune inflammation by the induction of regulatory B cells. Proc. Natl. Acad. Sci. U S A *104*, 14080–14085.

Hao, S., Han, X., Wang, D., Yang, Y., Li, Q., Li, X., and Qiu, C.H. (2016). Critical role of CCL22/CCR4 axis in the maintenance of immune homeostasis during apoptotic cell clearance by splenic CD8α(+) CD103(+) dendritic cells. Immunology *148*, 174–186.

Heath-Pagliuso, S., Rogers, W.J., Tullis, K., Seidel, S.D., Cenijn, P.H., Brouwer, A., and Denison, M.S. (1998). Activation of the Ah receptor by tryptophan and tryptophan metabolites. Biochemistry *37*, 11508–11515.

Imai, T., Nagira, M., Takagi, S., Kakizaki, M., Nishimura, M., Wang, J., Gray, P.W., Matsushima, K., and Yoshie, O. (1999). Selective recruitment of CCR4-bearing Th2 cells toward antigen-presenting cells by the CC chemokines thymus and activation-regulated chemokine and macrophage-derived chemokine. Int. Immunol. *11*, 81–88.

Iyer, S.S., and Cheng, G. (2012). Role of interleukin 10 transcriptional regulation in inflammation and autoimmune disease. Crit. Rev. Immunol. *32*, 23–63.

Kanehisa, M., Sato, Y., Kawashima, M., Furumichi, M., and Tanabe, M. (2016). KEGG as a reference resource for gene and protein annotation. Nucleic Acids Res. *44* (*D1*), D457–D462.

Kasama, T., Strieter, R.M., Lukacs, N.W., Lincoln, P.M., Burdick, M.D., and Kunkel, S.L. (1995). Interleukin-10 expression and chemokine regulation during the evolution of murine type II collagen-induced arthritis. J. Clin. Invest. *95*, 2868–2876.

Kauffmann, A., Gentleman, R., and Huber, W. (2009). arrayQualityMetrics—a bioconductor package for quality assessment of microarray data. Bioinformatics *25*, 415–416.

Khan, A., Fornes, O., Stigliani, A., Gheorghe, M., Castro-Mondragon, J.A., van der Lee, R., Bessy, A., Chèneby, J., Kulkarni, S.R., Tan, G., et al. (2018). JASPAR 2018: update of the open-access database of transcription factor binding profiles and its web framework. Nucleic Acids Res. *46* (*D1*), D260–D266.

Kimura, A., Naka, T., Nakahama, T., Chinen, I., Masuda, K., Nohara, K., Fujii-Kuriyama, Y., and Kishimoto, T. (2009). Aryl hydrocarbon receptor in combination with Stat1 regulates LPS-induced inflammatory responses. J. Exp. Med. *206*, 2027–2035.

Klinker, M.W., Reed, T.J., Fox, D.A., and Lundy, S.K. (2013). Interleukin-5 supports the expansion of fas ligand-expressing killer B cells that induce antigen-specific apoptosis of CD4(+) T cells and secrete interleukin-10. PLoS ONE *8*, e70131.

Lampropoulou, V., Hoehlig, K., Roch, T., Neves, P., Calderón Gómez, E., Sweenie, C.H., Hao, Y., Freitas, A.A., Steinhoff, U., Anderton, S.M., and Fillatreau, S. (2008). TLR-activated B cells suppress T cell-mediated autoimmunity. J. Immunol. *180*, 4763–4773.

Li, S., Miao, T., Sebastian, M., Bhullar, P., Ghaffari, E., Liu, M., Symonds, A.L., and Wang, P. (2012). The transcription factors Egr2 and Egr3 are essential for the control of inflammation and antigen-induced proliferation of B and T cells. Immunity *37*, 685–696.

Lino, A.C., Dang, V.D., Lampropoulou, V., Welle, A., Joedicke, J., Pohar, J., Simon, Q., Thalmensi, J., Baures, A., Fluhler, V., et al. (2018). LAG-3 inhibitory receptor expression identifies immunosuppressive natural regulatory plasma cells. Immunity *49*, 120–133.e9.

Liu, D., Yin, X., Olyha, S.J., Nascimento, M.S.L., Chen, P., White, T., Gowthaman, U., Zhang, T., Gertie, J.A., Zhang, B., et al. (2019). IL-10-dependent crosstalk between murine marginal zone B cells, macrophages, and CD8alpha(+) dendritic cells promotes Listeria monocytogenes infection. Immunity *51*, 64–76.e7.

Madan, R., Demircik, F., Surianarayanan, S., Allen, J.L., Divanovic, S., Trompette, A., Yogev, N., Gu, Y., Khodoun, M., Hildeman, D., et al. (2009). Nonredundant roles for B cell-derived IL-10 in immune counter-regulation. J. Immunol. *183*, 2312–2320.

Martins, G.A., Cimmino, L., Liao, J., Magnusdottir, E., and Calame, K. (2008). Blimp-1 directly represses Il2 and the Il2 activator Fos, attenuating T cell proliferation and survival. J. Exp. Med. *205*, 1959–1965.

Matsumoto, M., Fujii, Y., Baba, A., Hikida, M., Kurosaki, T., and Baba, Y. (2011). The calcium sensors STIM1 and STIM2 control B cell regulatory function through interleukin-10 production. Immunity *34*, 703–714.

Matsumoto, M., Baba, A., Yokota, T., Nishikawa, H., Ohkawa, Y., Kayama, H., Kallies, A., Nutt, S.L., Sakaguchi, S., Takeda, K., et al. (2014). Interleukin-10-producing plasmablasts exert regulatory function in autoimmune inflammation. Immunity *41*, 1040–1051.

Mauri, C., Mars, L.T., and Londei, M. (2000). Therapeutic activity of agonistic monoclonal antibodies against CD40 in a chronic autoimmune inflammatory process. Nat. Med. *6*, 673–679.

McClintick, J.N., and Edenberg, H.J. (2006). Effects of filtering by Present call on analysis of microarray experiments. BMC Bioinformatics *7*, 49.

Menon, M., Blair, P.A., Isenberg, D.A., and Mauri, C. (2016). A regulatory feedback between plasmacytoid dendritic cells and regulatory B cells is aberrant in systemic lupus erythematosus. Immunity *44*, 683–697.

Mohan, K., and Issekutz, T.B. (2007). Blockade of chemokine receptor CXCR3 inhibits T cell recruitment to inflamed joints and decreases the severity of adjuvant arthritis. J. Immunol. *179*, 8463–8469.

Nakayama, T., Hieshima, K., Nagakubo, D., Sato, E., Nakayama, M., Kawa, K., and Yoshie, O. (2004). Selective induction of Th2-attracting chemokines CCL17 and CCL22 in human B cells by latent membrane protein 1 of Epstein-Barr virus. J. Virol. *78*, 1665–1674.

Nistala, K., Adams, S., Cambrook, H., Ursu, S., Olivito, B., de Jager, W., Evans, J.G., Cimaz, R., Bajaj-Elliott, M., and Wedderburn, L.R. (2010). Th17 plasticity in human autoimmune arthritis is driven by the inflammatory environment. Proc. Natl. Acad. Sci. U S A *107*, 14751–14756.

Ohshima, S., Saeki, Y., Mima, T., Sasai, M., Nishioka, K., Nomura, S., Kopf, M., Katada, Y., Tanaka, T., Suemura, M., and Kishimoto, T. (1998). Interleukin 6 plays a key role in the development of antigen-induced arthritis. Proc. Natl. Acad. Sci. U S A *95*, 8222–8226.

Oleinika, K., Rosser, E.C., Matei, D.E., Nistala, K., Bosma, A., Drozdov, I., and Mauri, C. (2018). CD1d-dependent immune suppression mediated by regulatory B cells through modulations of iNKT cells. Nat. Commun. *9*, 684.

Pils, M.C., Pisano, F., Fasnacht, N., Heinrich, J.M., Groebe, L., Schippers, A., Rozell, B., Jack, R.S., and Müller, W. (2010). Monocytes/macrophages and/or neutrophils are the target of IL-10 in the LPS endotoxemia model. Eur. J. Immunol. *40*, 443–448.

Rendeiro, A.F., Schmidl, C., Strefford, J.C., Walewska, R., Davis, Z., Farlik, M., Oscier, D., and Bock, C. (2016). Chromatin accessibility maps of chronic lymphocytic leukaemia identify subtype-specific epigenome signatures and transcription regulatory networks. Nat. Commun. *7*, 11938.

Richards, S., Watanabe, C., Santos, L., Craxton, A., and Clark, E.A. (2008). Regulation of B-cell entry into the cell cycle. Immunol. Rev. *224*, 183–200.

Ritchie, M.E., Phipson, B., Wu, D., Hu, Y., Law, C.W., Shi, W., and Smyth, G.K. (2015). limma powers differential expression analyses for RNA-sequencing and microarray studies. Nucleic Acids Res. *43*, e47.

Robinson, M.D., and Smyth, G.K. (2008). Small-sample estimation of negative binomial dispersion, with applications to SAGE data. Biostatistics *9*, 321–332.

Robinson, M.D., McCarthy, D.J., and Smyth, G.K. (2010). edgeR: a Bioconductor package for differential expression analysis of digital gene expression data. Bioinformatics *26*, 139–140.

Rosser, E.C., and Mauri, C. (2015). Regulatory B cells: origin, phenotype, and function. Immunity *42*, 607–612.

Rosser, E.C., Oleinika, K., Tonon, S., Doyle, R., Bosma, A., Carter, N.A., Harris, K.A., Jones, S.A., Klein, N., and Mauri, C. (2014). Regulatory B cells are induced by gut microbiota-driven interleukin-1β and interleukin-6 production. Nat. Med. *20*, 1334–1339.

Rothhammer, V., Borucki, D.M., Tjon, E.C., Takenaka, M.C., Chao, C.C., Ardura-Fabregat, A., de Lima, K.A., Gutiérrez-Vázquez, C., Hewson, P., Staszewski, O., et al. (2018). Microglial control of astrocytes in response to microbial metabolites. Nature *557*, 724–728.

Ruth, J.H., Haas, C.S., Park, C.C., Amin, M.A., Martinez, R.J., Haines, G.K., 3rd, Shahrara, S., Campbell, P.L., and Koch, A.E. (2006). CXCL16-mediated cell recruitment to rheumatoid arthritis synovial tissue and murine lymph nodes is dependent upon the MAPK pathway. Arthritis Rheum. *54*, 765–778.

Saeed, A.I., Sharov, V., White, J., Li, J., Liang, W., Bhagabati, N., Braisted, J., Klapa, M., Currier, T., Thiagarajan, M., et al. (2003). TM4: a free, open-source system for microarray data management and analysis. Biotechniques *34*, 374–378.

Saraiva, M., Christensen, J.R., Tsytsykova, A.V., Goldfeld, A.E., Ley, S.C., Kioussis, D., and O'Garra, A. (2005). Identification of a macrophage-specific chromatin signature in the IL-10 locus. J. Immunol. *175*, 1041–1046.

Schneider, D., Manzan, M.A., Yoo, B.S., Crawford, R.B., and Kaminski, N. (2009). Involvement of Blimp-1 and AP-1 dysregulation in the 2,3,7,8-tetrachlorodibenzo-p-dioxin-mediated suppression of the IgM response by B cells. Toxicol. Sci. *108*, 377–388.

Shen, P., Roch, T., Lampropoulou, V., O'Connor, R.A., Stervbo, U., Hilgenberg, E., Ries, S., Dang, V.D., Jaimes, Y., Daridon, C., et al. (2014). IL-35-producing B cells are critical regulators of immunity during autoimmune and infectious diseases. Nature *507*, 366–370.

Shinde, R., Hezaveh, K., Halaby, M.J., Kloetgen, A., Chakravarthy, A., da Silva Medina, T., Deol, R., Manion, K.P., Baglaenko, Y., Eldh, M., et al. (2018). Apoptotic cell-induced AhR activity is required for immunological tolerance and suppression of systemic lupus erythematosus in mice and humans. Nat. Immunol. *19*, 571–582.

Stockinger, B., and Omenetti, S. (2017). The dichotomous nature of T helper 17 cells. Nat. Rev. Immunol. *17*, 535–544.

Stockinger, B., Di Meglio, P., Gialitakis, M., and Duarte, J.H. (2014). The aryl hydrocarbon receptor: multitasking in the immune system. Annu. Rev. Immunol. *32*, 403–432.

Szekanecz, Z., Halloran, M.M., Volin, M.V., Woods, J.M., Strieter, R.M., Kenneth Haines, G., 3rd, Kunkel, S.L., Burdick, M.D., and Koch, A.E. (2000). Temporal expression of inflammatory cytokines and chemokines in rat adjuvant-induced arthritis. Arthritis Rheum. *43*, 1266–1277.

Tarca, A.L., Draghici, S., Khatri, P., Hassan, S.S., Mittal, P., Kim, J.S., Kim, C.J., Kusanovic, J.P., and Romero, R. (2009). A novel signaling pathway impact analysis. Bioinformatics *25*, 75–82.

Thornton, S., Duwel, L.E., Boivin, G.P., Ma, Y., and Hirsch, R. (1999). Association of the course of collagen-induced arthritis with distinct patterns of cytokine and chemokine messenger RNA expression. Arthritis Rheum. *42*, 1109–1118.

Thornton, S., Boivin, G.P., Kim, K.N., Finkelman, F.D., and Hirsch, R. (2000). Heterogeneous effects of IL-2 on collagen-induced arthritis. J. Immunol. *165*, 1557–1563.

Tian, J., Zekzer, D., Hanssen, L., Lu, Y., Olcott, A., and Kaufman, D.L. (2001). Lipopolysaccharide-activated B cells down-regulate Th1 immunity and prevent autoimmune diabetes in nonobese diabetic mice. J. Immunol. *167*, 1081–1089.

Tucker, A.N., Vore, S.J., and Luster, M.I. (1986). Suppression of B cell differentiation by 2,3,7,8-tetrachlorodibenzo-p-dioxin. Mol. Pharmacol. *29*, 372–377.

Turner, C.A., Jr., Mack, D.H., and Davis, M.M. (1994). Blimp-1, a novel zinc finger-containing protein that can drive the maturation of B lymphocytes into immunoglobulin-secreting cells. Cell *77*, 297–306.

Vaidyanathan, B., Chaudhry, A., Yewdell, W.T., Angeletti, D., Yen, W.F., Wheatley, A.K., Bradfield, C.A., McDermott, A.B., Yewdell, J.W., Rudensky, A.Y., and Chaudhuri, J. (2017). The aryl hydrocarbon receptor controls cell-fate decisions in B cells. J. Exp. Med. *214*, 197–208.

Vasconcellos, R., Carter, N.A., Rosser, E.C., and Mauri, C. (2011). IL-12p35 subunit contributes to autoimmunity by limiting IL-27-driven regulatory responses. J. Immunol. *187*, 3402–3412.

Veldhoen, M., Hirota, K., Christensen, J., O'Garra, A., and Stockinger, B. (2009). Natural agonists for aryl hydrocarbon receptor in culture medium are essential for optimal differentiation of Th17 T cells. J. Exp. Med. *206*, 43–49.

Villa, M., Gialitakis, M., Tolaini, M., Ahlfors, H., Henderson, C.J., Wolf, C.R., Brink, R., and Stockinger, B. (2017). Aryl hydrocarbon receptor is required for optimal B-cell proliferation. EMBO J. *36*, 116–128.

Vos, J.G., Moore, J.A., and Zinkl, J.G. (1973). Effect of 2,3,7,8-tetrachlorodibenzo-p-dioxin on the immune system of laboratory animals. Environ. Health Perspect. *5*, 149–162.

Wagage, S., John, B., Krock, B.L., Hall, A.O., Randall, L.M., Karp, C.L., Simon, M.C., and Hunter, C.A. (2014). The aryl hydrocarbon receptor promotes IL-10 production by NK cells. J. Immunol. *192*, 1661–1670.

Yamaguchi, K., Matulka, R.A., Shneider, A.M., Toselli, P., Trombino, A.F., Yang, S., Hafer, L.J., Mann, K.K., Tao, X.J., Tilly, J.L., et al. (1997a). Induction of PreB cell apoptosis by 7,12-dimethylbenz[a]anthracene in long-term primary murine bone marrow cultures. Toxicol. Appl. Pharmacol. *147*, 190–203.

Yamaguchi, K., Near, R.I., Matulka, R.A., Shneider, A., Toselli, P., Trombino, A.F., and Sherr, D.H. (1997b). Activation of the aryl hydrocarbon receptor/transcription factor and bone marrow stromal cell-dependent preB cell apoptosis. J. Immunol. *158*, 2165–2173.

Yanaba, K., Bouaziz, J.D., Haas, K.M., Poe, J.C., Fujimoto, M., and Tedder, T.F. (2008). A regulatory B cell subset with a unique CD1dhiCD5+ phenotype controls T cell-dependent inflammatory responses. Immunity *28*, 639–650.

Yanaba, K., Bouaziz, J.D., Matsushita, T., Tsubata, T., and Tedder, T.F. (2009). The development and function of regulatory B cells expressing IL-10 (B10 cells) requires antigen receptor diversity and TLR signals. J. Immunol. *182*, 7459–7472.

Ye, J., Qiu, J., Bostick, J.W., Ueda, A., Schjerven, H., Li, S., Jobin, C., Chen, Z.E., and Zhou, L. (2017). The aryl hydrocarbon receptor preferentially marks and promotes gut regulatory T cells. Cell Rep. *21*, 2277–2290.

Yoshizaki, A., Miyagaki, T., DiLillo, D.J., Matsushita, T., Horikawa, M., Kountikov, E.I., Spolski, R., Poe, J.C., Leonard, W.J., and Tedder, T.F. (2012). Regulatory B cells control T-cell autoimmunity through IL-21-dependent cognate interactions. Nature *491*, 264–268.

Zhang, Y., Liu, T., Meyer, C.A., Eeckhoute, J., Johnson, D.S., Bernstein, B.E., Nusbaum, C., Myers, R.M., Brown, M., Li, W., and Liu, X.S. (2008). Model-based analysis of ChIP-Seq (MACS). Genome Biol. *9*, R137.

STAR★METHODS

KEY RESOURCES TABLE

REAGENT or RESOURCE	SOURCE	IDENTIFIER
Antibodies		
CD3e, Clone 145-2C11	BD PharMingen	Cat# 550275; RRID:AB_393572
*InVivo*Plus anti-mouse CD40	BioXcell	Cat# BP0016-2; RRID:AB_1107647
AffiniPure Fab Fragment Goat Anti-Mouse IgM, μ chain specific	Jackson ImmunoResearch	Cat# 115-007-020; RRID:AB_2338477
CD1d Percp/Cy5.5, Clone 1B1	Biolegend	Cat# 123514; RRID:AB_2073523
CD3 BV605, Clone 17A2	Biolegend	Cat# 100237; RRID:AB_2562039
CD4 BV605, Clone RM4-5	Biolegend	Cat# 100548; RRID:AB_2563054
CD4 BV711, Clone RM4-5	Biolegend	Cat# 100550; RRID:AB_2562099
CD5 AF647, Clone 53-7.3	Biolegend	Cat# 100614; RRID:AB_2075301
CD8a BV605, Clone 53-6.7	Biolegend	Cat# 100744; RRID:AB_2562609
CD11b BV605, Clone M1/70	Biolegend	Cat# 101257; RRID:AB_2565431
CD11c BV605, Clone N418	Biolegend	Cat# 117334; RRID:AB_2562415
CD19 BV785, Clone 6D5	Biolegend	Cat# 115543; RRID:AB_11218994
CD21 APC, Clone CR2/CR1	Biolegend	Cat# 123412; RRID:AB_2085160
CD23 FITC, Clone B3B4	Biolegend	Cat# 101606; RRID:AB_312831
CD24 PE-Cy7, Clone M1/69	Biolegend	Cat# 101822; RRID:AB_756048
CD24 BV421, Clone M1/69	Biolegend	Cat# 101826; RRID:AB_2563508
CD43 PECy7, Clone S7	BD PharMingen	Cat# 562866; RRID:AB_2737852
CD138 BV711, Clone 281-2	Biolegend	Cat# 142519; RRID:AB_2562571
F4/80 BV605, Clone BM8	Biolegend	Cat# 123133; RRID:AB_2562305
TER-119/Erythroid cells BV605, Clone TER-119	Biolegend	Cat# 116239; RRID:AB_2562447
Ly6C/G BV605, Clone RB6-8C5	Biolegend	Cat# 108440; RRID:AB_2563311
TCRβ BV605, Clone H57-597	Biolegend	Cat# 109241; RRID:AB_2629563
Tim-1 PE, Clone RMT1-4	Biolegend	Cat# 119506; RRID:AB_2232887
CD249 PE, Clone BP-1	BD PharMingen	Cat# 553735; RRID:AB_395018
Blimp-1 AF647, Clone 5E7	Biolegend	Cat# 150004; RRID:AB_2565618
Ki-67 BV421, Clone 16A8	Biolegend	Cat# 652411; RRID:AB_2562663
FoxP3 APC, Clone FJK-16	ThermoFisher Scientific	Cat# 17-5773-82; RRID:AB_469457
IFN-γ APC, Clone XMG1.2	ThermoFisher Scientific	Cat# 17-7311-82; RRID:AB_469504
IL-10 PE, Clone JES5-16E3	Biolegend	Cat# 505008; RRID:AB_315362
IL-17 PE (TC11-18H10.1)	Biolegend	Cat# 506904; RRID:AB_315464
AhR PE, Clone 4MEJJ	ThermoFisher Scientific	Cat# 12-5925-82; RRID:AB_2572644
Polyclonal antibody against AhR (used for ChIP and western blot)	Enzo Life Sciences	Cat# BML-SA210; RRID:AB_10540536
Rabbit anti-mouse β-actin, Clone 13E5	Cell Signaling Technology	Cat# 4970; RRID:AB_2223172
Goat Anti-Rabbit IgG – H&L Polyclonal antibody, HRP conjugated	Abcam	Cat# ab6721; RRID:AB_955447
Chemicals, Peptides, and Recombinant Proteins		
6-Formylindolo(3,2-b)carbazole (FICZ)	Enzo Life Sciences	Cat# BML-GR206
CH-223191 (AhR antagonist)	Merck Millipore	Cat# 182705
Lipopolysaccharide (LPS)	Sigma Aldrich	Cat# L4391
Methylated bovine serum albumin (mBSA)	Sigma Aldrich	Cat# A1009
Incomplete Freund's adjuvant (CFA)	Sigma Aldrich	Cat# F5506
CpG-B (ODN1826)	Invivogen	Cat# tlrl-1826

(*Continued on next page*)

Continued

REAGENT or RESOURCE	SOURCE	IDENTIFIER
Phorbol-12-myristate-13 acetate (PMA)	Sigma Aldrich	Cat# P8139
Ionomycin	Sigma Aldrich	Cat# I0634
DAPI	Sigma Aldrich	Cat# D9542
Brefeldin A	Biolegend	Cat# 420601
2-Mercaptoethanol	ThermoFisher Scientific	Cat# 31350010
RNase-Free DNase set	QIAGEN	Cat# 79254
Critical Commercial Assays		
IL-2 duoset ELISA kit	Biotechne	Cat# DY402
IL-6 duoset ELISA kit	Biotechne	Cat# DY402
IL-10 duoset ELISA kit	Biotechne	Cat# DY417
IL-17 duoset ELISA kit	Biotechne	Cat# DY421
TNFα duoset ELISA kit	Biotechne	Cat# DY410
Total mouse IgA ELISA kit	ThermoFisher Scientific	Cat# 88-50450-88
Total mouse IgG ELISA kit	ThermoFisher Scientific	Cat# 88-50470-88
Total mouse IgM ELISA kit	ThermoFisher Scientific	Cat# 88-50400-88
Negative CD43- Isolation Kit	Miltenyi Biotec	Cat# 130-049-801
Murine Treg isolation kit		N/A
Picopure^{TM} RNA isolation kit	ThermoFisher Scientific	Cat# KIT0204
iScript^{TM} cDNA synthesis kit	Biorad	Cat# 1708891
iQ^{TM} SYBR® green supermix	Biorad	Cat# 1708882
Nextera DNA library preparation kit	Illumina	Cat# FC-121-1030
MinElute PCR purification kit	QIAGEN	Cat# 28004
Pierce^{TM} BCA Protein Assay Kit	ThermoFisher Scientific	Cat# 23225
Deposited Data		
Raw microarray data – IL-10$^+$CD19$^+$CD21^{hi}CD24^{hi} B cells, IL-10$^-$CD19$^+$CD21^{hi}CD24^{hi} B cells, IL-10$^-$CD19$^+$CD21^{int}CD24^{int}	This paper	E-MTAB-7375
Raw and analyzed ATAC-seq data – IL-10$^+$CD19$^+$CD21^{hi}CD24^{hi} B cells, IL-10$^-$CD19$^+$CD21^{hi}CD24^{hi} B cells, IL-10$^-$CD19$^+$CD21^{int}CD24^{int}	This paper	E-MTAB-8393
Raw and analyzed ATAC-seq data – *Ahr*$^{fl/-}$*Mb1*$^{cre/+}$ and *Mb1*$^{cre/+}$ CD19$^+$CD21^{hi}CD24^{hi} B cells ex-vivo & after 6h LPS+anti-IgM stimulation	This paper	E-MTAB-7525
Raw and analyzed RNA-seq data - *Ahr*$^{fl/-}$*Mb1*$^{cre/+}$ and *Mb1*$^{cre/+}$ CD19$^+$CD21^{hi}CD24^{hi} B cells ex-vivo & after 6h LPS+anti-IgM stimulation	This paper	E-MTAB-7345
Experimental Models: Organisms/Strains		
Mouse, B6(Cg)-*Il10*$^{tm1.1Karp}$/J	Prof. Christopher Karp	RRID:IMSR_JAX:014530
Mouse, C57BL/6J	Envigo	N/A
Mouse, IL-10R KO	Pils et al., 2010. Professor Werner Muller	N/A
Mouse, *B6.C(Cg)-Cd79a*$^{tm1(cre)Reth}$*/EhobJ*	Jackson laboratory	RRID:IMSR_JAX:020505
Mouse, *B6.129-Ahr*^{tm1Bra}*/J*	Jackson laboratory	RRID:IMSR_JAX:002831
Mouse, *Ahr*$^{-/-}$*Mb1*$^{cre/cre}$	Prof. Brigitta Stockinger	N/A
Mouse, *Ahr*$^{fl/fl}$ *R26R FP635*$^{fl/fl}$	Prof. Brigitta Stockinger	N/A
Mouse, *Mb1*$^{cre/+}$	Generated in house from above strains	N/A
Mouse, *Ahr*$^{fl/-}$*Mb1*$^{cre/+}$	Generated in house from above strains	N/A
Oligonucleotides		
Please refer to Table S2	This paper	N/A

(*Continued on next page*)

Continued

REAGENT or RESOURCE	SOURCE	IDENTIFIER
Software and Algorithms		
GraphPad Prism 6	Graphpad Software	https://www.graphpad.com
Flowjo v10.5.0	Flowjo, LLC	https://www.flowjo.com
Limma	Ritchie et al., 2015	https://bioconductor.org/packages/release/bioc/html/limma.html
STAR	Dobin et al., 2013	https://github.com/alexdobin/STAR
HTSeq	Anders et al., 2015	https://htseq.readthedocs.io/en/release_0.11.1/install.html#install
Kallisto	Bray et al., 2016	https://pachterlab.github.io/kallisto/download
EdgeR	Robinson et al., 2010	https://bioconductor.org/packages/release/bioc/html/edgeR.html
Signaling pathway impact analysis	Tarca et al., 2009	https://bioconductor.org/packages/release/bioc/html/SPIA.html
Illumina Casava 1.7	Illumina	https://www.illumina.com
Picard Tools	N/A	https://broadinstitute.github.io/picard/
MACS2 v2.1.1.20160309	Zhang et al., 2008	https://github.com/taoliu/MACS
Multiple Experiment Viewer (MeV_4_8)	Saeed et al., 2003	http://mev.tm4.org/#/welcome
Other		
RPMI-1640 media	Sigma Aldrich	Cat# R8758
IMDM media	Lonza	Cat# BE12-915F
Red blood cell lysis buffer	Sigma Aldrich	Cat# R7757
Foetal calf serum (FCS)	Biosera	Cat# FB1001/500
Penicillin/Streptomycin	Sigma Aldrich	Cat# P0781
eBioscience^{TM} Intracellular fixation & permeabilisation buffer set	ThermoFisher Scientific	Cat# P078188-8824-00
Brilliant stain buffer	BD Biosciences	Cat# 563794
eBioscience^{TM} FoxP3 / Transcription Factor Staining Buffer Set	ThermoFisher Scientific	Cat# 00-5523-00
M. tuberculosis H37 Ra, desiccated	BD	Cat# 231141
Cell Lysis Buffer (10x)	Cell Signaling Technology	Cat# 9803
Pierce^{TM} ECL Western Blotting Substrate	ThermoFisher Scientific	Cat# 32106

LEAD CONTACT AND MATERIALS AVAILABILITY

Further information and requests for resources and reagents should be directed to and will be fulfilled by the Lead Contact, Professor Claudia Mauri (c.mauri@ucl.ac.uk). This study did not generate new unique reagents.

EXPERIMENTAL MODEL AND SUBJECT DETAILS

IL-10eGFP mice were as described (Madan et al., 2009), courtesy of Prof Karp. C57BL/6 mice were from purchased from Envigo, UK. *Ahr*$^{-/-}$, *Ahr*$^{+/-}$, *Mb1*$^{cre/+}$ (courtesy of Prof Reth) and *Ahr*$^{fl/-}$*Mb1*$^{cre/+}$ mice were kindly provided by Prof. Brigitta Stockinger. IL-10R$^{-/-}$ mice were kindly given courtesy of Professor Werner Muller. Male and female mice were used at 8–12 weeks of age and were bred and maintained at the animal facility, University College London. All experiments were approved by the Animal Welfare and Ethical Review Body of University College London and authorized by the United Kingdom Home Office.

Induction of antigen-induced arthritis (AIA)

AIA was induced and assessed as previously described (Rosser et al., 2014). Briefly, mice were injected subcutaneously at the tail base with 200 μg of methylated BSA (mBSA; Sigma-Aldrich) emulsified in 100 μL Complete Freund's Adjuvant (CFA). CFA was made by mixing 3mg/ml of *Mycobacterium tuberculosis* (Difco) in Incomplete Freund's Adjuvant (IFA; Sigma-Aldrich). After 7 days, mice received an intra-articular (IA) injection of 10 μL of PBS containing 200 μg mBSA in the right knee and 10 μL PBS alone in the left knee as a control. Joint size was measured using callipers (POCO 2T; Kroeplin GmbH) at daily intervals and swelling was calculated

as a percentage increase in size between the inflamed and control knee. All experiments, unless stated otherwise, were performed at day 7 post-IA injection.

METHOD DETAILS

Cell isolation and culture

For splenocyte and lymph node cell preparation, organs were mashed through a 70 μM cell strainer (BD Biosciences), as previously described (Rosser et al., 2014), and erythrocytes from spleens were lysed using Red Cell Lysis Buffer (Sigma-Aldrich). B cells were negatively purified by magnetic separation, according to manufacturer's instructions (Miltenyi Biotec). Cells were cultured with either RPMI 1640 (Sigma-Aldrich) containing L-glutamine and $NAHCO_3$ or Iscove's Modified Dulbecco's Medium (IMDM; Pan Biotech), enriched in AhR agonists (Veldhoen et al., 2009), supplemented with L-Glutamine and 25mM HEPES. Media were supplemented with 10% fetal calf serum (LabTech), 1% penicillin/streptomycin (100U/ml Penicillin+100 μg/ml streptomycin; Sigma-Aldrich) and 50 μM 2-Mercaptoethanol (ThermoFisher Scientific). Cells were cultured at 37°C with 5% CO_2.

Total lymphocytes, B cells and B cell subsets were cultured for 48h with CpGb ODN1826 (1 μM; Invivogen), LPS (1 μg/ml; Sigma-Aldrich) ± anti-mouse IgM (10 μg/ml; Jackson ImmunoResearch). or anti-CD40 (10 μg/ml; BioXcell). In addition, AhR agonist FICZ (100nM; Enzo LifeSciences) was added to culture. For 48h culture, anti-IgM ± FICZ were added 24h into culture.

ELISA's on cell culture supernatants and sera

Supernatants from cell cultures were harvested and analyzed for cytokines using standard sandwich IL-2, IL-6, IL-10, IL-17 and TNFα ELISA Kits (R&D Systems) and performed according to manufacturer's instructions. Serum was collected from $Mb1^{cre/+}$ and $Ahr^{fl/-}Mb1^{cre/+}$ day 7 post AIA and was analyzed for total IgA, IgG and IgM (ThermoFisher Scientific).

Flow cytometry and cell sorting

Flow cytometry was performed with the following directly conjugated antibodies from Biolegend: CD1d Percp/Cy5.5 (1B1), CD3 BV605 (17A2), CD4 BV711 (RM4-5), CD5 AF647 (53-7.3), CD19 BV785 (6D5), CD21 APC (CR2/CR1), CD23 FITC (B3B4), CD24 PE-Cy7 or BV421 (M1/69), CD43 PECy7 (S7), CD138 BV711 (281-2) and Tim-1 PE (RMT1-4). CD249 PE (BP-1) was purchased from BD Biosciences. For multi-color flow cytometric surface staining, cells were stained at 4°C for 20 min as previously described (Nistala et al., 2010). LIVE/DEAD fixable blue Dead Cell Stain (Life Technologies) was used to exclude dead cells from flow cytometric analysis. For measurement of intra-nuclear Blimp-1, Ki-67 and FoxP3 expression, cells were fixed for 25 minutes with FoxP3 Fixation buffer (ThermoFisher Scientific) and Blimp-1 AF647 (5E7), Ki-67 BV421 (16A8; Biolegend) or FoxP3 APC (FJK-16 s; ThermoFisher Scientific) was added in permeabilisation buffer. Intracellular cytokine analysis was performed as described previously (Rosser et al., 2014). Briefly, for detection of IFN-γ, IL-10 and IL-17, splenocyte or lymph node cells were cultured in complete medium with PMA (50ng/ml; Sigma-Aldrich), Ionomycin (250ng/ml; Sigma-Aldrich) and Brefeldin A (5 μg/ml; Sigma-Aldrich) for 4.5h. Cells were then stained with surface markers followed by intracellular fixation and permeabilisation (ThermoFisher Scientific). Cells were incubated with IFN-γ APC (XMG1.2; BD PharMingen), IL-10 PE (JES5-16E3; Biolegend) and IL-17 PE (TC11-18H10.1; BD PharMingen). FP635 and eGFP reporter expression were analyzed *ex vivo* without fixation.

B cell subsets were sorted using a cell sorter (FACSAria; BD PharMingen) by using CD19 BV785, CD21 APC, CD23 FITC and CD24 PE-Cy7. Dead cells were excluded by the use of 4,6-diamidino-2-phenylindole at 1 μg/ml (DAPI; Sigma). For cell sorting for RNA-seq and ATAC-seq, addition of BV605 dump channel antibodies against CD3 (17A2), CD4 (RM4-5), CD8a (53-6.7), CD11b (M1/70), CD11c (N418), F4/80 (BM8), Ly6C/G (RB6-8C5), erythroid cells (TER-119) and TCRβ (H57-597) were incorporated (Biolegend). Sort purity of B cell subpopulations was routinely > 95%. Flow cytometric data were collected on an LSRII or LSR Fortessa (BD PharMingen) using FACS Diva software. Data were analyzed using Flowjo (Tree Star).

In-vitro suppression assay

Splenic B cell subsets from $Ahr^{-/-}$ and $Ahr^{+/-}$ mice were sorted at day 7 post IA injection and stimulated with CpGb (ODN2006) for 6 hours. Cells were washed and then co-cultured with 0.5 μg/ml plate-bound anti-CD3 (145-2C11, BD Biosciences) for 72 hours with $CD4^+CD25^-$ (bead isolated) T cells from $Ahr^{+/-}$ mice. Following stimulation, cells were analyzed for $CD4^+$ IFN-γ expression. The percentage suppression of IFN-γ was calculated as a percentage reduction in IFN-γ from $CD4^+$ cells cultured alone, compared to when B cell subsets were added to culture.

Adoptive transfer experiments

$CD19^+CD21^{hi}CD24^{hi}$ B cells were isolated from spleens of $Mb1^{cre/+}$ and $Ahr^{fl/-}Mb1^{cre/+}$ mice after remission from AIA and $5x10^6$ were transferred into recipient wild-type mice on the day of intra-articular injection. The control group (no transfer) received a PBS injection.

Histology

For histopathological examination, joints from *Ahr*$^{fl/-}$*Mb1*$^{cre/+}$ and *Mb1*$^{cre/+}$ mice were removed post-mortem and fixed in 5% formalin and decalcified in 5% EDTA. The fixed joints were embedded in paraffin and 4 μm sections were cut and stained with hematoxylin-eosin.

Microarray analysis and RNA extraction

Splenic B cell subsets were sorted and RNA extracted using columns (Picopure, Life Technologies) and hybridized to murine mogene 2.0 ST arrays (Affymetrix). Raw CEL files were processed using the online GeneProfiler tool (accessible at https://www.beringresearch.com). Briefly, the GeneProfiler pipeline consists of present/absent call detection, (McClintick and Edenberg, 2006) Robust Microarray Average (RMA) normalization, and outlier detection (Kauffmann et al., 2009). Differential expression analysis was performed using the *limma* package (Ritchie et al., 2015).

Chromatin Immunoprecipitation

Total Vert-X splenic B cells were bead cell sorted and cultured for 24h with LPS, followed by addition of anti-IgM (10 μg/ml) and FICZ (100nM) at 24h into culture. After 48h total, total B cells were sorted based on eGFP for IL-10$^+$ and IL-10$^-$ populations. Cells were fixed for 10 minutes with 1% (vol/vol) formaldehyde and quenched with 400mM Tris. Fixed cells were lysed with 120 μL lysis buffer (1% (wt/vol) SDS, 10mM EDTA and 50mM Tris-HCl, pH 8.1, 1 × protease inhibitor ‘cocktail’ (Roche), 1mM PMSF) per 5x10^6 cells. Chromatin was sheared to 200-500bp fragments and 10% of the initial chromatin material was kept as input. The chromatin was diluted 5-fold in Dilution Buffer (1% (vol/vol) Triton X-100, 2mM EDTA, 150mM NaCl and 20mM Tris-HCl, pH 8.1) and incubated overnight, after pre-clearing, with 1 μg/10^6 cells of a polyclonal AhR-specific antibody (BML-SA210; Enzo Life Sciences). Immunoprecipitation took place by incubation with protein G Dynal magnetic beads (Invitrogen), held for at least 3 hours at 4°C. Immunoprecipitated chromatin complexes were washed with High Salt Wash Buffer (2x), Low Salt Wash Buffer (2x), LiCl Wash Buffer (2x) and TE Buffer (2x). Immunoprecipitated chromatin was eluted from the magnetic beads with Proteinase K Digestion Buffer and heated at 65°C for at least 6h for reverse crosslinking. DNA fragments were purified with NucleoMag beads kit (MN) and were analyzed by SYBR Green Quantitative Real-time PCR. The following primers were used for ChIP qPCR: *Il10* −3.5kb forward (5′-AGGGCTTGATAACGTGTGAGT-3′); *Il10* −3.5kb reverse (5′-TGAACTTCACACCCAGCTTGAG-3′); *Il10* −2kb forward (5′-TAAGAGGTGCTGCTTCTCCTG-3′); *Il10* −2kb reverse (5′-TGGCACTGGACAGTTCTATGA-3′); *Il10* −0.5kb forward (5′-AGGGAGGAGGAGCCTGAATAA-3′); *Il10* −0.5kb reverse (5′-CCTGTTCTTGGTCCCCCTTTT-3′); *Il10* +2kb forward (5′-GCCACATGCATCCAGAGACAC-3′); *Il10* +2kb reverse (5′-GTGCCTCAAAGTCACTCCCAC-3′); *Cyp1a1* −3.6kb forward (5′-GCTCTTTCTCTGCCAGGTTG-3′); *Cyp1a1* −3.6kb reverse (5′-GGCTAAGGGTCACAATGGAA-3′); *Cyp1a1* promoter forward (5′-AAGCATCACCCTTTGTAGCC-3′); *Cyp1a1* promoter reverse (5′-CAGGCAACACAGAGAAGTCG-3′); *Gapdh* promoter forward (5′-GCGCGAAAGTAAAGAAAGAAGCCC-3′); *Gapdh* promoter reverse (5′-AGCGGCCCGGAGTCTTAAGTATTAG-3′).

Western blot

5x10^6 cells CD19$^+$CD21^{hi}CD24^{hi} and FO B cells were FACS sorted from arthritic WT mice and lysed for 15 minutes at 4°C with cell lysis buffer (Cell signaling technology) for extraction of whole cell lysate. Protein was resolved by SDS-PAGE, transferred to nitrocellulose membranes and blotted using anti-AhR at 1/1000 (Enzo Life sciences) and anti-β-actin at 1/1000 (Cell Signaling Technology). Bound antibodies were revealed with a goat-anti rabbit H&L HRP-conjugated secondary antibody (1/1000) and ECL western blotting substrate (ThermoFisher Scientific).

RNA-seq

Splenic CD19$^+$CD21^{hi}CD24^{hi} B cells were isolated from *Mb1*^{cre} and *Ahr*$^{fl/-}$*Mb1*^{cre} mice in the remission phase of arthritis, at day 7 post-IA injection. Sorted cells were either left untouched (*ex-vivo*) or stimulated with LPS+anti-IgM for 6h in IMDM media. Dead cells were excluded using DAPI. Total RNA was isolated from these populations using the Picopure RNA isolation kit (ThermoFisher Scientific), according to manufacturer’s instructions. 60bp single reads were sequenced on 3 lanes of an Illumina hiseq. 130-500ng of total RNA was fragmented followed by reverse transcription and second strand cDNA synthesis. The double strand cDNA was subjected to end repair, A base addition, adaptor ligation and PCR amplification to create libraries. Libraries were evaluated by Qubit and TapeStation. Sequencing libraries were constructed with barcodes to allow multiplexing of samples in 3 lanes. Around 23-43 million single-end 60-bp reads were sequenced per sample on an Illumina HiSeq 2500 V4 instrument.

Poly-A/T stretches and Illumina adapters were trimmed from the reads using cutadapt. Resulting reads < 30bp were discarded. Reads were mapped to the *Mus musculus* GRCm38 reference genome using STAR (Dobin et al., 2013). Gene annotations were applied from Ensembl (EndToEnd option and outFilterMismatchNoverLmax was set to 0.04). Gene expression levels were quantified using htseq-count (“HTSeq,” n.d.) (Anders et al., 2015), using the gtf above. Transcripts per million (TPM) values were estimated independently using Kallisto (Bray et al., 2016).

Bioinformatic analysis of RNA-seq data

Differential expression analysis was performed using the default settings of the edgeR algorithm (Robinson et al., 2010). p values reflect two-sided p values obtained using the exact test proposed by Robinson and Smyth (Robinson and Smyth, 2008) for a

difference in means, between two groups of negative binomial random variables (implemented in edgeR package). Signaling Pathway Impact Analysis (SPIA) (Tarca et al., 2009) was used to detect significantly over-represented pathways, with the Kyoto Encyclopedia of Genes and Genomes (KEGG) Pathways database (Kanehisa et al., 2016) employed as a reference. The full mouse genome was used as background for enrichment.

ATAC-seq

ATAC-seq was performed on splenic IL-10eGFP^{+}CD19^{+}CD21^{hi}CD23^{hi}CD24^{hi}, IL-10eGFP^{-}CD19^{+}CD21^{hi}CD23^{hi}CD24^{hi} and IL-10eGFP^{-}FO B cells. In addition, ATAC-seq was performed on splenic total CD19^{+}CD21^{hi}CD24^{hi} B cells isolated as above for the RNA-seq from *Mb1*$^{cre/+}$ and *Ahr*$^{fl/-}$*Mb1*$^{cre/+}$ mice either left untouched (ex-vivo) or stimulated with LPS+anti-IgM for 6h in IMDM media. After sorting, 40,000 were washed with 1xPBS (10% FCS). The cell pellet was prepped for sequencing by using the Nextera DNA library preparation kit (Illumina). Briefly, 10.5 μL nuclease free water, 12.5 μL 2x Transposase buffer, 2 μL transposase and 0.25 μL digitonin (0.05%) per reaction were added to the cell pellets. Cells were incubated at 37°C for 30 minutes. DNA was then purified using a MinElute PCR purification kit (QIAGEN), according to manufacturer's instructions. Following DNA purification, 1μl of eluted DNA was used in a qPCR reaction to estimate the optimum number of amplification cycles. Library amplification was performed using custom Nextera primers and was followed by solid phase reversible immobilization (SPRI) size selection to exclude fragments larger than 1,200bp. DNA concentration was measured with a Qubit fluorometer (Life Technologies). The libraries were sequenced by the Biomedical Sequencing Facility at CeMM using the Illumina HiSeq4000 platform and the 50bp single-end configuration.

Bioinformatic analysis of ATAC-seq data

Bioinformatic analysis was performed as previously described (Rendeiro et al., 2016). Briefly, Illumina Casava1.7 software was used for basecalling. Sequenced reads were trimmed for adaptor and Nextera sequences and reads were mapped to mm10 reference genome using bowtie2 v2.2.4 with the "–very-sensitive" parameter. Duplicate reads were marked and removed with picard tools version 1.118. Reads were extended to the average fragment size and bigWig files containing counts of reads per basepair created. Peaks for ATAC-seq samples were called with MACS2 version 2.1.1.20160309 (Zhang et al., 2008) using the "–nomodel" and "–extsize 147" parameters. Peaks were assigned to genes by proximity. If a peak overlapped the gene body or promoter ± 2500bp of the transcription start site (TSS), the peak was assigned to that gene. If a peak did not fall into these criteria, the peak was assigned to the closest TSS. If the nearest TSS to the peak was further than 100kb away, no gene was assigned.

qPCR

qPCR analyses were performed as described (Nistala et al., 2010). RNA from isolated B cells/subsets was extracted using Arcturus Picopure RNA isolation kit (ThermoFisher Scientific) and RNA was reverse transcribed using an iScript cDNA synthesis kit (Bio-Rad), according to manufacturer's instructions. qPCR was performed on the cDNA samples using iQ SYBR® Green Supermix (Bio-Rad), according to manufacturer's instructions. Primers were used at a concentration of 10 μM. Quantitect primers for *Arnt*, *Ahrr* and *Cyp1a1* were purchased from QIAGEN. Primers for *β-Actin* and *Ahr* were custom designed with the following sequences: *Act* Forward (5′-AGATGACCCAGATCATGTTTGAG-3′); *Act* Reverse (5′- AGGTCCAGACGCAGGATG-3′); *Ahr* Forward (5′-AGGATCGGGG TACCAGTTCA-3′); *Ahr* Reverse (5′-CTCCAGCGACTGTGTTTTGC-3′); *Il6* Forward (5′-GCCTTCTTGGGACTGATGCT-3′); *Il6* Reverse (5′-TGCCATTGCACAACTCTTTTC-3′); *Il5ra* Forward (5′-GGTCCCGGTATGCAGTTCTA-3′); *Il5ra* Reverse 5′-AGCCGAATGCTG GAAAAGTG-3′. *Ccl22* (Hao et al., 2016), *Ebi3* (Shen et al., 2014), *Il2* (Martins et al., 2008), *Il10* (Yanaba et al., 2009), *p35* (Shen et al., 2014) *Tnf* (Denaës et al., 2016) were used as previously described. qPCR data were calculated as the ratio of gene to β-Actin expression by the relative quantification method ($\Delta\Delta C_t$; means ± s.e.m. of triplicate determination).

QUANTIFICATION AND STATISTICAL ANALYSIS

Heatmap analyses for microarray, RNA-seq and ATAC-seq datasets were performed using Multiple Experiment Viewer (MeV_4_8) software (Saeed et al., 2003). Hierarchical clustering was applied to genes using average linking clustering with the Euclidean distance metric. All data are expressed as mean ± s.e.m, unless stated otherwise. For *in vivo* studies, power calculations were performed on data showing mean maximum wild-type arthritic knee swelling of 2 mm with a s.d. of 0.39 mm, and an expected test group (transferred T2-MZPs) arthritic knee swelling of 1.4 mm. Group sizes of three mice or above were sufficient to reach a statistical power of at least 80% (http://www.statisticalsolutions.net/pss_calc.php). Mice were assigned at random to treatment groups for all mouse studies and, where possible, mixed among cages. Clinical scoring was performed in a blinded fashion. Mice that developed adverse reactions to protocols were excluded from datasets. Statistical significance was determined using unpaired t tests (comparison of two groups), using mann-whitney tests (comparison of two groups, non-parametric data), one-way ANOVA (comparison of three or more groups) or two-way ANOVA (comparison of two or more groups with 2 independent variables). One and two-way ANOVA were assessed with Bonferonni's multiple comparison tests. All data met the assumption of

statistical tests and had a normal distribution and variance was similar between groups that were statistically compared. Results were considered significant at $p \leq 0.05$. Statistical tests were performed using GraphPad Prism (La Jolla, CA, USA) v.6, Software for Apple Mac.

DATA AND CODE AVAILABILITY

The microarray, RNA-seq and ATAC-seq datasets generated during this study are available at ArrayExpress: E-MTAB-7345, E-MTAB-7375, E-MTAB-7525 and E-MTAB-8393.

Cell Reports, Volume *29*

Supplemental Information

Aryl Hydrocarbon Receptor Contributes to the Transcriptional Program of IL-10-Producing Regulatory B Cells

Christopher J.M. Piper, Elizabeth C. Rosser, Kristine Oleinika, Kiran Nistala, Thomas Krausgruber, André F. Rendeiro, Aggelos Banos, Ignat Drozdov, Matteo Villa, Scott Thomson, Georgina Xanthou, Christoph Bock, Brigitta Stockinger, and Claudia Mauri

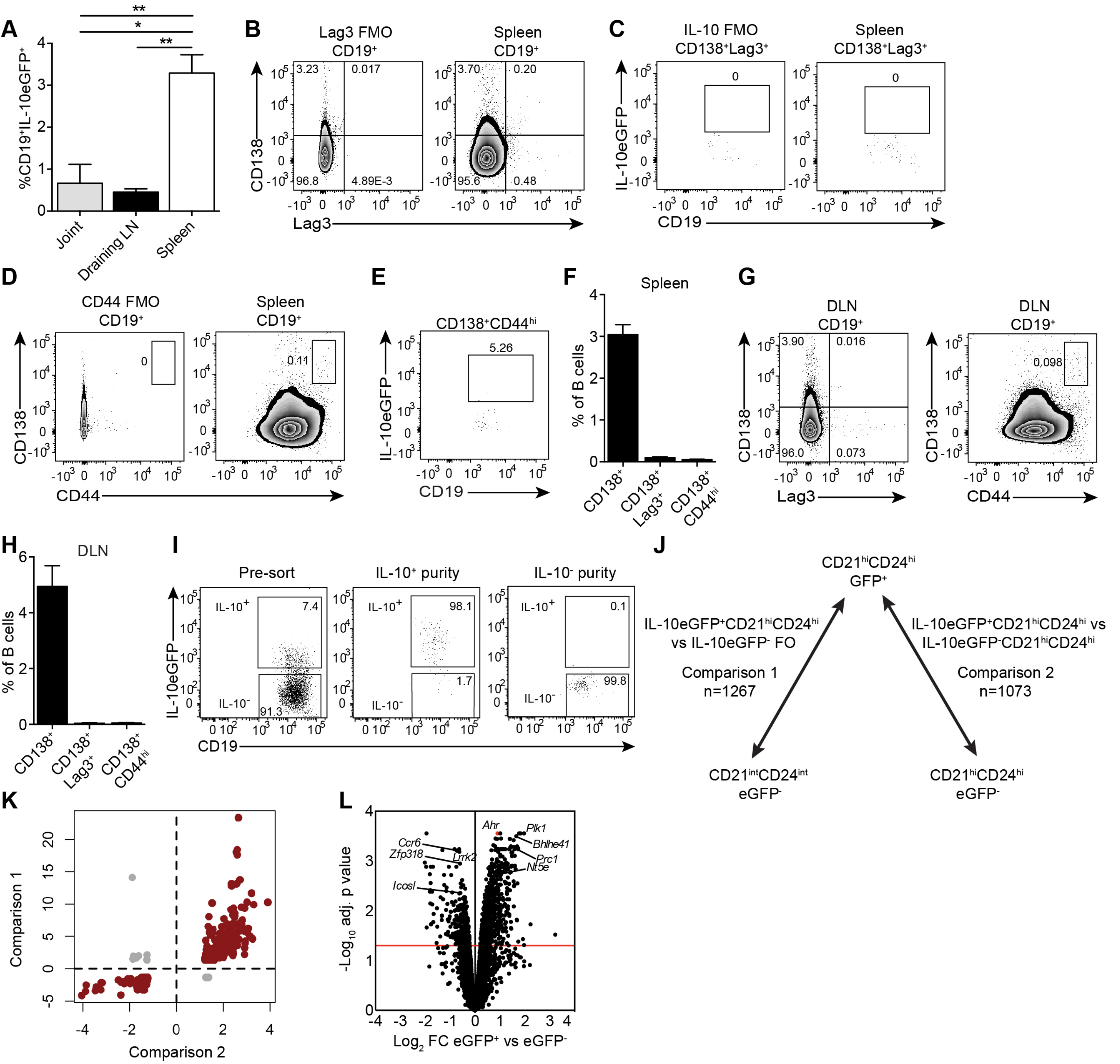

Figure S1. Comparison of gene expression profiles between eGFP^{+} and eGFP^{-} subsets. Related to Figures 1 and 2. Antigen-induced arthritis (AIA) was induced in IL-10eGFP reporter (Vert-X) mice. (**A**) Bar chart showing the frequencies of IL-10^{+}CD19^{+} B cells in the joint, draining LN and spleens of Vert-X mice (n=3). **B-E,** Representative flow cytometry plots showing respectively the frequencies of (**B**) CD138^{+}Lag3^{+} plasmablasts, (**C**) IL-10^{+} CD138^{+}Lag3^{+}, (**D**) CD138^{+}CD44^{hi} plasmablasts and (**E**) IL-10^{+}CD138^{+}CD44^{hi} B plasmablasts in the spleen. (**F**) Bar chart showing the percentages of CD19^{+}CD138^{+}, CD19^{+}CD138^{+}Lag3^{+} and CD19^{+}CD138^{+}CD44^{hi} plasmablasts in the spleens of in Vert-X mice, as shown gated in **B+D** (n=5). (**G**) Representative flow cytometry plots showing respectively the frequencies of (left) CD138^{+}Lag3^{+} and (right) CD138^{+}CD44^{hi} plasmablasts in the DLNs of Vert-X mice. (**H**) Bar chart showing the percentages of CD19^{+}CD138^{+}, CD19^{+}CD138^{+}Lag3^{+} and CD19^{+}CD138^{+}CD44^{hi} plasmablasts in the DLNs of in Vert-X mice, as shown gated in **G** (n=5). (**I**) Representative flow cytometry plots showing purity of CD19^{+}CD21^{hi}CD24^{hi}eGFP^{+} and CD19^{+}CD21^{hi}CD24^{hi}eGFP^{-} B cells. (**J**) Total number of differentially expressed genes between CD19^{+}CD21^{hi}CD24^{hi}GFP^{+} and IL-10eGFP^{-} subsets (>1.5 fold change, adjusted p value <0.05). (**K**) Scatter plot showing fold changes of differentially expressed genes from the two comparisons (n=660). Concordant changes for both comparisons are shown in red, and discordant in grey. (**L**) Volcano plot analysis showing $\log_2$ fold changes (FC) between CD19^{+}CD21^{hi}CD24^{hi}eGFP^{+} B cells versus CD19^{+}CD21^{hi}CD24^{hi}eGFP^{-} B cells, plotted against $-\log_{10}$ adjusted p value. *Ahr* is highlighted in red (adjusted p value of 3.4E-05). All experiments were carried out at day 7 post IA-injection. For figures **A-H,** data representative of 2 independent experiments. Figures **A, F and H,** data are expressed as mean±sem. *p<0.05, **p<0.01, one-way ANOVA.

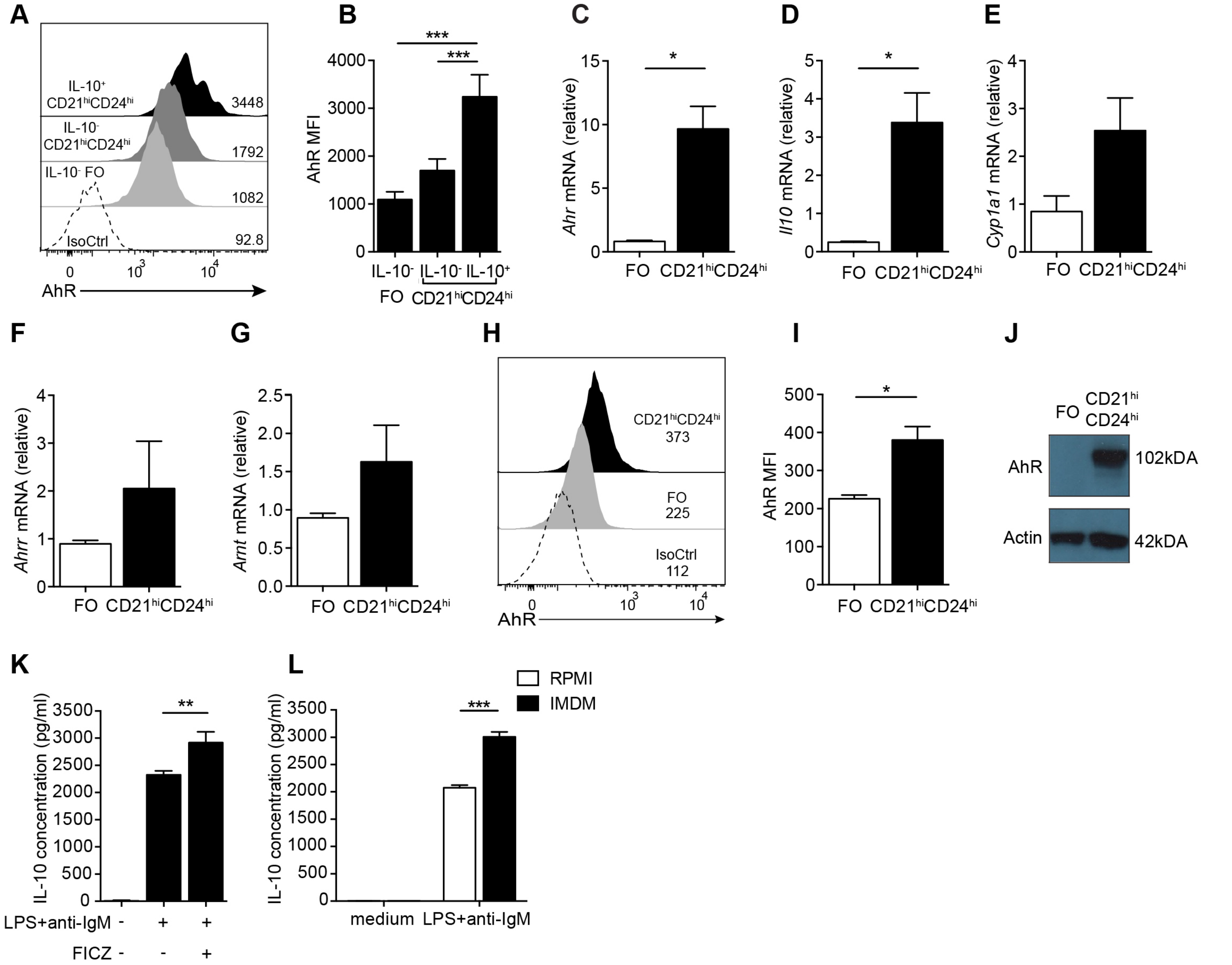

Figure S2. AhR is upregulated in IL-10^{+}CD19^{+}CD21^{hi}CD24^{hi}B cells after stimulation with LPS+anti-IgM. Related to Figure 2 and Figure 3. (**A**) Representative histogram and (**B**) bar chart showing the MFI of AhR expression in IL-10^{+}CD19^{+}CD21^{hi}CD24^{hi}, IL-10^{-}CD19^{+}CD21^{hi}CD24^{hi} and IL-10^{-}FO B cells after 48h stimulation with LPS+anti-IgM (*n*=4). **C-J, Increased levels of *Ahr* and down-stream pathway in *ex vivo* CD19^{+}CD21^{hi}CD24^{hi} compared to FO B cells.** CD19^{+}CD21^{hi}CD24^{hi} and FO B cells were isolated from WT mice and the mRNA levels of (**C**) *Ahr*, (**D**) *Il10*, (**E**) *Cyp1a1*, (**F**) *Ahrr* and (**G**) *Arnt* were analysed *ex-vivo* (*n*=3). (**H**) Representative histogram and (**I**) bar chart showing the median fluorescent intensity (MFI) of AhR expression in CD19^{+}CD21^{hi}CD24^{hi} and FO B cells *ex vivo* (*n*=4). (**J**) Western blot showing the expression of AhR in CD19^{+}CD21^{hi}CD24^{hi} and FO B cells isolated from arthritic WT mice. β-actin was used as a loading control. The numbers indicate the size of the protein bands in kDA. **K-L, AhR agonists increase IL-10 concentration in LPS+anti-IgM stimulated CD19^{+}CD21^{hi}CD24^{hi}B cells.** (**K**) CD19^{+}CD21^{hi}CD24^{hi}B cells were cultured in RPMI media for 48h with LPS+anti-IgM±FICZ and IL-10 was measured in the supernatant (*n*=4). (**L**) CD19^{+}CD21^{hi}CD24^{hi}B cells were cultured in LPS+anti-IgM for 48h in RPMI or IMDM media and IL-10 was measured in the supernatants (*n*=5). For qPCR, gene expression was calculated normalizing to β-Actin. All experiments were carried out at day 7 post IA-injection. Data representative of at least 2 independent experiments with biological replicates. Figures **B-G, I and K-L** data are expressed as mean±sem. *p<0.05, **p<0.01, ***p<0.001, one and two-way ANOVA and unpaired t test.

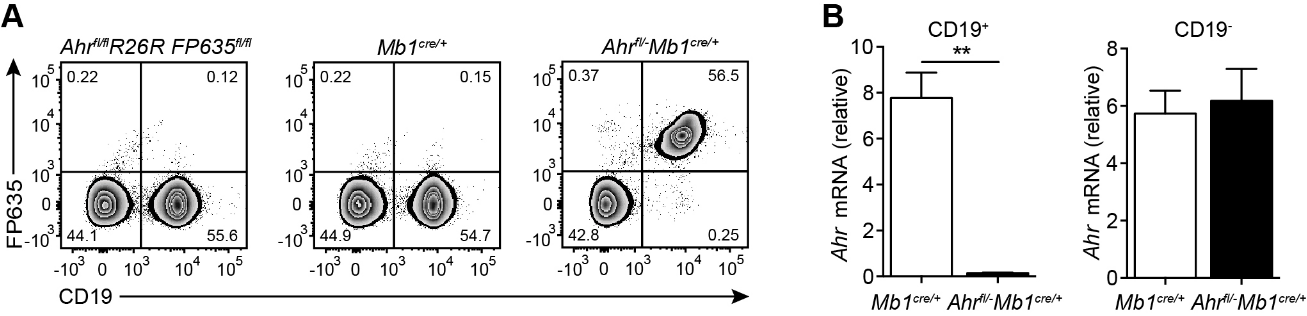

Figure S3. Validation of B cell AhR deficient (*Ahr*$^{fl/-}$*Mb1*$^{cre/+}$) mice. Related to Figures 4-6. (**A**) *Ahr*$^{fl/-}$*Mb1*$^{cre/+}$ mice lack *Ahr* in Mb1-expressing cells and report Cre activity via FP635 expression. Representative flow cytometry plots of FP635 expression in the parental *Ahr*$^{fl/fl}$*R26R FP635*$^{fl/fl}$ strain, *Mb1*$^{cre/+}$ control mice and *Ahr*$^{fl/-}$*Mb1*$^{cre/+}$ mice. (**B**) Splenocytes from *Ahr*$^{fl/-}$*Mb1*$^{cre/+}$ mice and *Mb1*$^{cre/+}$ controls were sorted into CD19^{+}B220^{+} and CD19^{-}B220^{-} fractions and the levels of *Ahr* mRNA were analysed *ex-vivo* (*n*=3). For qPCR, gene expression was calculated normalizing to β-Actin. All experiments were carried out at day 7 post IA-injection. Data representative of at least 2 independent experiments with biological replicates. Figure **B,** data are expressed as mean±sem. ***p*<0.01, unpaired t test.

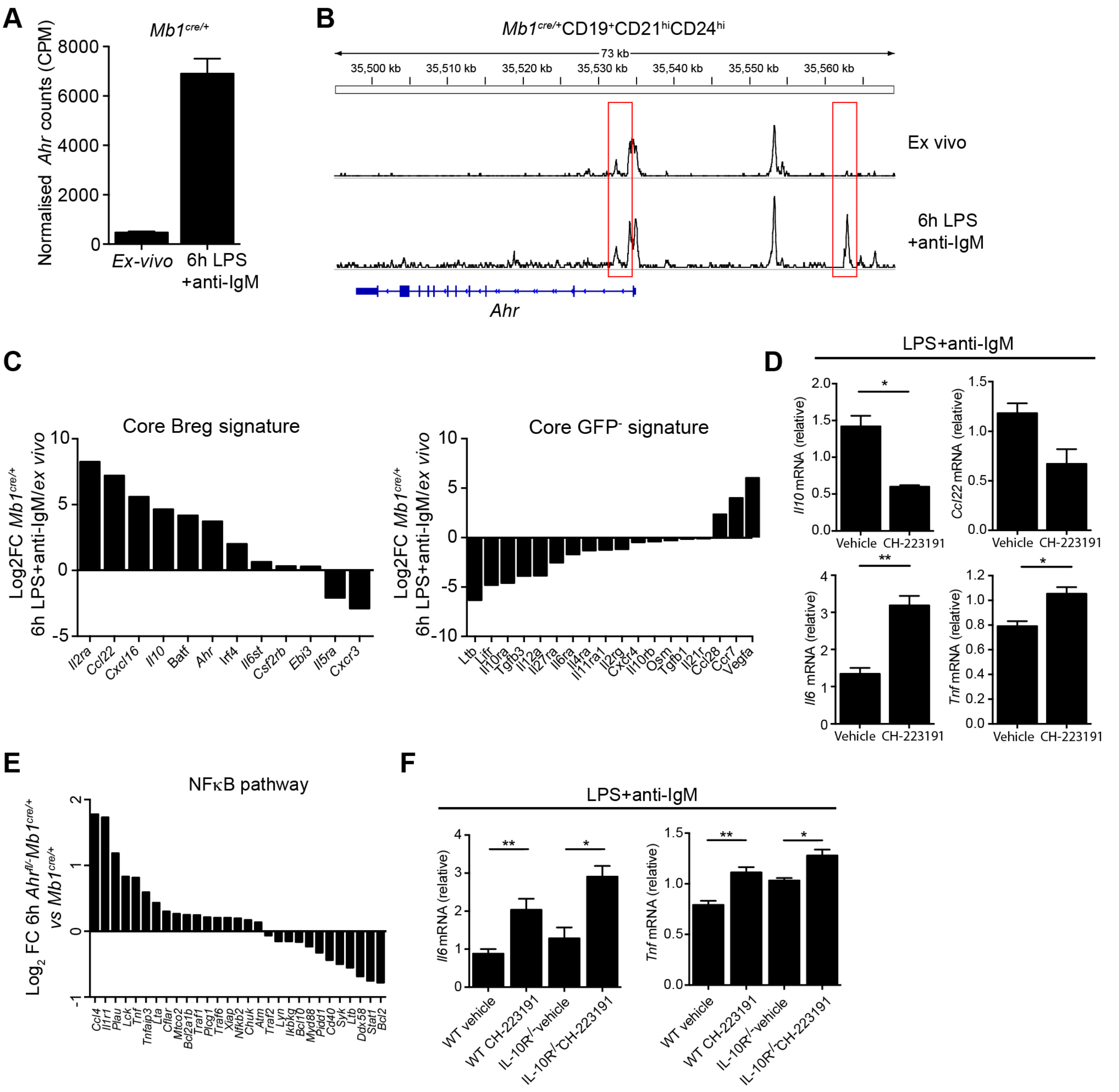

Figure S4. AhR contributes to the chromatin and transcriptional landscape of CD19^{+}CD21^{hi}CD24^{hi}B cells after Breg priming conditions. Related to Figure 4. (**A**) Normalised counts (CPM) of *Ahr* expression in *Mb1*$^{cre/+}$ mice *ex vivo* and after activation for 6h with LPS+anti-IgM. (**B**) Representative track of the *Ahr* locus before and after stimulation with LPS+anti-IgM in *Mb1*$^{cre/+}$ CD19^{+}CD21^{hi}CD24^{hi} B cells. Red box indicates one significantly differentially accessable region. (**C**) Log$_2$ FC for core GFP^{+} and GFP^{-} gene signatures (identified from Figure 1G) comparing 6h LPS+anti-IgM vs *ex vivo Mb1*$^{cre/+}$ CD19^{+}CD21^{hi}CD24^{hi}B cells. (**D**) CD19^{+}CD21^{hi}CD24^{hi}B cells were isolated from WT mice and stimulated for 24h with LPS+anti-IgM in the presence of the AhR antagonist (CH-223191) or a vehicle control and *Il10, Ccl22, Il6* and *Tnf* mRNA levels were analyzed (*n*=5). (**E**) Log$_2$ FC for NF-κB pathway genes (taken from KEGG) comparing 6h LPS+anti-IgM stimulated CD19^{+}CD21^{hi}CD24^{hi}B cells from *Mb1*$^{cre/+}$ and *Ahr*$^{fl/-}$*Mb1*$^{cre/+}$ mice. (**F**) WT or IL-10R$^{-/-}$ CD19^{+}CD21^{hi}CD24^{hi}B cells were cultured with LPS+anti-IgM±CH-223191 and *Il6* and *Tnf* mRNA levels were analyzed (*n*=5). For qPCR, gene expression was calculated normalizing to β-Actin. All experiments were carried out at day 7 post IA-injection. For RNA-seq data, *n*=3 per condition and genotype. For ATAC-seq data, *n*=3 for *Mb1*$^{cre/+}$ mice and *n*=2 for *Ahr*$^{fl/-}$*Mb1*$^{cre/+}$ mice. Figures **A, D and F,** data expressed as mean±sem. *p<0.05, **p<0.01, Mann-whitney test and two-way ANOVA. Figures **D** and **F,** representative of two independent experiments with biological replicates.

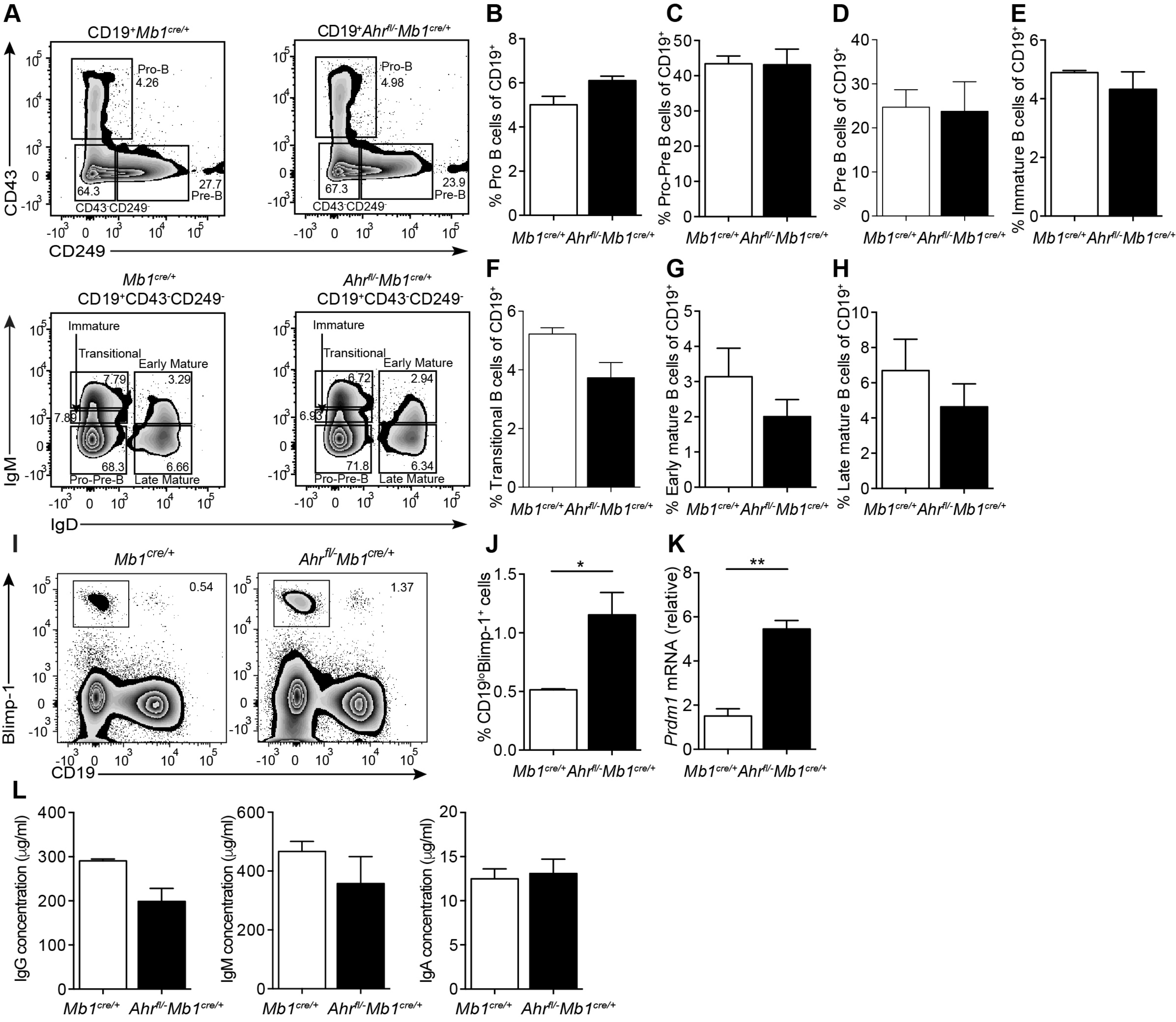

Figure S5. A-H, AhR plays a redundant role in early B cell development in the bone marrow. Related to Figure 6. (**A**) Representative flow cytometry plots showing *Mb1*$^{cre/+}$ and *Ahr*$^{fl/-}$*Mb1*$^{cre/+}$ B cell subsets in the bone marrow. **B-H,** Bar charts showing the frequencies of (**B**) pro, (**C**) pro-pre, (**D**) pre, (**E**) immature, (**F**) transitional (**G**) early mature and (**H**) late mature B cells, as a percentage of total CD19^{+} B cells in the bone marrow for *Mb1*$^{cre/+}$ and *Ahr*$^{fl/-}$*Mb1*$^{cre/+}$ mice (*n*=3 per genotype). **I-K, AhR represses plasma cell differentiation.** (**I**) Representative flow cytometry plots and (**J**) bar chart showing the percentage of splenic Blimp-1^{+} B cells from *Mb1*$^{cre/+}$ and *Ahr*$^{fl/-}$*Mb1*$^{cre/+}$ mice (*n*=3). (**K**) Total splenic B cells were isolated from *Mb1*$^{cre/+}$ and *Ahr*$^{fl/-}$*Mb1*$^{cre/+}$ mice and *Prdm1* mRNA levels were analysed *ex-vivo* (*n*=3). (**L**) Serum concentrations of total IgG, IgM and IgA from *Mb1*$^{cre/+}$ and *Ahr*$^{fl/-}$*Mb1*$^{cre/+}$ mice were measured by ELISA. For qPCR, gene expression was calculated normalizing to β-Actin. All experiments were carried out at day 7 post IA-injection. Data representative of at least 2 independent experiments with biological replicates. Figures **B-H, and J-L** data are expressed as mean±sem. *p<0.05, **p<0.01, unpaired t test.

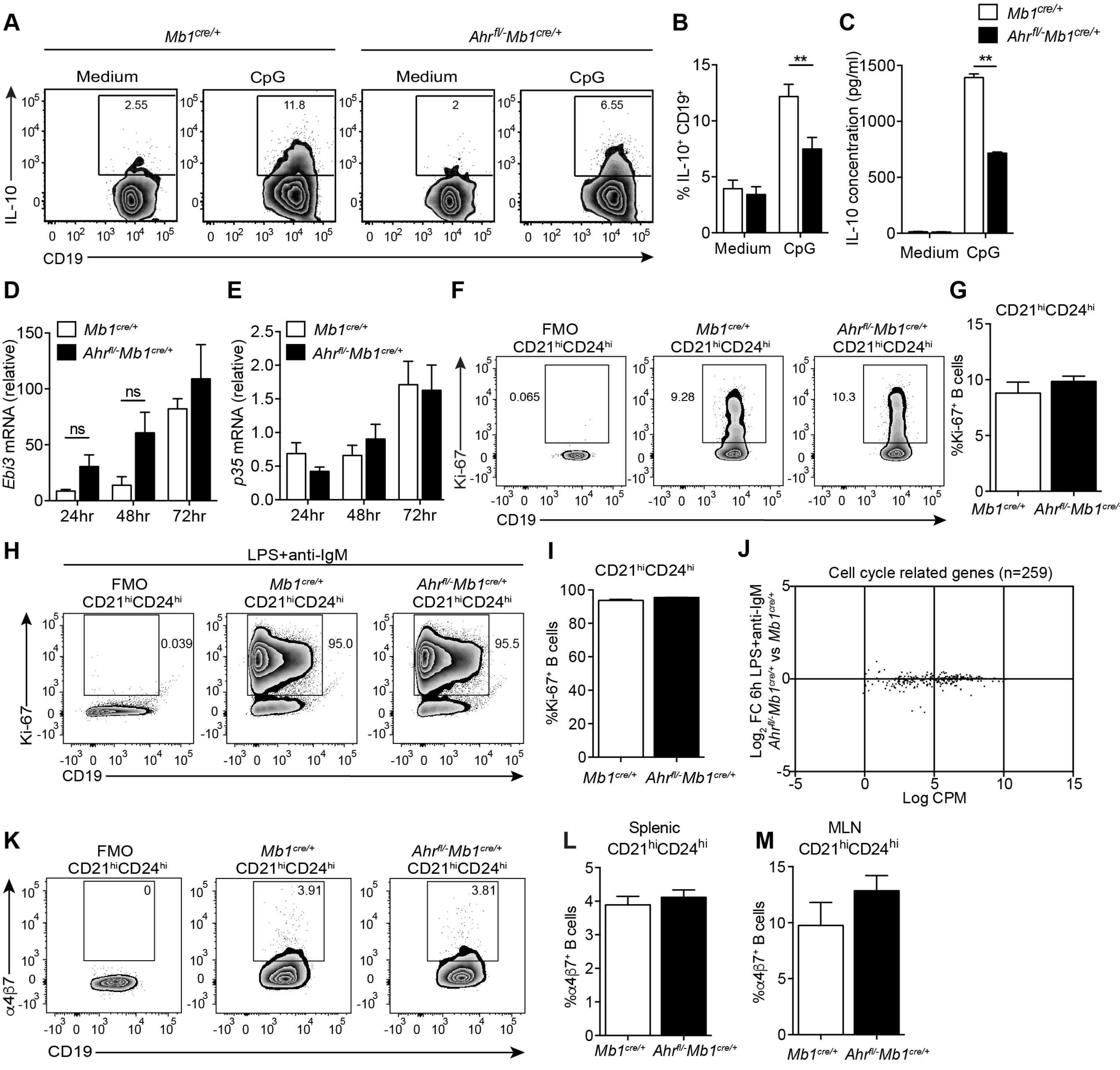

Figure S6. A-C, AhR is required for IL-10 production by Breg *in vitro*. Related to Figure 6. Representative flow cytometry plots (**A**) and bar chart (**B**) showing the percentage of IL-10-expressing CD19^{+}B cells from *Mb1*$^{cre/+}$ and *Ahr*$^{fl/-}$*Mb1*$^{cre/+}$ mice, after 48h stimulation with CpGb (*n*=3). (**C**) IL-10 production, as measured by ELISA (*n*=3). **D-E, AhR does not control IL-35 production by B cells.** Splenic B cells were isolated from *Mb1*$^{cre/+}$ and *Ahr*$^{fl/-}$*Mb1*$^{cre/+}$ mice and stimulated with LPS for the indicated times and (**D**) *Ebi3* and (**E**) *p35* mRNA levels were analysed (*n*=3). **F-J, AhR does not affect the proliferation of CD19^{+}CD21^{hi}CD24^{hi}B cells in arthritic mice.** (**F**) Representative flow cytometry plots and (**G**) bar graphs summarising Ki-67 expression in CD19^{+}CD21^{hi}CD24^{hi}B cells from *Mb1*$^{cre/+}$ and *Ahr*$^{fl/-}$*Mb1*$^{cre/+}$ mice *ex vivo* after day 7 AIA and (**H-I**) after 48h stimulation with LPS+anti-IgM (*n*=3). (**J**) Volcano plot (RNA-seq analysis) showing log$_{2}$ fold changes (FC) between 6h LPS+anti-IgM stimulated CD19^{+}CD21^{hi}CD24^{hi}B cells from *Ahr*$^{fl/-}$*Mb1*$^{cre/+}$ versus *Mb1*$^{cre/+}$ mice, plotted against average log counts per million (CPM; across all samples) for cell cyle related genes (n=259). **K-M, α4β7 is not differentially expressed between *Mb1*$^{cre/+}$ and *Ahr*$^{fl/-}$*Mb1*$^{cre/+}$ CD19^{+}CD21^{hi}CD24^{hi}B cells.** (**K**) Representative flow cytometry plots of splenic α4β7 expression in CD19^{+}CD21^{hi}CD24^{hi}B cells from *Mb1*$^{cre/+}$ and *Ahr*$^{fl/-}$*Mb1*$^{cre/+}$ mice. **L-M,** Bar charts showing the frequencies of α4β7-expressing CD19^{+}CD21^{hi}CD24^{hi}B cells in the (**L**) spleen and (**M**) MLNs of *Mb1*$^{cre/+}$ and *Ahr*$^{fl/-}$*Mb1*$^{cre/+}$ mice (*n*=6). All experiments were carried out at day 7 post IA-injection. For qPCR gene expression was calculated normalising to β-Actin. All data representative of at least 2 independent experiments, with biological replicates. Figures **B-E, G, I and L-M,** data expressed as mean±sem. **p<0.01, two-way ANOVA.

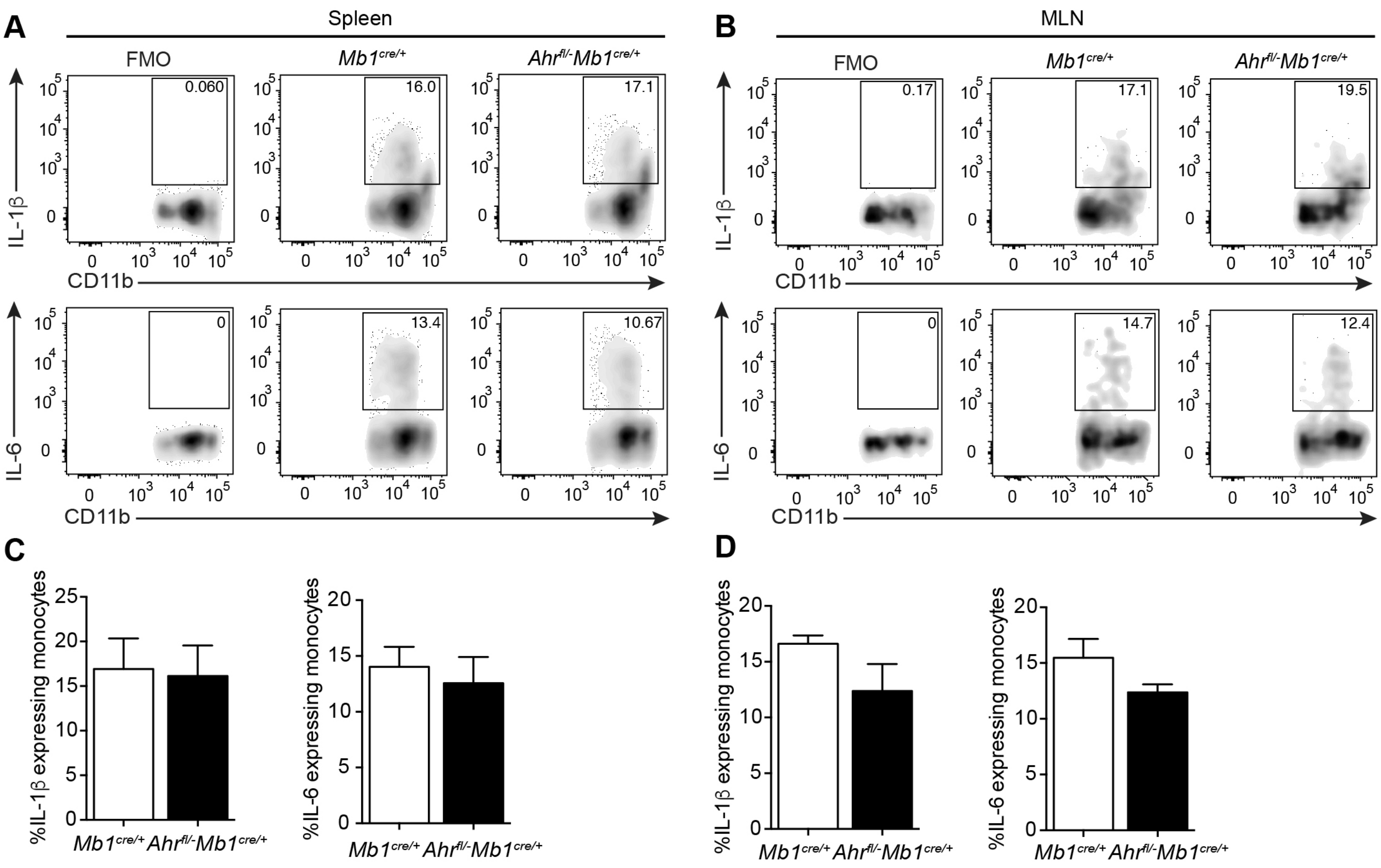

Figure S7 (Related to Figure 6): A-D, No difference in monocyte IL-1β and IL-6 expression is observed between *Mb1*$^{cre/+}$ and *Ahr*$^{fl/-}$*Mb1*$^{cre/+}$ mice. Total splenocytes or MLN cells were cultured for 6h with LPS. **A-D,** Representative flow cytometry plots and bar charts showing respectively the percentage of (**A,C**) splenic and (**B,D**) MLN IL-1β and IL-6-expressing monocytes (*n*=5). All data representative of at least 2 independent experiments with biological replicates. Figures **C-D,** data expressed as mean±sem. $^{**}p$<0.01

Symbol	Name	Function	FC ($CD21^{hi}CD24^{hi}$ pos vs $CD21^{hi}CD24^{hi}$ neg)	adj.P.Val ($CD21^{hi}CD24^{hi}$ pos vs $CD21^{hi}CD24^{hi}$ neg)	FC ($CD21^{hi}CD24^{hi}$ pos vs FO)	adj.P.Val ($CD21^{hi}CD24^{hi}$ pos vs FO)
Ahr	Aryl-hydrocarbon receptor	DNA binding	1.869114565	5.05135E-05	5.183739908	1.73543E-08
E2f8	E2F transcription factor 8	Core promoter binding	3.524122031	8.24144E-05	9.937468163	1.16556E-07
Bhlhe41	Basic helix-loop-helix family, member e41	RNA polymerase II core promoter proximal region sequence-specific DNA binding	3.151182551	5.20865E-05	5.070827978	2.34121E-07
Pim1	Proviral integration site 1	Nucleotide binding	1.43710652	0.000803313	1.536952061	5.28079E-05
Tacc3	Transforming, acidic coiled-coil containing protein 3	Protein binding	1.754219566	0.000237504	2.612848846	1.13543E-06
E2f7	E2F transcription factor 7	Core promoter binding	1.569164933	0.000491558	2.089170639	3.35506E-06
Dnmt1	DNA methyltransferase (cytosine-5) 1	DNA binding	1.525927763	0.001016079	1.542642041	0.000192334
Zbtb32	Zinc finger and BTB domain containing 32	Nucleic acid binding	1.564102074	0.00060818	1.599906949	8.54549E-05
Zfpm1	Zinc finger protein, multitype 1	RNA polymerase II core promoter binding transcription factor activity	1.635156815	0.000956596	2.073406035	1.36055E-05
Pmf1	Polyamine-modulated factor 1	Transcription coactivator activity	1.613117282	0.000743998	2.03500123	9.61821E-06
C1qbp	C1q binding protein	Complement component C1q binding	1.49365815	0.002166338	1.796509212	4.16981E-05
Foxm1	Forkhead box M1	DNA binding	1.457277204	0.00190059	2.20317842	3.01442E-06

Cenpf	Centromere protein F	Protein C-terminus binding	1.922637351	0.008852351	3.81234672	2.613E-05
Pdlim1	PDZ and LIM domain 1 (elfin)	Transcription coactivator activity	1.538439454	0.001738439	1.372627344	0.00277832
Setd8	SET domain containing (lysine methyltransferase) 8	P53 binding	1.481768428	0.005872492	1.58624982	0.000660724
E2f1	E2F transcription factor 1	Core promoter binding	1.27720107	0.005234129	1.799717751	4.40902E-06
Hes6	Hairy and enhancer of split 6	DNA binding	1.26830407	0.004558499	1.367381679	0.000227666
Smarca4	SWI/SNF related, matrix associated, actin dependent regulator of chromatin	Nucleotide binding	1.335143927	0.011950492	1.398775214	0.00168523
Dip2c	DIP2 disco-interacting protein 2 homolog C (Drosophila)	Unknown	1.258003811	0.02122796	1.734098658	2.86597E-05
Skil	SKI-like	Chromatin binding	-1.201268078	0.022180352	-1.54986752	3.18895E-05
Hhex	Hematopoietically expressed homeobox	DNA binding	-1.235422164	0.026407274	-1.511651527	0.000170022
Rbpms	RNA binding protein gene with multiple splicing	Nucleotide binding	-1.326003662	0.040745097	-1.992794467	7.70317E-05
Hist1h4k	Histone Cluster 1 H4 Family Member K	Unknown	1.274	0.019929	1.5213	0.00027

Table S1 (Related to Figure 2): List of 23 candidate genes differentially expressed between $CD21^{hi}CD24^{hi}IL\text{-}10eGFP^{+}$ and GFP^{-} populations. Abbreviations: FC – fold change, FO – Follicular.

Resource	Source	Identifier
**qPCR primers**		
Actb	ThermoFisher Scientific; This paper	N/A
Fwd 5'-AGATGACCCAGATCATGTTTGAG		
Rev 5'-AGGTCCAGACGCAGGATG		
Ahr	ThermoFisher Scientific; This paper	N/A
Fwd 5'-AGGATCGGGGTACCAGTTCA-3'		
Rev 5'-CTCCAGCGACTGTGTTTTGC-3'		
Ahrr	Qiagen	Cat#QT00161693
N/A		
Arnt	Qiagen	Cat#QT00151718
N/A		
Ccl22	ThermoFisher Scientific; Hao *et al*., 2016	N/A
Fwd 5'-CAGGCAGGTCTGGGTGAA-3'		
Rev 5'-TAAAGGTGGCGTCGTTGG-3'		
Cyp1a1	Qiagen	Cat#QT00105756
N/A		
Ebi3	ThermoFisher Scientific; Shen et al., 2014	N/A
CGGTGCCCTACATGCTAAAT		
GCGGAGTCGGTACTTGAGAG		
Il2	ThermoFisher Scientific; Martins., 2008	N/A
5′-AGCAGCTGTTGATGGACCTA-3′		
5′-CGCAGAGGTCCAAGTTCAT-3′		
Il5ra	ThermoFisher Scientific; This paper	N/A
Fwd 5'-GGTCCCGGTATGCAGTTCTA-3'		
Rev 5'-AGCCGAATGCTGGAAAAGTG-3'		
Il6	ThermoFisher Scientific; This paper	N/A
Fwd 5'-GCCTTCTTGGGACTGATGCT-3'		
Rev 5'-TGCCATTGCACAACTCTTTTC-3'		
Il10	ThermoFisher Scientific; Yanaba et al., 2009	N/A
Fwd 5'-GGTTGCCAAGCCTTATCGGA-3'		
Rev 5'-ACCTGCTCCACTGCCTTGCT-3'		
p35	ThermoFisher Scientific; Shen et al., 2014	N/A
Fwd 5'-CATCGATGAGCTGATGCAGT-3'		
Rev 5'-CAGATAGCCCATCACCCTGT-3'		
Tnf	ThermoFisher Scientific; Denaes et al., 2016	N/A
Fwd 5'-AATGGCCTCCCTCTCATCAGTT-3'		
Rev 5'-CCACTTGGTGGTTTGCTACGA-3'		
**ChIP qPCR primers**		

***Il10* -3.5kb**	ThermoFisher Scientific; This paper	N/A
Fwd 5'-AGGGCTTGATAACGTGTGAGT-3'		
Rev 5'-TGAACTTCACACCCAGCTTGAG-3'		
***Il10* -2kb**	ThermoFisher Scientific; This paper	N/A
Fwd 5'-TAAGAGGTGCTGCTTCTCCTG-3'		
Rev 5'-TGGCACTGGACAGTTCTATGA-3'		
***Il10* -0.5kb**	ThermoFisher Scientific; This paper	N/A
Fwd 5'-AGGGAGGAGGAGCCTGAATAA-3'		
Rev 5'-CCTGTTCTTGGTCCCCCTTTT-3'		
***Il10* +2kb**	ThermoFisher Scientific; This paper	N/A
Fwd 5'-GCCACATGCATCCAGAGACAC-3'		
Rev 5'-GTGCCTCAAAGTCACTCCCAC-3'		
***Cyp1a1* -3.6kb**	ThermoFisher Scientific; This paper	N/A
Fwd 5'-GCTCTTTCTCTGCCAGGTTG-3'		
Rev 5'-GGCTAAGGGTCACAATGGAA-3'		
***Cyp1a1* promoter**	ThermoFisher Scientific; This paper	N/A
Fwd 5'-AAGCATCACCCTTTGTAGCC-3'		
Rev 5'-CAGGCAACACAGAGAAGTCG-3'		
***Gapdh* promoter**	ThermoFisher Scientific; This paper	N/A
Fwd 5'-GCGCGAAAGTAAAGAAAGAAGCCC-3'		
Rev 5'-AGCGGCCCGGAGTCTTAAGTATTAG-3'		

Table S2. qPCR and ChIP qPCR primers used in this study. Related to the Key Resources Table in STAR methods. N/A – not applicable.
